# Efficacy and Safety of a Novel Triple Single-Pill for Uncontrolled Hypertension: The OPTION TREAT Trial

**DOI:** 10.1016/j.jacadv.2025.102175

**Published:** 2025-08-29

**Authors:** Vagner Madrini, Caio A.M. Tavares, Monica T.A. Albuquerque, Odilson M. Silvestre, João S. Felicio, Fabio S. Silveira, Miguel N. Hissa, Murillo O. Antunes, Marco A. Mota-Gomes, Maria H. Vidotti, Flávio D. Fuchs, Fabiana G. Marcondes-Braga, Conrado R.H. Filho, Charlene Troiani do Nascimento, Rafael A. Bastos, Andrea A. Brandão, Elizabeth E.S. Cestário, Luiz A. Bortolotto, Vivienne C. Castilho, Maura G. Lapa, Erida A.P. Magaton, Paula B. Fernandes, Camila S.N. Albuquerque, Lucas R. Silva, Otávio Berwanger, Patricia O. Guimarães

**Affiliations:** a Hospital Israelita Albert Einstein, São Paulo, Brazil; b Instituto do Coração (InCor), Hospital das Clinicas HCFMUSP, Faculdade de Medicina, Universidade de São Paulo, São Paulo, Brazil; c Universidade Federal do Acre, Rio Branco, Brazil; d João de Barros Barreto University Hospital, Federal University of Pará, Belém, Brazil; e Centro de Pesquisa Clínica do Coração, Aracaju, Brazil; f Centro de Pesquisas em Diabetes e Doenças Endócrino-Metabólicas/Clínica Popular Endocrinologia, Fortaleza, Brazil; g Hospital Universitário São Francisco de Assis na Providência de Deus da Universidade de São Francisco, Bragança Paulista, Brazil; h Centro Universitário CESMAC, Maceió, Brazil; i Centro I9 Pesquisa Clínica, Campinas, Brazil; j Hospital de Clínicas de Porto Alegre, Porto Alegre, Brazil; k Centro, CIPES, São José dos Campos, Brazil; l W&H Cardiologia, Joinville, Brazil; m Hospital Regional de Presidente Prudente, Presidente Prudente, Brazil; n Centro Ruy Azeredo, Goiânia, Brazil; o Faculdade de Ciências Médicas-Universidade do Estado do Rio de Janeiro, Rio de Janeiro, Brazil; p Clínica Cardiológica, Votuporanga, Brazil; q Libbs Pharmaceuticals, São Paulo, Brazil; r George Institute for Global Health UK, London, United Kingdom; s Imperial College London, London, United Kingdom

**Keywords:** blood pressure control, fixed-dose combination, hypertension, single-pill combination, triple therapy

## Abstract

**Background:**

Novel single-pill combinations with blood pressure (BP)-lowering agents are needed to increase treatment options for hypertension.

**Objectives:**

The purpose of this study was to assess the efficacy and safety of a novel single pill (candesartan cilexetil, amlodipine, and chlorthalidone) compared with an active control (valsartan, amlodipine, and hydrochlorothiazide) for uncontrolled hypertension.

**Methods:**

OPTION TREAT (Efficacy and Safety of a Novel Triple Single-Pill Combination Therapy Compared with an Active Control in Patients with Uncontrolled Hypertension) was a randomized, double-blind, double-dummy, noninferiority trial conducted across 19 sites in Brazil. Participants with an office systolic BP of 140 to 180 mm Hg and a diastolic BP of 90 to 110 mm Hg despite dual therapy were randomized in a 1:1 ratio to receive either the experimental treatment or the active control for 12 weeks. The primary outcome was the mean change in office systolic BP from baseline to week 12. The prespecified noninferiority margin was 3 mm Hg. Secondary outcomes included mean changes in diastolic BP and adverse events.

**Results:**

Overall, 703 participants were included (mean age 57.8 years, 62.7% women, baseline office BP of 153.0/95.6 mm Hg). At 12 weeks, the least square mean change in systolic BP was −22.6 mm Hg in the experimental group vs −18.2 mm Hg in the control group (between-group difference −4.4 mm Hg; 90% CI –6.3 to −2.5 mm Hg; *P* < 0.001). Diastolic BP was also reduced in both groups, with greater reductions in the experimental group (−13.8 mm Hg vs −12.0 mm Hg; *P* = 0.008). Adherence was high, and serious adverse events were rare.

**Conclusions:**

In patients with uncontrolled hypertension, a novel triple single-pill containing candesartan cilexetil, amlodipine, and chlorthalidone improved BP control at 12 weeks and had a reasonable safety profile. (Candesartan Cilexetil + Chlorthalidone + Amlodipine Versus Exforge HCT for Systemic Arterial Hypertension [OPTION TREAT]; NCT05920005)

Hypertension is a global public health issue, affecting over one billion individuals worldwide and contributing significantly to the burden of cardiovascular diseases.^1^ Most patients with hypertension require a combination of two or more antihypertensive agents to achieve optimal blood pressure (BP) control.^2^^,^^3^ Despite the availability of effective therapies, challenges in managing patients with hypertension persist. In low- and middle-income countries, socioeconomic barriers influence patient access and adherence to treatments. In Latin America, only a third of patients receiving medications for hypertension achieve BP control targets.^4^ Moreover, the social impact of hypertension extends beyond clinical outcomes, imposing substantial costs on health care systems.^5^

Single-pill combinations (SPCs) of two or more BP-lowering agents offer a pragmatic approach to simplify treatment regimens in resource-constrained settings.^6^^,^^7^ Such combinations have demonstrated significant improvements in BP control and enhanced patient adherence, ultimately reducing cardiovascular risk.^8^^,^^9^ However, the availability of drug combinations in a single-pill formulation is limited.^9^ The development of novel SPCs comprising different agents may help expand therapeutic options for hypertension management.

The combination of a calcium-channel blocker, an angiotensin receptor antagonist, and a thiazide diuretic is attractive due to their complementary mechanisms of action and potential for mitigating side effects associated with each drug class.^10^, ^11^, ^12^ Although candesartan cilexetil, amlodipine, and chlortalidone are individually effective and frequently used together in clinical practice, the efficacy and tolerability of a triple pill containing these 3 components have not been established. We therefore designed a randomized trial to evaluate the efficacy and safety of a novel triple combination containing candesartan cilexetil 16 mg, chlorthalidone 12.5 mg, and amlodipine 5 mg vs an active comparator of established BP-lowering effects (single pill of valsartan 160 mg, hydrochlorothiazide 12.5 mg, and amlodipine 5 mg) in adults with uncontrolled hypertension (HTN).

## Methods

### Study design and participants

The OPTION TREAT (Efficacy and Safety of a Novel Triple Single-Pill Combination Therapy Compared with an Active Control in Patients with Uncontrolled Hypertension) trial was a randomized, double-blind, double-dummy, active-controlled, parallel-group study conducted across 19 centers in Brazil. Libbs Pharmaceuticals funded the trial, which was designed and conducted in collaboration with the Academic Research Organization of the Hospital Israelita Albert Einstein. The scientific committee and sponsor jointly supervised the trial conduct. The Brazilian Health Regulatory Agency and the Institutional Review Boards of all participating sites approved the protocol. All participants provided written informed consent prior to any study activity. An independent Data and Safety Monitoring Board reviewed patient-level data during the study, including 2 prespecified safety analyses after approximately 25% and 50% of participants completed study treatment. The protocol and statistical analysis plan are provided in the Supplemental Appendix.

Eligible participants were adults with uncontrolled hypertension (office systolic BP [SBP] between 140 and 180 mm Hg and diastolic BP [DBP] between 90 and 110 mm Hg) despite dual antihypertensive therapy for ≥8 weeks before screening. Between screening and randomization, participants continued receiving their usual dual antihypertensive treatment and underwent laboratory assessments. At the randomization visit, BP eligibility was confirmed if SBP and DBP were within the same predefined ranges. Key exclusion criteria included recent major cardiovascular events (myocardial infarction, stroke, or heart failure hospitalization), renal impairment (estimated glomerular filtration rate <45 mL/min/1.73 m^2^), severe hepatic dysfunction, symptomatic heart failure, pregnancy or lactation, hypersensitivity to study medications, and known obstructive coronary artery disease. Complete eligibility criteria are provided in Supplemental Table 1.

### Randomization and interventions

Participants were randomized in a 1:1 ratio via a centralized web-based system to stop their prior dual antihypertensive therapy and receive either the experimental treatment (candesartan cilexetil 16 mg/chlorthalidone 12.5 mg/amlodipine 5 mg) or the active control (valsartan 160 mg/hydrochlorothiazide 12.5 mg/amlodipine 5 mg) for 12 weeks. An independent statistician generated the allocation sequence. The study design is shown in Supplemental Figure 1.

Due to different formulation appearances, a double-dummy design was employed. All participants received one active tablet plus one matching placebo capsule at approximately the same time in the morning. The intervention group received the experimental treatment plus control-matched placebo; the control group received the comparator product plus intervention-matched placebo. All personnel and participants remained blinded throughout the trial. No emergency unblinding occurred.

### Study procedures

The trial included screening, randomization (1 week after screening), and follow-up visits at weeks 4, 8, and 12 after randomization (Supplemental Figure 2). BP was measured following international guidelines.^13^ After 5 minutes of seated rest, 3 readings were obtained using a standardized automated device (HEM 7122, Omron Healthcare) at 1- to 2-minute intervals that was provided to all participating sites by the sponsor. If any 2 systolic readings differed by >10 mm Hg, additional measurements were taken until variability was below this threshold. The mean of the last 2 valid readings was used to calculate the SBP and DBP in each visit. Laboratory assessments were collected at screening, week 4, and week 8 visits, which included renal function (creatinine, estimated glomerular filtration rate), electrolytes (sodium, potassium), liver enzymes, complete blood count, and urinalysis. Adherence was assessed by pill count at each visit after randomization. A safety telephone follow-up occurred 30 days after the last in-person visit.

## Outcomes

The primary efficacy outcome was the between-group difference in the mean change in office SBP from baseline to week 12. Secondary efficacy outcomes included mean changes in SBP and DBP at weeks 4 and 8; changes in DBP at week 12; the proportion of participants achieving BP control rates (<140/90 mm Hg or SBP <120 mm Hg); and the proportion with clinically significant BP reductions (≥20 mm Hg in SBP and/or ≥10 mm Hg in DBP) at weeks 4, 8, and 12.

Safety outcomes included the proportion of patients experiencing adverse events, adverse events leading to treatment discontinuation, and serious adverse events (defined as events that resulted in death, were life-threatening, required hospitalization or prolongation of an existing hospitalization, resulted in persistent or significant disability or incapacity, or caused a birth defect). Clinically relevant abnormalities in laboratory parameters, vital signs, and physical examination findings were other safety outcomes. All outcomes were prespecified in the protocol and statistical analysis plan (Supplemental Appendix) and summarized in Supplemental Table 2.

### Statistical analysis

We estimated that a sample size of 698 participants would provide 85% power to demonstrate the noninferiority of the intervention over the active control based on an assumed SD of 14 mm Hg for the mean change in SBP, a noninferiority margin of 3 mm Hg, a one-sided type I error rate of 0.05, and an anticipated dropout rate of approximately 10%.^14^^,^^15^ The 3 mm Hg noninferiority margin was aligned with previous studies.^16^^,^^17^

All efficacy analyses were performed according to the statistical analysis plan finalized before database lock (Supplemental Appendix). The primary analysis followed the intention-to-treat principle and included all randomized participants who contributed with data for at least 2 follow-up visits, with no imputation for missing data. The primary efficacy outcome was analyzed using a linear mixed-effects model for repeated measures with fixed effects for treatment group, visit, treatment-by-visit interaction, and baseline SBP as a covariate. A random effect for study site accounted for site-level clustering. The model was fitted using the restricted maximum likelihood method. Noninferiority would be declared if the upper bound of the one-sided 90% CI for the treatment difference was below or equal to 3.0 mm Hg.

A sensitivity analysis for the primary efficacy outcome was performed using multiple imputation for missing data. A pattern mixture model approach was implemented with 50 imputations using the multiple imputation procedure in SAS (SAS Institute). For participants who permanently discontinued study treatment due to use of another antihypertensive medication not permitted by the protocol, data were imputed using the last observation carried forward method from week 8 onward, as these data were classified as missing not at random. Intermittent missing values were imputed using Markov chain Monte Carlo methodology assuming a multivariate normal distribution and missing at random assumption. Missing data from dropouts were imputed using a sequential procedure for monotonic data, incorporating treatment group, baseline BP, and other covariates from the analysis model. Results from the 50 imputed data sets were combined using Rubin's rule to obtain final parameter estimates and CIs. Two post hoc sensitivity analyses for the primary outcome were also performed: 1) without baseline SBP as a covariate; and 2) including all participants who had at least one follow-up visit with BP data available. Both used the same mixed-effects model structure as the primary analysis.

Secondary continuous outcomes were analyzed using the similar linear mixed-effects models. Categorical outcomes, including the proportion of participants achieving BP control at predefined thresholds, were analyzed using chi-square tests. The safety population included participants who received at least 1 dose of study treatment. No adjustment for multiplicity was performed, and *P* values for secondary and exploratory endpoints are presented descriptively. All analyses were performed using SAS software, version 9.4 (SAS Institute).

## Results

**Figure 1:**
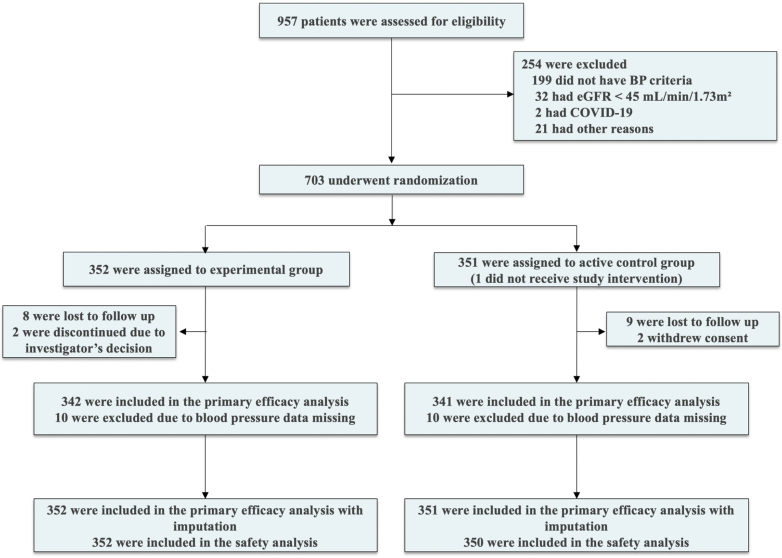
**Patient Flowchart** The primary efficacy analysis population included all patients who underwent randomization and had blood pressure data for at least 2 study follow-up visits. The safety population included all participants who receive at least 1 dose of study drug. One participant was of the active control group did not receive study treatment. BP = blood pressure; eGFR = estimated glomerular filtration rate.

Between August 2023 and December 2024, a total of 957 individuals were screened for eligibility, of whom 703 were randomized to receive either the experimental treatment (n = 352) or the active control (n = 351) (Figure 1). The main reasons for screen failure were not meeting the BP eligibility criteria, having an estimated glomerular filtration rate lower than 45 mL/min/1.73 m^2^, and having suspected or confirmed COVID-19 infection. All participants allocated to the experimental group received the assigned treatment, whereas one participant in the active control group did not receive the allocated intervention. By the end of follow-up in April 2025, 679 participants (96.6%) completed the trial: 339 participants in the experimental treatment group and 340 in the active control group. Overall, 342 patients in the experimental treatment group and 341 in the active control group had BP data available for at least 2 follow-up visits and were included in the primary efficacy analysis. A total of 21 participants discontinued the study: 17 were lost to follow-up, 2 withdrew consent, and 2 were discontinued due to the investigator’s decision.

### Baseline characteristics

Baseline characteristics were well balanced between the study groups (Table 1). The mean age was 57.8 years, 62.7% were women, and 62.4% were Black or mixed race. The mean body mass index was 31.1 kg/m^2^. At randomization, the mean SBP/DBP was 153.0/95.6 mm Hg. A total of 39.4% had diabetes and 49.9% dyslipidemia. Overall, 95.0% of participants were previously treated with renin-angiotensin system blockers and 59.1% with thiazide diuretics before randomization.

**Table 1:** Baseline Characteristics of Randomized Participants

	Experimental Group (n = 352)	Active Control Group (n = 351)	Total (N = 703)
Age, y	57.6 ± 11	57.9 ± 10.5	57.75 ± 10.75
Female	227 (64.5%)	214 (61.0%)	441 (62.7%)
Race			
White	120 (34.1%)	138 (39.4%)	258 (36.7%)
Black	65 (18.5%)	63 (18.0%)	128 (18.2%)
Mixed race	163 (46.3%)	148 (42.3%)	311 (44.3%)
Asian	4 (1.1%)	0 (0.0%)	4 (0.6%)
Indigenous	0 (0.0%)	1 (0.3%)	1 (0.1%)
BMI, kg/m^2^	30.8 ± 5.7	31.5 ± 5.8	31.15 ± 5.75
Hypertension status			
SBP at screening, mm Hg	156.3 ± 11.3	157.4 ± 11.4	156.9 ± 11.35
DBP at screening, mm Hg	96.2 ± 5.3	96.6 ± 5.6	96.4 ± 5.45
SBP at randomization, mm Hg	152.8 ± 10.7	153.1 ± 10.9	153.0 ± 10.8
DBP at randomization, mm Hg	95.5 ± 5.3	95.6 ± 5.4	95.6 ± 5.35
Health conditions			
Type 2 diabetes mellitus	142 (40.3%)	135 (38.5%)	277 (39.4%)
Dyslipidemia	180 (51.3%)	171 (48.6%)	351 (49.9%)
Coronary artery disease	4 (1.1%)	14 (4.0%)	18 (2.6%)
Stroke	3 (0.9%)	7 (2.0%)	10 (1.4%)
Alcohol use	58 (16.5%)	65 (18.5%)	123 (17.5%)
Smoking			
Never	257 (73.0%)	267 (76.1%)	524 (74.6%)
Former	79 (22.4%)	69 (19.7%)	148 (21.1%)
Current	16 (4.5%)	15 (4.3%)	31 (4.4%)
Medications			
Renin-angiotensin system blockers	330 (93.8%)	338 (96.3%)	668 (95.0%)
Thiazide diuretics	194 (55.1%)	222 (63.2%)	416 (59.1%)
Calcium-channel blockers	102 (29%)	77 (21.9%)	179 (25.3%)
Beta-blockers	61 (17.3%)	53 (15.1%)	114 (16.2%)
Mineralocorticoid receptor antagonists	1 (0.3%)	1 (0.3%)	2 (0.3%)

Values are mean ± SD or n (%).

BMI = body mass index; DBP = diastolic blood pressure; SBP = systolic blood pressure.

### Primary outcome

**Figure 2:**
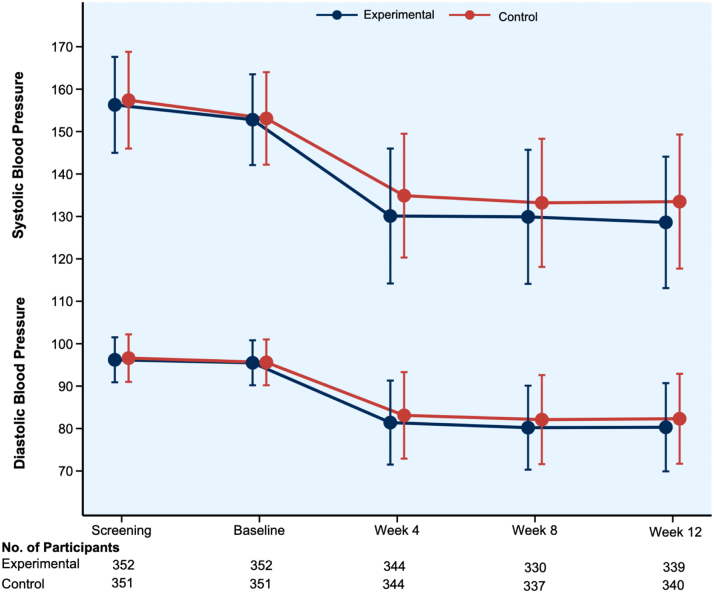
**Office Mean Blood Pressure During the Course of the Trial by Treatment Groups** The mean systolic and diastolic blood pressure at screening, baseline, and follow-up visits are presented by study groups. The SD at each visit is shown. The trajectory of systolic and diastolic BP over 12 weeks demonstrated blood pressure reductions in both treatment arms, with a greater absolute reduction observed in the experimental treatment group. Abbreviation as in Figure 1.

At 12 weeks, mean SBP was 128.6 ± 15.5 mm Hg in the experimental group and 133.5 ± 15.8 mm Hg in the active control group (Table 2, Figure 2). The least square mean changes in SBP from baseline to week 12 were −22.6 mm Hg (SE: 1.90) in the experimental group and −18.2 mm Hg (SE: 1.90) in the active control group (between-group difference −4.4 mm Hg [90% CI: −6.3 to −2.5 mm Hg], *P* < 0.001) (Central Illustration). Results were consistent in the sensitivity analysis using multiple imputation (between-group difference of −4.6 mm Hg [95% CI: −6.8 to −2.4 mm Hg]; *P* < 0.001) and in post hoc analyses without baseline SBP adjustment and including all participants with ≥1 follow-up visit (Supplemental Table 3).

**Table 2:** Primary and Secondary Outcomes

Primary Outcome	Experimental Group (n = 342)	Active Control Group (n = 341)	Mean Difference (90% CI)	*P* Value
Change in SBP from baseline to 12 wk	−22.6 ± 1.9	−18.2 ± 1.9	−4.4 (−6.3 to −2.5)	<0.001^a^

Key Secondary Outcomes			Mean Difference (95% CI)^b^	
Change in SBP from baseline to wk 4	−22.7 ± 15.2	−18.4 ± 14.1	−4.30 (−6.6 to −2.1)	<0.001
Change in SBP from baseline to wk 8	−22.9 ± 16	−20.1 ± 15.2	−2.94 (−5.2 to −0.6)	0.012
Change in DBP from baseline to wk 4	−14.1 ± 8.7	−12.5 ± 8.9	−1.64 (−3.0 to −2.8)	0.018
Change in DBP from baseline to wk 8	−15.2 ± 9.3	−13.5 ± 9.1	−1.67 (−3.0 to −0.3)	0.017
Change in DBP from baseline to wk 12	−15.1 ± 9.2	−13.3 ± 9.3	−1.84 (−3.2 to −0.5)	0.008

BP = blood pressure; other abbreviations as in Table 1.

Blood pressure measurements are presented in mm Hg.

a The primary efficacy outcome was analyzed using a linear mixed-effects model for repeated measures. The noninferiority margin was set at 3 mm Hg. *P* value for noninferiority is presented.

b The widths of the 95% CIs for secondary outcomes were not adjusted for multiple comparisons and should not therefore be used for inference about treatment effects.

**Central Illustration:**
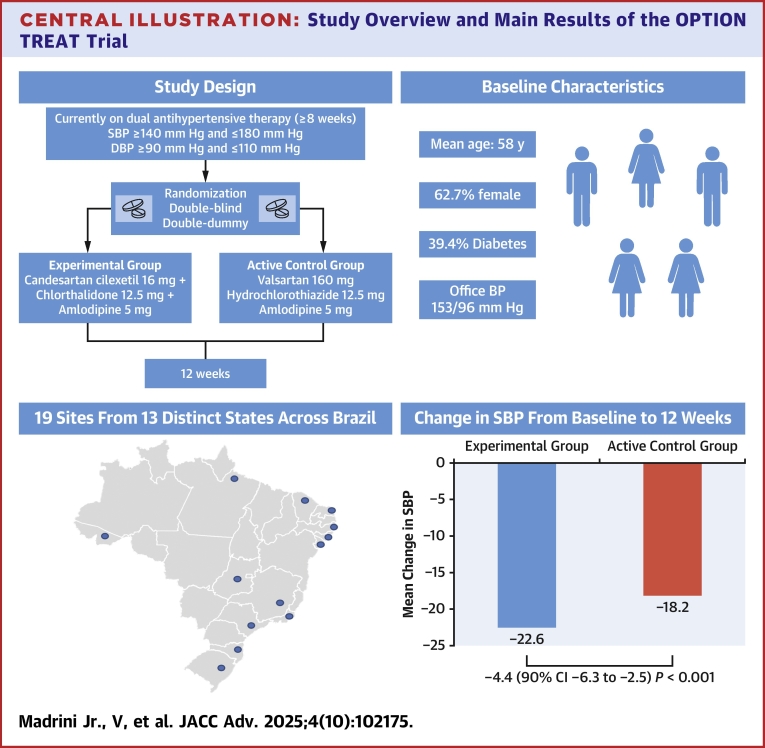
**Study Overview and Main Results of the OPTION TREAT Trial** This figure summarizes key baseline characteristics and blood pressure outcomes of participants enrolled in the OPTION TREAT trial. OPTION TREAT = Efficacy and Safety of a Novel Triple Single-Pill Combination Therapy Compared with an Active Control in Patients with Uncontrolled Hypertension; other abbreviations as in Figures 1 and 3.

### Secondary outcomes

At week 12, both treatment groups showed significant reductions in DBP compared with baseline. The least squares mean changes in DBP were −13.84 mm Hg (SE: 1.03) in the experimental group and −12.00 mm Hg (SE: 1.03) in the active control group (between-group difference −1.84 mm Hg [90% CI: −3.0 to −0.7 mm Hg], *P* = 0.008).

**Figure 3:**
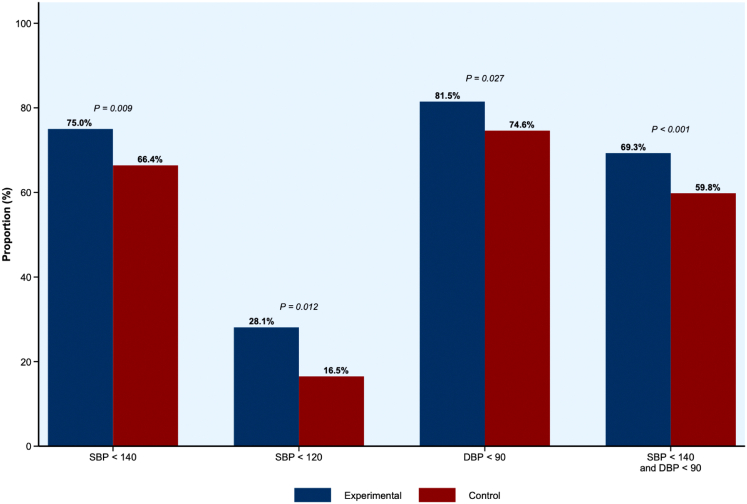
**Proportion of Patients Achieving Blood Pressure Targets at Week 12** The proportions of patients achieving blood pressure targets at week 12 were compared using chi-square tests. DBP = diastolic blood pressure; SBP = systolic blood pressure.

The proportion of patients achieving BP targets at weeks 4, 8, and 12 are presented in the Supplemental Table 4. A higher proportion of participants in the experimental group achieved BP < 140/90 mm Hg at week 12 (69.3% vs 59.8%; *P* = 0.009) (Figure 3). Additionally, SBP< 120 mm Hg was achieved in 28.1% of patients in the experimental group compared to 16.5% in the active control group (*P* < 0.001). SBP <140 mm Hg was achieved in 75.0% vs 66.4% (*P* = 0.012) of the experimental group vs the active control, respectively. Other secondary outcomes are presented in Table 2. Overall, 93.5% of participants in the experimental group and 93.8% in the control group had treatment adherence between 80% and 120% of the time (Table 3).

**Table 3:** Adherence to Study Treatments

	Experimental Group (n = 352)	Active Control Group (n = 351)	Total (N = 703)
<50%	9 (2.6%)	8 (2.3%)	17 (2.4%)
50%-79%	14 (4.0%)	14 (4.0%)	28 (4.0%)
80%-99%	297 (84.4%)	298 (84.9%)	595 (84.6%)
100%-119%	28 (8.0%)	28 (8.0%)	56 (8.0%)
≥120%	4 (1.1%)	3 (0.9%)	7 (1.0%)

Adherence to study treatment across study visits. Adherence was calculated as (actual amount of use × 100)/expected amount of use based on pill count. Data are presented as n (%) of participants within each adherence category. Percentages are calculated based on the total safety population at each visit.

### Safety

Overall, 31.5% of participants in the experimental treatment group and 30.9% of those in the active control group reported at least one adverse event (Table 4). Most adverse events were considered as mild or moderate in intensity. The most common adverse event reported was hypotension (2.9%), followed by dizziness (2.6%) and peripheral edema (2.1%). Other adverse events and laboratory changes were rare. A total of 15 patients in the experimental treatment group (4.3%) and 8 in the active control groups (2.3%) reported serious adverse events; in each group, 2 serious adverse events were considered related to the study treatment. Overall, 3 patients (0.9%) in the active control group presented with adverse events leading to treatment discontinuation. One patient in the active control group died of cancer during trial follow-up.

**Table 4:** Adverse Events by Treatment Groups

Adverse Events	Experimental Group (n = 352)	Active Control Group (n = 350)	Total (N = 702)
At least one adverse event	111 (31.5)	108 (30.9)	219 (31.2)
At least one serious adverse event	15 (4.3)	8 (2.3)	23 (3.3)
At least one serious adverse event related to study treatment	2 (0.6)	2 (0.6)	4 (0.6)
At least one adverse event leading to treatment discontinuation	0 (0)	3 (0.9)	3 (0.4)
Most common adverse events			
Hypotension	11 (3.1)	9 (2.6)	20 (2.9)
Dizziness	10 (2.8)	8 (2.3)	18 (2.6)
Peripheral edema	4 (1.1)	11 (3.1)	15 (2.1)
Urinary infection	7 (2.0)	7 (2.0)	14 (2.0)
Headache	4 (1.1)	3 (0.9)	7 (1.0)
General discomfort	3 (0.9)	4 (1.1)	7 (1.0)

All other adverse events occurred in <1% of patients.

## Discussion

This randomized, double-blind, multicenter trial assessed the efficacy and safety of a novel triple pill containing candesartan cilexetil, amlodipine, and chlorthalidone in patients with uncontrolled hypertension. The experimental treatment was effective in reducing SBP at 12 weeks compared with the active control. Tolerability was good with low occurrence of serious adverse events and low rates of treatment discontinuation. This trial introduces a novel triple SPC that may be an effective strategy for the management of patients with high BP levels despite the use of dual antihypertensive therapy.

Several trials have shown that triple SPCs provide greater BP-lowering effects than dual therapies, usual care, or placebo in patients with initial, moderate, or severe HTN.^11^^,^^12^^,^^15^^,^^16^^,^^18^, ^19^, ^20^ However, not all antihypertensive agents are available in single-pill formulations. Pharmacological evidence suggests synergistic effects of candesartan cilexetil, amlodipine, and chlortalidone with the potential of enhancing BP reduction.^10^^,^^11^^,^^21^ Although these 3 components are effective BP-lowering agents, their use in combination in a single pill had not been examined previously in a randomized clinical trial. Since patients had uncontrolled BP levels at baseline with use of dual therapy, an established triple SPC comprising agents from the same pharmacologic classes was chosen as the active control for this study. At 12 weeks, both study groups showed significant mean SBP reductions (∼20 mm Hg), which was sufficient to declare the noninferiority of the study treatment over the SPC comparator. Other trials showed similar BP-lowering effects with triple pills containing other components from the same drug classes.^11^^,^^22^ Conversely, greater SBP reductions with three-drug combinations were seen in patients with higher baseline BP levels.^15^^,^^18^ In our trial, substantial reductions in DBP were also observed with study treatments. The overall magnitude of BP control seen in OPTION TREAT was clinically meaningful, especially considering our study population presented with a baseline SBP of ∼153 mm Hg with prior use of dual antihypertensive therapy.

In our study, the most substantial BP-lowering effect was seen within the first 4 weeks of study treatment, and after that, mean BP levels were maintained through week 12. Trials evaluating other triple SPCs found similar patterns of BP reduction over time.^12^^,^^23^^,^^24^These studies demonstrated SBP reductions within the first weeks of treatment in up to 70% of patients, without an increase in adverse events.^19^ A meta-analysis of 7 trials of low-dose SPCs containing 3 or 4 agents also observed that BP reduction was more pronounced at early follow-up.^23^ In OPTION TREAT, two-thirds of participants receiving the experimental treatment achieved BP control (<140/90 mm Hg) at 12 weeks, which was higher than the observed in those receiving the active control. Other studies showed similar rates of BP control over 12 weeks among patients treated with SPCs containing aldosterone receptor blockers, amlodipine, and diuretics.^12^^,^^18^

One major aspect of developing SPC therapies is establishing safety. In OPTION TREAT, the overall incidence of adverse events was low and balanced between treatment groups. The most frequent adverse events were hypotension, dizziness, and peripheral edema, each occurring in < 3.0% of patients. Electrolyte abnormalities, such as hyponatremia and hyperkalemia, were also rare. Importantly, treatment discontinuation due to adverse events occurred in only 3 participants of the active control group. Other studies on triple combination therapies reported 4.0% to 5.0% rates of treatment discontinuation, depending on the dose of each component within the single pill.^18^^,^^19^

The use of SPCs has the potential to improve adherence by simplifying treatment regimens, reducing pill burden, and enhancing patient persistence. Gupta et al reported a 21% increase in medication compliance with combination therapies compared with the observed with their individual components given separately.^25^ Prior trials testing triple SPCs have reported treatment adherence of 59% to 77%,^24^ which is higher than the observed with multipill regimens.^24^^,^^26^^,^^27^ We also observed that over 80% of study participants adhered to study treatment throughout the trial.

### Study limitations

The trial was conducted exclusively in Brazil, which may limit the generalizability of findings to other regions with different genetic backgrounds, dietary patterns, and health care systems. In addition, the follow-up duration was restricted to 12 weeks. Although this period encompasses the typical window during which maximal antihypertensive effects are observed, it does not allow for long-term evaluation of treatment efficacy, safety, cardiovascular outcomes, or sustained adherence beyond this period. The study also did not include ambulatory or home BP monitoring, which could have provided greater insight into BP variability and nocturnal control. Finally, patients with very high BP levels at baseline were excluded; thus, no conclusions on the efficacy and safety of the experimental treatment on this population may be drawn.

## Conclusions

A novel triple-pill containing candesartan cilexetil, amlodipine, and chlorthalidone improved BP control at 12 weeks and had a reasonable safety profile in patients previously treated with dual therapy.

Perspectives **COMPETENCY IN MEDICAL KNOWLEDGE:** This randomized, double-blind, double-dummy, multicenter trial demonstrated that a novel triple SPC of candesartan cilexetil, amlodipine, and chlorthalidone significantly reduced systolic and diastolic BP in patients with uncontrolled hypertension compared with an active control containing valsartan, amlodipine, and hydrochlorothiazide. The experimental therapy achieved higher BP control rates with a good safety profile, underscoring its potential role as an effective treatment option in patients requiring escalation beyond dual therapy.

**TRANSLATIONAL OUTLOOK:** Despite guideline endorsements for SPCs to improve adherence and outcomes, the availability of triple combination formulations remains limited. The OPTION TREAT trial provides evidence supporting the efficacy and safety of a new triple single-pill in patients with uncontrolled hypertension, including a substantial proportion of women and individuals of Black or mixed race. Future long-term studies assessing cardiovascular outcomes, adherence, and cost-effectiveness across diverse health care settings are warranted to further define its place in hypertension management.

## Funding support and author disclosures

This study was funded by Libbs Pharmaceuticals. Dr Madrini Jr has received lecture fees from Bristol Myers Squibb, Novartis, and Bayer outside the submitted work. Dr Tavares has received lecture fees from Novo Nordisk outside the submitted work. Dr Berwanger has received research grants from Bayer paid to his current institution and research grants from AstraZeneca, Bayer, Servier, Amgen, Pfizer, and Novartis paid to his former institution. Drs Castilho, Lapa, Magaton, and Fernandes are employees of Libbs Pharmaceuticals. All other authors have reported that they have no relationships relevant to the contents of this paper to disclose.

## References

[1] Malta D.C., Santos N.B., Perillo R.D. (2016). Prevalence of high blood pressure measured in the Brazilian population, National Health Survey, 2013. Sao Paulo Med J.

[2] Volpe M., Gallo G., Tocci G. (2020). New approach to blood pressure control: triple combination pill. Trends Cardiovasc Med.

[3] Jamerson K.A., Brakis G.L., Wun C.C. (2008). Rationale and design of the avoiding cardiovascular events through combination therapy in patients living with systolic hypertension (ACCOMPLISH) trial. Am J Hypertens.

[4] Lames P., Dias R., Orlandini A. (2019). Prevalence, awareness, treatment and control of hypertension in rural and urban communities in Latin American countries. J Hyperten.

[5] Picon R.V., Fuchs F.D., Moreira L.B. (2012). Trends in prevalence of hypertension in Brazil: a systematic review with meta-analysis. PLoS One.

[6] Carey R.M., Whelton P.K. (2018). Prevention, detection, evaluation, and management of high blood pressure in adults. Ann Intern Med.

[7] Williams B., Mancia G., Spiering W. (2018). 2018 ESC/ESH guidelines for the management of arterial hypertension. Eur Heart J.

[8] Malachias M.V.B., Souza W.K.S.B., Plavnik F.L. (2016). Diretriz Brasileira de Hipertensão Arterial: Capítulo 1 - Conceituação, Epidemiologia e Prevenção Primária. Arquivos Brasileiros de Cardiologia.

[9] Wright J.T., Williamson J.D., Whelton P.K. (2015). A randomized trial of intensive versus standard blood-pressure control. N Engl J Med.

[10] Chi H., Zhang X., Ma S. (2025). Efficacy and safety of allisartan isoproxil/amlodipine in patients with essential hypertension uncontrolled by amlodipine: a phase III, multicenter, double-blind, parallel-group, randomized controlled trial. J Clin Hypertens.

[11] Cho E.J., Kim M.H., Kim Y.H. (2023). Efficacy and safety of standard dose triple combination of telmisartan 80 mg/amlodipine 5 mg/chlorthalidone 25 mg in primary hypertension: a randomized, double-blind, active-controlled, multicenter phase 3 trial. J Clin Hypertens (Greenwich).

[12] Rodgers A., Salam A., Schutte A.E. (2024). Efficacy and safety of a novel low-dose triple single-pill combination of Telmisartan, Amlodipine and Indapamide, compared with dual combinations for treatment of hypertension: a randomised, double-blind, active-controlled, international clinical trial. Lancet.

[13] Unger T., Borghi C., Charchar F. (2020). 2020 international society of hypertension global hypertension practice guidelines. Hypertension.

[14] Smith T.R., Glazer R.D., Koren M.J. (2010). Combination therapy with amlodipine/valsartan in essential hypertension: a 52-week, randomised, open-label, extension study. Int J Clin Pract.

[15] Calhoun D.A., Lacourcière Y., Chiang Y.T. (2009). Triple antihypertensive therapy with amlodipine, valsartan, and hydrochlorothiazide: a randomized clinical trial. Hypertension.

[16] Ojji D.B., Mayosi B., Francis V. (2019). Comparison of dual therapies for lowering blood pressure in black Africans. N Engl J Med.

[17] Sharma A.M., Davidson J., Koval S. (2007). Telmisartan/hydrochlorothiazide versus valsartan/hydrochlorothiazide in obese hypertensive patients with type 2 diabetes: the SMOOTH study. Cardiovasc Diabetol.

[18] Oparil S., Melino M., Lee J. (2010). Triple therapy with olmesartan medoxomil, amlodipine besylate, and hydrochlorothiazide in adult patients with hypertension: the TRINITY multicenter, randomized, double-blind, 12-week, parallel-group study. Clin Ther.

[19] Rodgers A., Salam A., Schutte A.E. (2024). Efficacy and safety of a novel low-dose triple single-pill combination compared with placebo for initial treatment of hypertension. J Am Coll Cardiol.

[20] Webster R., Salam A., de Silva H.A. (2018). Fixed low-dose triple combination antihypertensive medication vs usual care for blood pressure control in patients with mild to moderate hypertension in Sri Lanka: a randomized clinical trial. JAMA.

[21] Hong S.J., Sung K.C., Lim S.W. (2020). Low-dose triple antihypertensive combination therapy in patients with hypertension: a randomized, double-blind, phase II study. Drug Des Devel Ther.

[22] Lung T., Jan S., de Silva H.A. (2019). Fixed-combination, low-dose, triple-pill antihypertensive medication versus usual care in patients with mild-to-moderate hypertension in Sri Lanka: a within-trial and modelled economic evaluation of the TRIUMPH trial. Lancet Glob Health.

[23] Wang N., Rueter P., Atkins E. (2023). Efficacy and safety of low-dose triple and quadruple combination pills vs monotherapy, usual care, or placebo for the initial management of hypertension: a systematic review and meta-analysis. JAMA Cardiol.

[24] Wang X., Chen H., Essien E. (2019). Medication adherence to antihypertensive triple-combination therapy among patients enrolled in a medicare advantage plan. J Manag Care Spec Pharm.

[25] Gupta A.K., Arshad S., Poulter N.R. (2010). Compliance, safety, and effectiveness of fixed-dose combinations of antihypertensive agents: a meta-analysis. Hypertension.

[26] Rea F., Morabito G., Savaré L. (2023). Adherence and related cardiovascular outcomes to single pill vs. separate pill administration of antihypertensive triple-combination therapy. J Hypertens.

[27] Mapesi H., Rohacek M., Vanobberghen F. (2025). Treatment strategies to control blood pressure in people with hypertension in Tanzania and Lesotho: a randomized clinical trial. JAMA Cardiol.

