# Supplementary material for: Efficacy and Safety of a Novel Triple Single-Pill for Uncontrolled Hypertension: The OPTION TREAT Trial
Source: JACC Adv. 2025 Aug 29;4(10):102175. doi: 10.1016/j.jacadv.2025.102175 (PMC12791868; doi:10.1016/j.jacadv.2025.102175)
Supplement: Supplementary Material [file mmc1.pdf]

Supplementary Appendix to Manuscript Entitled:

Efficacy and Safety of a Novel Triple Single-Pill For Uncontrolled Hypertension: the OPTION TREAT Trial

TABLE OF CONTENTS

Study Investigators 3

OPTION-TREAT Study Group 5

Supplemental Tables 6

Supplemental Table S1. Inclusion and Exclusion Criteria 6

Supplemental Table S2. Study Outcomes 7

Supplemental Table S3. Post-hoc Sensitivity Analyses of the Primary Outcome 8

Supplemental Table S4. Proportion of Patients Achieving Blood Pressure Targets and Reductions at Weeks 4, 8, and 12 9

Supplemental Figures 10

Supplemental Figure S1. Study Design 10

Supplemental Figure S2. Study Procedures 11

Study Investigators

Participant Centers in Brazil and Site Investigators

1. Hospital Israelita Albert Einstein: Patrícia O. Guimarães, Vagner M. Junior. Caio A. M. Tavares (Coordinating Center)
2. Clínica Silvestre Santé - Centro de Pesquisa Silvestre Santé: Odilson Marcos Silvestre, Débora de Souza Rodrigures, Thais Giovana Araujo, Paula, Thais Giovana Araujo, Bárbara Vieira dos Santos, Hortência, Maysa Oliveira de Abreu Dias, Wilson Rodrigues Barbosa Neto. (Enrolled participants=132)
3. Hospital Universitário João de Barros Barreto: João Soares Felício, Alyne Maciel Lobato, Ana Regina Bastos Motta, Elquer Cardias Marques, Gabrielly Brito Balhieiro, Karem Miléo Felício, Melissa de Sá Oliveira dos Reis, Priscila Boaventura Barbosa de Figueiredo, Valéria Suênya Galvão Leal, Centro Financeiro, Ana Carolina Souza, Caroline Nunes Figueira , Franciane Melo, Gisely Mouta de Andrade Paes, Karem Mileo Felício, Natercia Neves Marques de Queiroz. (Enrolled participants=113)
4. Centro de Pesquisa Clínica do Coração/Hospital Cirurgia: Fábio Serra Silveira, Anisia Vieira Souza Fonte , Janaina Pinheiro Marinho da Silva, Maelyn Geoking de Oliveira Silveira, Tamyres Macenas de Oliveira Galvão, Maelyn Geoking de Oliveira Silveira, Maelyn Geoking de Oliveira Silveira, Dárcio Andrade de Melo, Marcos Serra Silveira. (Enrolled participants=88)
5. Centro de Pesquisas em Diabetes e Doenças Endocrino Metabólicas: Miguel Nasser Hissa, Giorlanda de Souza Pereira, Josiane Pontes, Lúcia Nasser Hissa, Maria Luiza Quindere Saraiva, Viviane Rocha Nasser Hissa, João Quinderé Saraiva, Fabiana Fiuza Alencar Araripe, Marcelo Rocha Nasser Hissa. (Enrolled participants=75)
6. Hospital Universitário São Francisco de Assis: Murillo de Oliveira Antunes, Smyrna Meuci Martins Marino, Joice Cristina Santos, Laura Dib Cassab, Larissa Maioli da Rosa, Mariana Raquel Gonçalves, Livia de Oliveira Antunes, Nicoli Papiani Gosmano, Pedro Henrique Breno de Andrade, Renata Nascimento Dias, Tibério Augusto Oliveira Costa. (Enrolled participants=75)
7. Centro de Pesquisas Clinicas Dr. Marco Mota (Centro Universitario Cesmac/ Hospital do Coração de Alagoas): Marco Antonio Mota Gomes, Saulo Ramalho, Annelise Gomes. (Enrolled participants=72)
8. i9 Pesquisa Clinica: Maria Helena Vidotti, Carolina Garcia Fass, Debora Virginia do Carmo Guimaraes, Janaina Keyla Cavalcante Carvalho, Liene Pagnan Rondi, Patrícia Dantas, Carina Forner. (Enrolled participants=31)
9. Hospital de Clinicas de Porto Alegre: Flávio Danni Fuchs, Andrei Rickrot, Jeruza Neyellof, Raquel Osório Luiz, Vera Lorentz de Oliveira Freitas, André Amon , Milene Sehn. (Enrolled participants=30)
10. CIPES - Centro Internacional de Pesquisa Clínica: Fabiana Goulart Marcondes Braga, Poliana Helena Rosolem Kishi, , Aristeu Haroldo Kimio Mizuta. (Enrolled participants=21)

11. H&W Cardiologia: Conrado Roberto Hoffman Filho, Drieli Meerholz, Leonardo Dyoran. (Enrolled participants=20)
12. Hospital Regional de Presidente Prudente: Charlene Troiani do Nascimento, Caroline Lucio Moreira Simões, Isabela Cristina Marocchio Vasconcellos , Marcelo Minszone, Mariana Gonçalves, Mariana Raquel Gonçalves , Luciane Schadeck Martins Portelinha, Michel Ullofo do Nascimento. (Enrolled participants=12)
13. Hospital Ruy Azeredo: Rafael Andrade de Azeredo Bastos, Emilly Giovannucci, Julia Guimarães, Katiuscia de Godoi Oliveira, Mariana Gonçalves, Jim Davis de Oliveira, José Maria Dias de Azeredo Bastos, Leandro Andrade de A. Bastos, Sara Borges de Oliveira. (Enrolled participants=12)
14. Hospital Universitario Pedro Ernesto - UERJ: Andrea Araújo Brandão, Ivy Machado, Simone Offrede, Erika Campana. (Enrolled participants=9)
15. Clinica Cardiologica: Elizabeth do Espirito Santo Cestario, Luana Beatriz de Souza Silva. (Enrolled participants=8)
16. Instituto do Coração do Hospital das Clínicas da FMUSP: Luiz Aparecido Bortolotto, Vivian Generoso Monteiro Moreira, Cassia C de Paiva. (Enrolled participants=2)
17. Hospital M Boi Mirim: Niklas Söderberg Campos, Edmilson Carvalho, Aline de Souza Ferreira, Maria das Vitórias Guedes, Niklas Söderberg Campos, Adriana Gomes Pereira Maurmo, Bruna Achar Söderberg Campos, Luiz Adriano Esteves, Paula Geraldes David Joao. (Enrolled participants=1)
18. Indacor Serviços Médicos LTDA-ME: Flávio de Souza Brito, Andrea Borges Calapodopulos Brito, Maria Eduarda Laguillo. (Enrolled participants=1)
19. Integral Pesquisa e Ensino: Mauro Esteves Hernandes, Hugo Morelli, Kamila Maria Silveira Negri, Silvio César Pantano, Gracielly de Souza Pantano. (Enrolled participants=1)

OPTION-TREAT Study Group

The following study group members were all closely involved with the design, implementation, and oversight of the OPTION-TREAT Trial:

Coordinating Center: Academic Research Organization (ARO) – Hospital Israelita Albert Einstein: Vagner Madrini Junior, Patrícia O. Guimarães, Caio A. M. Tavares, Monica T. A. Albuquerque, Mariana Castaldi Ramalho Silva, Flavia Ghizzoni, Ana Beatriz da Silva Correa Santos, Wilson José Milantoni, Francisco de Assis Queiroga Gonzaga Neto, Bruna do Santos Sampaio, Vitor Emanuel Freitas Mourão, Jaqueline Amalia de Paulo, Bruna Ladeira Moreno, Luciana Pereira Almeida de Piano, Maria Angela Amorim Dias, Roberta Momesso, Magali Satomi Ueda, Elaine de Jesus Santos, Cintia Selles Santos, Diogo Duarte Fagundes Moia, Lucas Ribeiro da Silva, Carlos Esteves, Glaucia Helena Castro de Freitas Novaes, Vitoria Santos de Oliveira Luz, Camila Santos Nascimento de Albuquerque, Adilio Roberto Bernardes, Tatiana Pinelli, Ronaldo Vicente Pereira Soares.

Data Safety Monitoring Board (DSMB): Luciano F. Drager (Chair), Felix J. A. Ramires, Tiago Mendonça.

Libbs Pharmaceuticals: Erida A. Pinto, Paula B. Fernandes, Maura G. Lapa, Augusto T. Figueiredo, Vivienne C. Castilho, Deborah Fucidji, Carolina O. R. Zilio.

Supplemental Tables

Supplemental Table S1. Inclusion and Exclusion Criteria

**Inclusion Criteria**
1. Age 18 years or older.
2. Current use of dual antihypertensive therapy with different therapeutic classes for at least 8 weeks and no response to treatment, defined as office measurements of SBP ≥140 mmHg and ≤180 mmHg and DBP ≥90 mmHg and ≤110 mmHg, assessed at screening and randomization visits.
3. Signed informed consent
**Exclusion Criteria**
1. Any clinical observation that the investigator interprets as a risk to the participant's involvement in the clinical study.
2. Any laboratory finding that the investigator considers a risk to the participant's involvement in the clinical study.
3. Suspected or diagnosed with coronavirus disease-19.
4. Known hypersensitivity to components of the medications used during the study or to sulfonamide-derived medications.
5. Pregnant or breastfeeding women.
6. Women of childbearing age who do not agree to use effective contraceptive methods, except those surgically sterile, postmenopausal for 1 year, or not engaging in sexual practices.
7. Male participants who do not agree to use effective contraceptive methods or whose partners do not agree to use effective contraceptive methods.
8. Participation in another clinical study in the last 12 months, unless direct benefit is foreseen by the investigator.
9. Second-degree or closer relationship or bond with sponsor's or research center's employees.
10. eGFR <45 ml/min/1.73m² or end-stage renal disease.
11. Severe liver dysfunction.
12. Cardiogenic shock or heart failure with reduced ejection fraction ≤50%.
13. Symptomatic congestive heart failure (NYHA classes II-IV) or recent myocardial infarction, unstable angina, or stroke within 6 months prior to study start.
14. Clinically significant ventricular cardiac arrhythmias.
15. Obstructive coronary artery disease scheduled for percutaneous or surgical coronary intervention.
16. Dementia,
17. Alcohol or illicit drug dependence in the prior 6 months
18. Use of prohibited medications.
19. Obstructive biliary disorders.
20. Refractory hypokalemia, hyperkalemia (serum potassium >5.5 mmol/L), or hyponatremia.
21. Symptomatic hyperuricemia
22. Secondary hypertension.
23. Malignant neoplasia without documentation of remission/cure

Abbreviations: SBP= systolic blood pressure; DBP= diastolic blood pressure; eGFR=estimated glomerular filtration rate; NYHA=New York Heart Association

Supplemental Table S2. Study Outcomes

**Primary Efficacy Outcome**
Mean change in SBP from baseline to 12 weeks
**Secondary Efficacy Outcomes**
Mean change in office DBP from baseline to 4, 8, and 12 weeks
Mean change in office SBP from baseline to 4 and 8 weeks
Proportion of participants who achieve SBP <140 and DBP <90 mmHg at 4, 8, and 12 weeks
Proportion of participants who achieve SBP <120 mmHg at 4, 8, and 12 weeks
Proportion of participants who achieve SBP <140 mmHg at 4, 8, and 12 weeks
Proportion of participants who achieve DBP <90 mmHg at 4, 8, and 12 weeks
Proportion of participants who achieve a reduction of 20 mmHg or more in SBP at 4, 8, and 12 weeks
Proportion of participants who achieve a reduction of 10 mmHg or more in DBP at 4, 8, and 12 weeks
**Safety Outcomes**
Incidence of adverse events from the first dose of the investigational product up to 30 days after the end of treatment
Proportion of participants with clinically relevant laboratory test alterations according to investigator's discretion at the screening visit and final visit
Proportion of participants with clinically relevant clinical or physical examination alterations according to investigator's discretion at the follow-up visits
Absolute change in vital signs from baseline to follow-up visits

Abbreviations: SBP= systolic blood pressure; DBP=diastolic blood pressure.

Supplemental Table S3. Post-hoc Sensitivity Analyses of the Primary Outcome

	Change in SBP from baseline to 12 weeks ± SE				
**Model**	Experimental Group (n=342)	Active Control Group (n=341)	Mean Difference (90% CI)	Mean Difference (95% CI)	p-value
Without baseline SBP as a covariate	–24.1 ± 0.83	–19.8 ± 0.83	–4.38 (–6.3 to –2.5)	–4.38 (–6.7 to –2.1)	<0.001[a]
Participants with ≥1 follow-up visit and baseline SBP as a covariate	–24.2 ± 0.91	–19.7 ± 0.91	–4.56 (–6.1 to –3.0)	–4.56 (–6.4 to –2.7)	<0.001[a]
Participants with ≥1 follow-up visit and without baseline SBP as a covariate	–24.2 ± 0.85	–19.7 ± 0.85	–4.82 (–6.6 to –3.0)	–4.82 (–7.0 to –2.7)	<0.001[a]

[a]The primary efficacy outcome was analyzed using a linear mixed-effects model for repeated measures. The noninferiority margin was set at 3 mmHg. P-value for noninferiority is presented. Blood pressure measurements are presented in mmHg.

Abbreviations: SBP = Systolic blood pressure; CI = Confidence interval; SE = Standard error

Supplemental Table S4. Proportion of Patients Achieving Blood Pressure Targets and Reductions at Weeks 4, 8, and 12

Blood Pressure	Visits	Experimental treatment (%)	Active Control (%)
SBP < 140 and DBP < 90 mmHg	Week 4	64.5	57.3
	Week 8	66.5	59.0
	Week 12	69.3	59.8
SBP < 120 mmHg	Week 4	26.4	14.0
	Week 8	25.0	17.1
	Week 12	28.1	16.5
SBP < 140 mmHg	Week 4	71.9	63.5
	Week 8	71.9	65.2
	Week 12	75.0	66.4
DBP < 90 mmHg	Week 4	76.7	75.5
	Week 8	79.0	74.4
	Week 12	81.5	74.6
SBP reduction ≥ 20 mmHg	Week 4	58.0	44.2
	Week 8	55.4	50.4
	Week 12	60.5	47.9
DBP reduction ≥ 10 mmHg	Week 4	68.5	63.2
	Week 8	67.9	64.4
	Week 12	70.5	66.4

Abbreviations: BP = Blood Pressure; SBP = Systolic Blood Pressure; DBP = Diastolic Blood Pressure; mmHg = Millimeters of Mercure

Supplemental Figures

Supplemental Figure S1. Study Design

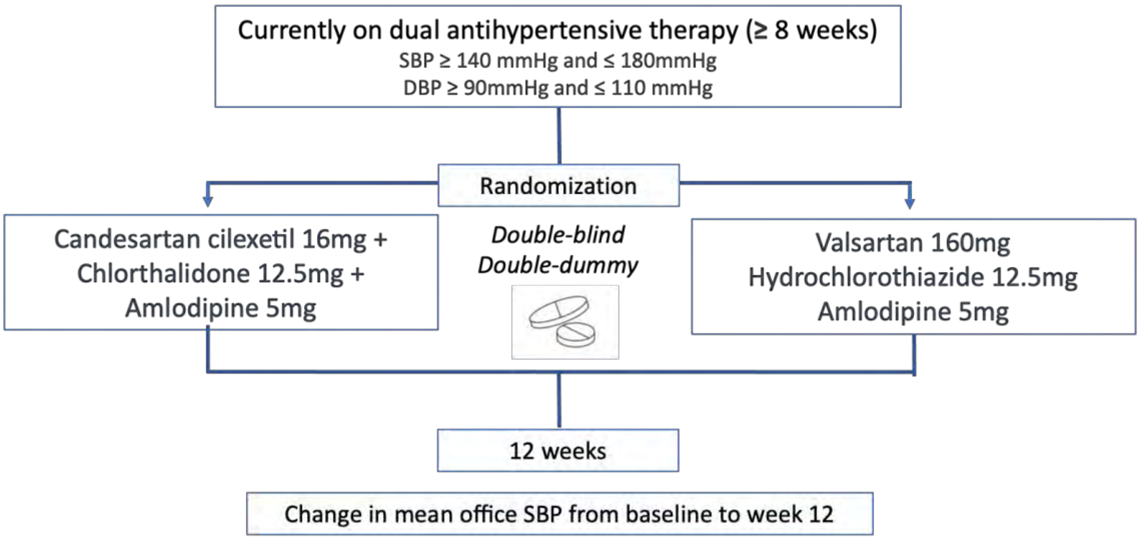

Abbreviations: SBP: Systolic Blood Pressure; **DBP**: Diastolic Blood Pressure

The OPTION TREAT Trial enrolled participants with uncontrolled hypertension, defined as office systolic blood pressure between 140 and 180 mmHg and diastolic blood pressure between 90 and 110 mmHg. Eligible participants were randomized to receive either a fixed-dose triple pill combination of candesartan cilexetil 16 mg, chlorthalidone 12.5 mg, and amlodipine 5 mg, or an active control triple pill consisting of valsartan 160 mg, hydrochlorothiazide 12.5 mg, and amlodipine 5 mg. The treatment period lasted 12 weeks. The primary endpoint was the change in mean office systolic blood pressure from baseline to week 12.

Supplemental Figure S2. Study Procedures

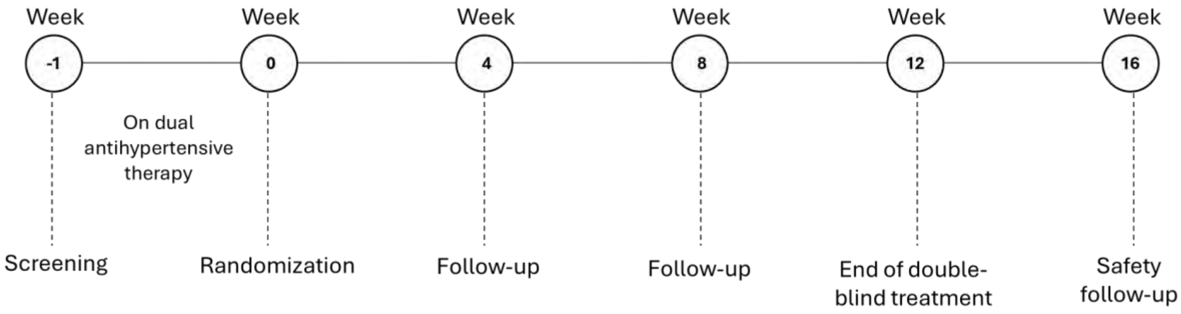

This timeline illustrates the schedule of study activities across the trial duration. The screening visit occurred at week –1, followed by randomization (week 0). Subsequent follow-up visits were conducted at weeks 4, 8, and 12, with a post-treatment safety follow-up visit at week 16. At each visit, key assessments included measurement of blood pressure, laboratory tests, safety evaluations, and assessment of adherence. These visits were designed to monitor efficacy and tolerability of the interventions over the 12-week treatment period, with an additional visit to ensure post-study safety.

Clinical Study Protocol

STUDY TITLE

National, phase III, multicenter, randomized, double-blind, controlled, parallel-group clinical study to evaluate the non-inferiority of the combination of candesartan cilexetil 16mg + chlorthalidone 12.5mg + amlodipine 5mg compared to Exforge HCT® (valsartan 160mg + hydrochlorothiazide 12.5mg + amlodipine 5mg) in the treatment of systemic arterial hypertension in adults of both sexes.

LB2009

**Sponsor:** Libbs Pharmaceuticals
**Version**: 4.0
**Document Date:** January 3, 2024

CONFIDENTIAL

This document is confidential to Libbs Pharmaceuticals. Acceptance of this document constitutes the recipient's agreement that no unpublished information contained herein shall be published or disclosed without prior written approval.

Table of contents

General Information 5

Study Overview 10

Study Schedule 14

Introduction 15

Experimental Medicinal Product 17

Candesartan Cilexetil 17
Pharmacokinetics 17
Pharmacodynamics 18
Adverse Events 18
Drug Interactions 18
Contraindications 19

Chlorthalidone 19
Pharmacokinetics 19
Adverse Events 20
Drug Interactions 20
Contraindications 21

Amlodipine Besylate 21
Pharmacokinetics 21
Pharmacodynamics 22
Adverse Events 22
Drug Interactions 22
Contraindications 23

Candesartan Cilexetil, Chlorthalidone And Amlodipine Besylate 23
Pharmacokinetics 23
Pharmacodynamics 23
Adverse Events 24
Drug Interactions 24
Contra Indications 27

Report Of Non-Clinical Trials 27

Acute Toxicity Study 27

Repeated Dose Toxicity And Reproductive Toxicity 28

Genotoxicity And Carcinogenicity 28

Studies Of Interest For The Evaluation Of Pharmacological Safety 28

Toxicokinetics Studies 29

Discussion And Conclusions On Non-Clinical Data 29

Clinical Trials Report 29

Literature Search Methodology 29

Description Of The Selected Studies 30

Rationale For The Experimental Drug 31

Justification For Conducting The Clinical Study 33

Risk And Benefits Assessment **34**
Drug Risks And Benefits Assessment 34
Study Risk-Benefit Assessment **36**
Objectives **37**
Primary Objective 37
Secondary Objectives 37
Eligibility Criteria For Study Population **37**
Study Population 37
Number Of Participants 37
Inclusion Criteria 38
Exclusion Criteria 38
Clinical Study **39**
Rationale For The Clinical Study 39
Duration Of Clinical Study 40
Clinical Study Design 40
Study Plan 41
Assessments **42**
Clinical And Physical Assessment 42
Clinical Assessment 43
Laboratory Tests 43
Adverse Event Assessment 44
Treatment Adherence Assessment 44
Procedures During Clinical Study Visits 45
Materials And Methods **47**
Treatment Group 47
Research Participant Identification 47
Study Medications 47
Description Of The Investigational Product 47
Comparator Product Description 47
Supply Of Study Medications 48
Packaging And Labeling 48
Storage 48
Receipt, Dispensing And Accounting Of Study Medications 49
Study Blinding 49
Unblinding Study Code 52
Medications / Treatments 52
Prohibited Medications/Treatments 53
Adverse Events **53**
Idefinition And Classification 53
Classifications And Evaluations Made By The Investigator Regarding The Adverse Event 54

Reporting Of Serious Adverse Events (Saes) 56
Following-Up Adverse Event Reports 56
Reports Of Direct And Indirect Pregnancy 57

Statistical Analysis Plan 57
Analysis Populations 59
Statistical Methods 59
Safety Analyses 59
Effectiveness Analyses 60
Primary Efficacy Analysis 60
Secondary Efficacy Analyses 60
Interim Analysis 61
Randomization And Allocation Concealment 61
Treatment Of Missing Or Invalid Data 61
Data Management 61

Ethical And Regulatory Considerations 62

Protocol/Amendments 62
Informed Consent Form (Icf) 63
Research Participant Confidentiality 63
Confidential Information And Publications 64
Care 64
Regulatory Aspects Of Clinical Research 64
Quality Control And Assurance 64
Source Documents 64
Clinical Study Monitoring 65
Audit 65
Independent Data Security Monitoring Committee 65

References 66

Annexes 72

Annex Iii – Package Leaflet 94

Annex Iv – Ldl Calculation (Low Density Lipoprotein) - Martin, 2013 95

GENERAL INFORMATION

**Protocol title:**<br>National, phase III, multicenter, randomized, double-blind, controlled, parallel clinical study to evaluate the non-inferiority of the combination candesartan cilexetil 16mg + chlorthalidone 12.5mg + amlodipine 5mg compared to Exforge HCT® (valsartan 160mg + hydrochlorothiazide 12.5mg + amlodipine 5mg) in the treatment of systemic arterial hypertension in adults of both sexes.		
**Identification code**<br>LB2009	**Date**<br>January 3, 2024	**Development phase**<br>Phase III
**Persons authorized by the sponsor to sign the protocol and amendments**<br>Vivienne Carduz Castilho<br>Medical Sciences Manager<br>Libbs Pharmaceuticals<br>vivienne.castilho@libbs.com.br		
**Study Biostatistician**<br>Maura Gonzaga Lapa<br>Specialist in Clinical Research Statistics<br>Libbs Pharmaceuticals<br>maura.lapa@libbs.com.br		

SIGNATURE PAGE

Study Title: National, phase III, multicenter, randomized, double-blind, controlled, parallel clinical study to evaluate the non-inferiority of the combination candesartan cilexetil 16mg + chlorthalidone 12.5mg + amlodipine 5mg compared to Exforge HCT® (valsartan 160mg + hydrochlorothiazide 12.5mg + amlodipine 5mg) in the treatment of systemic arterial hypertension in adults of both sexes.

My signature confirms that I have read and approved this protocol and assures that this clinical study will be conducted in accordance with all the requirements of this document, the Declaration of Helsinki, the Good Clinical Practice - Americas Document and all applicable regulatory requirements.

Signature of Sponsor Libbs Pharmaceuticals

__________________________________ Date:______/_____/______

Vivienne Carduz
Medical Sciences Manager
Libbs Pharmaceuticals

Signature of the ARO (Academic Research Organization) - Hospital Israelita Albert Einstein

__________________________________ Date:______/_____/______

Dr. Patrícia Oliveira
Guimarães
Cardiologist
Hospital Israelita Albert Einstein

Center:

Signature of Principal Investigator (Lead Researcher)

__________________________________ Date:______/_____/______

Dr. Vagner Madrini Junior
Principal Investigator (Lead Researcher)

LIST OF ABBREVIATIONS

ADL	Activities of Daily Living
NSAIDs	Non-steroidal anti-inflammatory drugs
AML	AMlodipine Besylate
ANVISA	Brazilian Health Regulatory Agency
AT1	Angiotensin II Receptor Subtype 1
ATC	Anatomical Therapeutic Chemical
CVA	Cerebrovascular Accident
BB	Beta-blockers
CCB	Calcium Channel Blockers
GCP	Good Clinical Practices
ARB	Angiotensin II Receptor Blockers
CAN	Candesartan Cilexetil
SN	Special Notice
EC	Ethics Committee
CIOMS	Council for International Organizations of Medical Sciences
CFR	Code of Federal Regulations
CNS	National Health Board
CONEP	National Research Ethics Committee
CRF	Case Report Form
CT	Telephone Contact
CV	Cardiovascular
CAD	Coronary Artery Disease
DCDD	Drug Clinical Development Dossier
SCTD	Specific Clinical Trial Dossier
DIU	Diuretics
AE	Adverse Events
TEAE	Treatment Emergent Adverse Events
SAEs	Serious Adverse Events

ACE	Angiotensin Converting Enzyme
RCT	Randomized Clinical Trials
eCRF	Electronic Case Report Form
ECG	Electrocardiogram
HR	Heart Rate
FDA	Food and Drug Administration
FEM	Fixed Effects Model
RR	Respiratory Rate
AH	Arterial Hypertension
AMI	Acute Myocardial Infarction
IBGE	*Instituto Brasileiro de Geografia e Estatística*
HF	Heart Failure
ICH	International Council for Harmonisation of Technical Requirements for Pharmaceuticals for Human Use
ICH-E9	Statistical Principles for Clinical Trials
RDI	Direct Renin Inhibitors
ACEI	Angiotensin Converting Enzyme Inhibitors
BMI	Body Mass Index
ITT	Intended-to-Treat Analysis Population
GDPR	General Data Protection Regulation
LOCF	Last Observation Carried Forward
MedDRA	Medical dictionary for regulatory activities
MMRM	Mixed Model for Repeated Measures
MS	Ministry of Health
msDBP	Mean Standard Diastolic Blood Pressure
msSBP	Mean Standard Systolic Blood Pressure
NI	Notification to Investigators
WHO	World Health Organization
BP	Blood Pressure
DBP	Diastolic Blood Pressure
SBP	Systolic Blood Pressure
SOP	Standard Operating Procedure
PP	Per-Protocol (PP) Population

PT	Preferred Term (MedDra Dictionary)
IP	Investigational Product (experimental medication)
CBR	Collegiate Board Resolution
OSC	Organ System Class (MedDra Dictionary)
RAS	Renin-Angiotensin System
SADR	Suspected Adverse Drug Reaction
SUS	Brazilian Unified Health System
ICF	Informed Consent Form
V0	Randomization Visit
V-1	Screening Visit
V1	Follow-up Visit
V2	Follow-up Visit
DV	Direct Vasodilators
UV	Unescheduled Visit
FV	Final Visit
WHO-UMC	World Health Organization - Upssala Monitoring Centre

Libbs

STUDY OVERVIEW

Clinical Study Code	LB2009
Clinical Study Title	National, phase III, multicenter, randomized, double-blind, controlled, parallel clinical study to evaluate the non-inferiority of the combination of candesartan cilexetil 16mg + chlorthalidone 12.5mg + amlodipine 5mg compared to Exforge HCT® (valsartan 160mg + hydrochlorothiazide 12.5mg + amlodipine 5mg) in the treatment of systemic arterial hypertension in adults of both sexes
Version	Version 4.0
Document Date	January 3, 2024
Clinical Study Population	Participants of both sexes, aged 18 years or older, who, despite currently using dual antihypertensive therapy from different therapeutic classes for at least 8 weeks, have maintained SBP measurements ≥ 140 mmHg and ≤180 mmHg and DBP ≥ 90 mmHg and ≤ 110 mmHg.
Primary Objective	To evaluate the efficacy through non-inferiority of the combination of candesartan cilexetil 16mg + chlorthalidone 12.5mg + amlodipine 5mg compared to Exforge HCT® (valsartan 160mg + hydrochlorothiazide 12.5mg + amlodipine 5mg) in the mean change in systolic blood pressure 12 weeks after initiation of treatment compared to baseline.
Primary efficacy endpoint	The primary efficacy endpoint is the mean change in SBP, measured at the research center, 12 weeks (± 4 days) after the initiation of treatment, compared to baseline (V0).
Secondary Objectives	• To evaluate the change in DBP 4, 8 and 12 weeks after the initiation of treatment;<br>• To evaluate the change in SBP 4 and 8 weeks after the initiation of treatment;<br>• To evaluate the proportion of participants achieving the target blood pressure of SBP <140 and DBP <90 mmHg 4, 8 and 12 weeks after the initiation of treatment;<br>• To evaluate the proportion of participants achieving the target blood pressure of SBP <120 mmHg 4, 8 and 12 weeks after the initiation of treatment;<br>• To evaluate the proportion of participants achieving the target SBP of < 140 mmHg 4, 8 and 12 weeks after the initiation of treatment;<br>• To evaluate the proportion of participants achieving the target DBP of < 90 mmHg 4, 8 and 12 weeks after the initiation of treatment;<br>• To evaluate the proportion of participants who achieve a reduction of greater than or equal to 20 mmHg in SBP 4, 8 and 12 weeks after the initiation of treatment;<br>• To evaluate the proportion of participants who achieve a reduction greater than or equal to 10 mmHg in DBP 4, 8 and 12 weeks after the initiation of treatment.

Secondary Efficacy Endpoints	• Mean change in DBP, measured at the research center at 4, 8 and 12 weeks (± 4 days) after the initiation of treatment, compared to baseline (V0);<br>• Mean change in SBP, measured at the research center at 4 and 8 weeks (± 4 days) after the initiation of treatment, compared to baseline (V0);
Safety Objectives	To evaluate the safety of treatments based on the occurrence of adverse events and changes in laboratory parameters, clinical/physical evaluation and vital signs.
Safety Endpoints	• Incidence of adverse events (AEs) recorded from the first dose of IP up to 30 days after the end of the treatment defined in the protocol; however, all adverse events occurring after informed consent is obtained must also be collected and reported;<br>• Incidence of any AE occurring after informed consent has been obtained up to 30 days after the end of the treatment period defined in the protocol;<br>• Proportion of participants with laboratory abnormalities considered to be clinically relevant determined by the investigator, at the screening visit (V-1) and final visit (FV) of the study;<br>• Proportion of participants with clinical or physical abnormalities, considered to be clinically relevant determined by the investigator, at visits V1, V2 and FV compared to baseline (V0);<br>• Absolute change in vital signs measured at visits V1, V2 and FV compared to the baseline visit (V0); Note: Although only adverse events occurring after the use of the experimental/comparator drug will be used in the analysis that will evaluate the safety of the treatments, all adverse events occurring after the consent obtained through the ICF will be collected and reported.
Methodology	The study includes a Screening Visit (V-1), a Randomization Visit (V0), two Follow-up Visits (V1 and V2), a Final Visit (FV) and a telephone contact (CT).<br><br>The Screening visit (V-1) will be the first visit of the study and all procedures must be conducted only after informed consent is obtained through the ICF. The purpose of this visit is to assess the eligibility criteria that may be evaluated at this stage of the study.<br><br>The Randomization Visit (V0) will take place one week (+4 days) after the Screening Visit (V-1). At this visit, all eligibility criteria must be verified and, if the participant is eligible, he/she must be randomized and assigned to one of two treatment groups:<br><br>Group 1: Candesartan cilexetil 16mg + chlorthalidone 12.5mg + amlodipine 5mg;<br><br>Group 2: Exforge HCT® (valsartan 160mg + hydrochlorothiazide 12.5mg + amlodipine 5mg)

	The Follow-up Visits (V1 and V2) will take place four and eight weeks (± 4 days) after the date of the Randomization Visit (V0). The purpose of this visit is to evaluate the efficacy and safety of the IP during the course of the study.<br><br>The Final Visit (FV) will take place twelve weeks (± 4 days) after the date of the Randomization Visit (V0). This visit aims to evaluate the effectiveness and safety of IP after the end of treatment. This visit will be the final in-person visit of the study, therefore, the research participant must be informed of the completion of the study.<br><br>Telephone Contact (CT) will be conducted 30 (+7) days after the end of treatment with IP. The purpose of this contact is to assess the possible occurrence of adverse events during this period.
Inclusion Criteria	• Participants of both sexes aged 18 years or older;<br>• Participants currently using dual anti-hypertensive therapy from different therapeutic classes for at least 8 weeks and non-responders to this treatment, defined as presenting with office measurements of SBP ≥ 140 mmHg and ≤180 mmHg and DBP ≥ 90 mmHg and ≤ 110 mmHg, assessed at both screening and randomization visit (both conditions are in accordance with the Brazilian Hypertension Guideline - – 2020);<br>• Ability to understand and consent to their participation in this clinical study, documented by signing the Informed Consent Form (ICF).
Exclusion Criteria	• Any clinical finding (from clinical/physical evaluation and vital signs assessments) that, in the opinion of the investigator, represent a risk to the research participant's participation in the clinical study;<br>• Any laboratory test finding that the investigator considers to represent a risk to the research participant regarding their participation in the clinical study;<br>• Participants suspected or diagnosed with COVID 19; Known hypersensitivity to any component of the study medication or to agents derived from sulphonamides;<br>• Women who are pregnant or breastfeeding;<br>• Menopausal women who do not agree to use effective contraceptive methods [oral contraceptive, injectable contraceptive, intrauterine device, hormonal implant, barrier methods, hormonal transdermal patch, vaginal ring and tubal ligation]; except those who are surgically sterile (bilateral oophorectomy or hysterectomy), those who have been menopausal for at least one (1) year and participants who declare that they are sexual abstinent or engage exclusively in non-reproductive sexual practices;<br>• Male participants who do not agree to use effective contraceptive methods:<br>contraceptive methods for the participant:

	condom, except for those who are surgically sterile (vasectomy) or for participants who are sexually abstinent during the study period or participants who declare that they are sexual abstinent or engage exclusively in non-reproductive sexual practices; OR<br>contraceptive methods for the female partner: oral contraceptive, injectable contraceptive, intrauterine device, hormonal implant, hormonal transdermal patch, tubal ligation, vaginal ring and barrier methods except for female partners who are surgically sterile (bilateral oophorectomy or hysterectomy), menopausal for at least one (1) year;<br>• Participants with a history of obstructive coronary artery disease scheduled for percutaneous or surgical coronary intervention;<br>• Participants with a history of dementia syndrome;<br>• History of alcohol or illicit drug dependence within the six months prior to the date of informed consent;<br>• Participants who use prohibited concomitant medication as described in item 9.1;<br>• Participants with a history of obstructive biliary disorders;<br>• Participants with a history of refractory hypokalemia and/or conditions involving marked loss of potassium, hyperkalemia (with serum potassium levels above 5.5 mmol/L) and/or hyponatremia;<br>• Participants with a history of symptomatic hyperuricemia (history of gout or uric acid nephrolithiasis);<br>• Participants with a history of secondary arterial hypertension;<br>• Participants with a medical history of malignant neoplasia, without documentation of remission/cure.
Investigational Product	Candesartan cilexetil 16 mg + chlorthalidone 12.5 mg + amlodipine 5 mg;<br>Pharmaceutical form: tablet;<br>- Dosing: administer one (1) tablet of the active investigational drug and then one (1) tablet of comparator placebo, orally, once daily in the morning.
Comparator Product	- Exforge HCT® (valsartan 160 mg + hydrochlorothiazide 12.5 mg + amlodipine 5 mg);<br>- Pharmaceutical form: coated tablet;<br>- Dosing: administer one (1) tablet of the active comparator and then one (1) placebo tablet of the investigational product, orally, once daily in the morning.
Number of Research Participants	698 randomized research participants.
Treatment Period	Twelve weeks (± 4 days).
Duration of Participation in the Study	Seventeen weeks (± 4 days), considering the period from the Screening Visit (V-1) up to the Telephone Contact (TC) conducted 30 days after the end of treatment (+7).

STUDY SCHEDULE

Visit	V-1	V0	V1	V2	FV	TC
Week (days)	- 1 (+ 4)	0	4 (± 4)	8 (± 4)	12 (± 4)	30 days after end of treatment (+7)
Study phase	Screening	Randomization	Follow-up	Follow-up	Final	Follow-up
**Selection**						
Informed consent	x					
Eligibility criteria assessment	x	x				
Randomization		x				
**Treatment**						
Previous/Concomitant/Prohibited Medications and Treatments Assessment	x	x	x	x	x	
IP Dispension		x	x	x		
IP return and checking / accounting			x	x	x	
Treatment adherence assessment			x	x	x	
**Efficacy and safety**						
Clinical / Physical Assessment / Vital signs	x	x	x	x	x	
Collection of laboratory testing[1]	x		x	x	x	
Male condoms dispension[2]	x	x	x	x	x	
Adverse Events Assessment	x	x	x	x	x	x
Discontinuation Criteria		x	x	x		

1 Laboratory tests should be evaluated as soon as the results are available; in the event of abnormalities, at the discretion of the investigator, the participant may be summoned to return to the research site for evaluation;

2 Male condoms will be offered as a barrier method of contraception. Participants who use other effective contraception methods will be instructed to continue with their current method. Participants who declare that are sexual abstinent or only engage in non-reproductive practices may refuse to receive condoms.

Note: The Clinical Trial will only begin after it is approved by the Ethics Committees (CEP/CONEP) and the regulatory authorities (ANVISA).

INTRODUCTION

According to the 2020 Brazilian Hypertension Guidelines, arterial hypertension (AH) is defined as a chronic, non-communicable, multifactorial disease that depends on genetic, environmental and social factors. It is diagnosed by sustained elevation of systolic blood pressure (SBP) greater than or equal to 140 mmHg and/or diastolic BP (DBP) greater than or equal to 90 mmHg measured on at least two different occasions in the absence of antihypertensive medication (BARROSO, 2020; WILLIAMS, 2018; FOROUZANFAR, 2017; ZHOU, 2021).

According to data from the World Health Organization, 1.13 billion people worldwide are diagnosed with AH, equivalent to 1 in 4 men and 1 in 5 women (WORLD HEALTH ORGANIZATION, 2015). In Brazil, its prevalence is 22.8% of the population, 20.0% in women, 25.8% in men and 47.0% among individuals aged 75 years or older (MALTA, 2016; PICON, 2012). Regarding cardiovascular complications, AH is the most frequent cause of morbidity and the main independent risk factor for other complications such as stroke, acute myocardial infarction (AMI), chronic kidney disease, among others (MALTA, 2016). By 2018, cardiovascular diseases accounted for 28% of all deaths in the country. Worldwide, the trends are similar (WORLD HEALTH ORGANIZATION, 2018).

In addition to the impairment in quality of life and life expectancy and the impact on the lives of relatives, friends and work colleagues, AH entails high economic costs. In 2018, the Brazilian Unified Health System (SUS) spent approximately 2 billion reais on hospital admissions, outpatient care and medications, directly affecting the health system and the economy (MALTA, 2016; NILSON; 2018). The main goal of AH treatment is to achieve blood pressure control, reaching the previously established levels and thus reducing mortality and major cardiovascular event. To achieve these goals, pharmacological treatment is recommended (BARROSO, 2020; NATIONAL HIGH BLOOD PRESSURE EDUCATION PROGRAM WILLIAMS, 2018).

Regarding the pharmacological treatment of AH, in more than two thirds of cases, it is necessary to use 2 or more agents from different classes, even 3 or more in patients with very high levels of blood pressure or with the need to achieve lower pressure levels (BARROSO, 2020; NATIONAL HIGH BLOOD PRESSURE EDUCATION PROGRAM, 2004; WILLIAMS, 2018). The six main drugs classes used for AH are: diuretics, which can be thiazide, loop or potassium-sparing diuretics; calcium channel blockers (CCBs), dihydropyridines and non-hydropyridines; angiotensin-converting enzyme inhibitors (ACEIs); angiotensin II receptor blockers (ARB); beta-blockers (BB), including those with intrinsic sympathomimetic activity and a combination of alpha and beta adrenergic blockers; and direct vasodilators (DV) (LAW, 2009; MCLEAN, 2016; BARROSO, 2020).

The treatment choice for AH is individualized, based on individualized characteristics such as age, cardiovascular complications, concomitant diseases, target organ damage, blood pressure and socioeconomic condition (CAREY, 2018; WILLIAMS, 2018; MALACHIAS, 2016; OLIVEIRA, 2017). Particularly, in case of monotherapy for AH (with the use of only one drug), patients should be considered to be in stage 1 of HA (140-159/90-99 mm/Hg) with a low risk of cardiovascular complications or pre-hypertension (130- 139/85-89 mm/Hg) with a high risk of cardiovascular complications (FLYNN, 2017); using drugs from the IUD, BCC, ACEI and ARB classes (BARROSO, 2020).

However, monotherapy has often not been sufficient for controlling blood pressure, leading to an increased risk of developing major cardiovascular events, a higher incidence of adverse events related to high doses of medications and, in cases of constant switching of drug classes, less commitment and patient confidence treatment (GRADMAN, 2010; VOLPE, 2020). In combination therapy, currently considered the preferred therapeutic strategy regardless of the stage of AH and associated cardiovascular risk, treatment is conducted with drugs with different mechanisms of action. The rationale is based on increasing the antihypertensive effect of these drugs by acting through different pathophysiological mechanisms, a synergistic effect, inhibition of the activation of counter-regulatory mechanisms and the use of lower doses/ability of one drug to antagonize the adverse effects of the other, resulting in a lower incidence of these effects (BARROSO, 2020; JAMERSON, 2004; VOLPE, 2020).

Figure 1 shows the flowchart of the combination therapy rationale for the treatment of hypertension, with the addition of new drugs with different mechanisms when BP is not controlled, continuing until the desired goal is achieved (BARROSO, 2020). Figure 2 shows the preferred combinations (connected by a green line), the contraindicated combinations (in dashed red) and the possible but less studied combinations (dotted line (MALACHIAS, 2016).

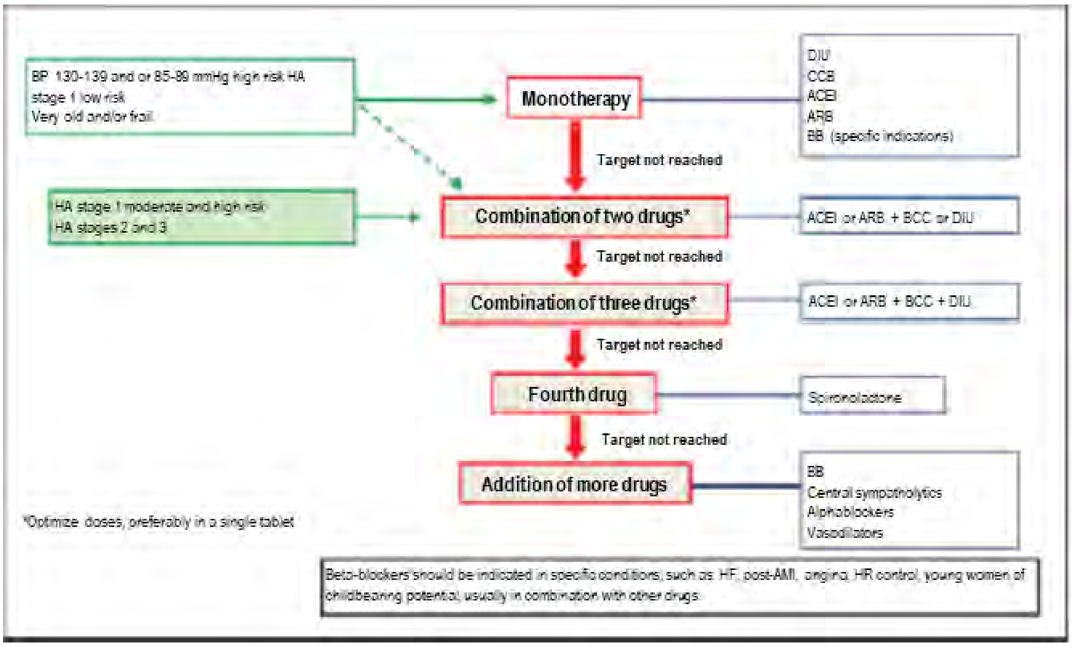

Figure 1 - Flowchart of drug treatment for arterial hypertension (AH). BP = blood pressure; IUD = diuretic; BBC = calcium channel blocker; ACEI = angiotensin-converting enzyme inhibitor; ARB = angiotensin receptor blocker; BB = beta-blocker; HR = heart rate.

Source: Adapted from BARROSO, 2020.

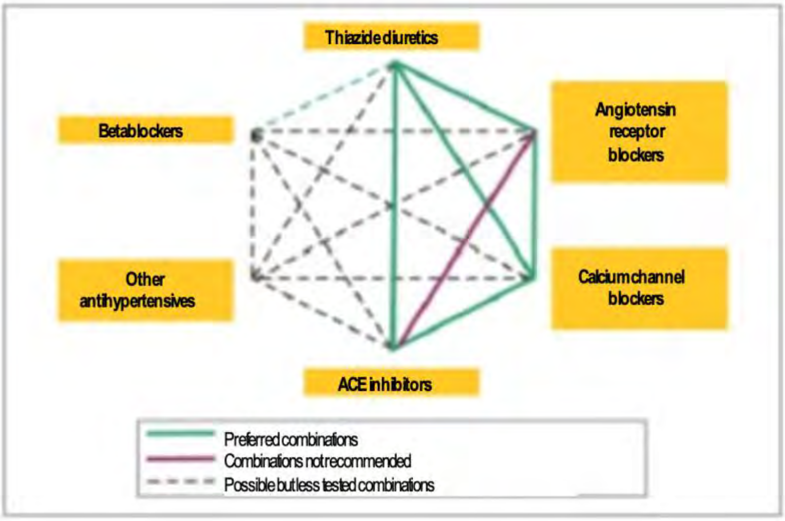

Figure 2 - Preferred drug combination scheme based onto mechanisms of action and synergy.

Source: Adapted from MALACHIAS, 2016.

Based on this rationale and greater convenience for the patient, leading to increased adherence to treatment, current guidelines encourage the use of pharmaceutical forms associating two or more anti-hypertensive drugs (WILLIAMS, 2018; VOLPE, 2020; WEBSTER, 2018; SALAM, 2014).

In particular, the combination of a calcium channel blocker, inhibiting the transmembrane influx of calcium ions in vascular smooth muscle and cardiac muscle, with an angiotensin receptor blocker, inhibiting angiotensin II-mediated vasoconstriction and renal sodium retention, and a thiazide diuretic, reducing intravascular volume and total body sodium, (association BCC+ARB+DIU) presents a synergistic effect of ARB+DIU and BCC+ARB providing a superior antihypertensive effect in combination when compared to the agents alone (CALHOUN, 2009; ALLEMANN, 2008; LEE, 2020; BRAMLAGE, 2018).

Based on the information presented and aiming to achieve a synergistic effect of the combination of agents in the treatment of hypertension, greater control of blood pressure levels and patient adherence, the proposal is to study a triple combination consisting of amlodipine (calcium channel blocker) at a dosage of 5 mg, candesartan cilexetil (angiotensin receptor blocker) at 16 mg and the diuretic chlorthalidone at 12.5 mg, in a single fixed-dose formulation containing the three anti-hypertensive drugs.

EXPERIMENTAL MEDICINAL PRODUCT

Candesartan cilexetil

A prodrug formed by the esterification of candesartan, the active form. Non-peptide antagonist of the angiotensin II (AT1) type 1 receptor (GLEITER, 2002).

Pharmacokinetics

Physical-Chemical Properties
Chemical name:
Candesartana cilexetila (prodrug): 1-cyclohexyloxycarbonyloxyethyl 2-ethoxy-3-[[4-[2-[2-(2H-tetrazol-5-yl)phenyl]phenyl]methyl]benzimidazole-4-carboxylate (PUBCHEM_candesartana cilexetila_2540).
Candesartana (active drug): 2-ethoxy-3-[[4-[4-[2-(2-(2*H*-tetrazol-5-il)phenyl]phenyl]phenyl]methyl]benzimidazole-4-carboxylic acid (PUBCHEM_candesartana_2541).

Molecular formula:
Candesartan cilexetil: C33H34N6O6 (PUBCHEM_candesartan cilexetil_2540).
Candesartan: C24H20N6O3 (PUBCHEM_candesartan_2541).

Chemical structure:

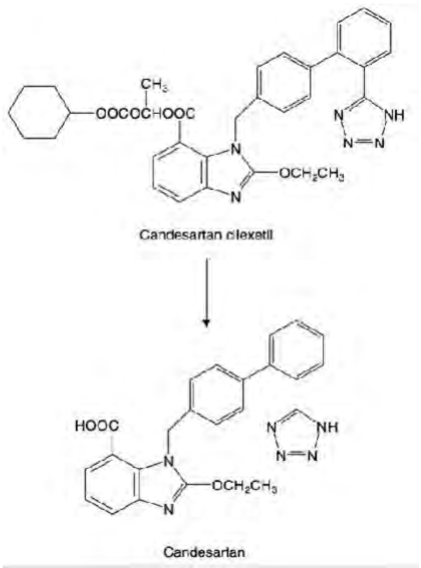

Figure 3: Chemical structure of candesartan and candesartan cilexetil
Source: Extracted from GLEITER 2002.

Pharmacodynamics

Mechanism of action

Angiotensin II regulates blood pressure as well aswater and electrolyte balance by acting on the AT1 receptor. Its interaction with the AT1 receptor causes vasoconstriction and stimulation of aldosterone and vasopressin secretion, resulting in sodium reabsorption in the renal tubule, increased peripheral noradrenergic and central sympathetic activity and reduced renal blood flow (JOHNSTON, 1997).
Candesartan is a non-peptide angiotensin II receptor blocker which selectively binds to and slowly dissociates from the angiotensin II subtype 1 (AT1) receptor, thereby inhibiting the activity of angiotensin II in the receptor. It has no activity on the subtype 2 receptor (AT2) (MCCLELLAN, 1998).

Dosage
1.2.1.2.2.1 Dosage for adults
The clinically recommended effective dose of candesartan cilexetil for the treatment of hypertension is 4 to 32 mg once a day (MCCLELLAN, 1998; GAVRAS, 2000).
For adults and the elderly, the starting dose is 16 mg a day. In cases of intravascular volume depletion, the initial dose should be reduced to 8 mg a day. The usual dose ranges from 8 to 32 mg a day (BULSARA, 2021).

Dosage for pregnant women
Pregnancy risk category D. The use of candesartan during the second or third trimester of pregnancy is associated with oligohydramnios, fetal growth retardation, pulmonary hypoplasia, limb contracture, skullcap hypoplasia, neonatal death (stillbirth) and births with kidney damage. Use in the first trimester of pregnancy is associated with impaired fetal vascular perfusion and kidney function (ALWAN, 2005).

Dosage for patients with renal insufficiency
A dosage of 8 mg a day of candesartan cilexetil is suitable for patients with mild to moderate renal insufficiency without the need for a dose adjustment. Starting treatment with lower doses, 4 mg a day, may be appropriate in patients with severe kidney failure, including patients undergoing hemodialysis (DE ZEEUW, 1997; MCCLELLAN, 1998).

Adverse Events
The most common adverse events are symptomatic hypotension, kidney function impairement (increased creatinine), and hyperkalemia, with incidences of 18.8%, 12.5%, and 6.3%, respectively. Hypotension is more common in patients with volume or salt depletion secondary to dietary restriction, patients on dialysis, with diarrhea, emesis, or taking diuretics (LUND, 2018). Other reported adverse effects include headache, back pain, angioedema, and symptoms of upper respiratory tract infection, dizziness, syncope, muscle weakness, hypotension, skin rash, skin inflammation, urticaria, pruritus, alopecia, dry skin, diarrhea, abdominal pain, nausea, constipation, dry mouth and toothache (GAVRAS, 2000; HUSAIN, 2011; LUND, 2018).

Drug interactions
When used with diuretics, candesartan can lead to significant hypotension. The diuretics that interact with candesartan are furosemide, hydrochlorothiazide, potassium-sparing drugs such as spironolactone, triamterene and amiloride. The use of non-steroidal anti-inflammatory drugs (NSAIDs), especially in patients with heart failure, can lead to increased blood pressure and edema. Elderly patients with kidney insufficiency, kidney failure, who use diuretics or who are dehydrated, the use of NSAIDs concomitantly with candesartan NSAIDs concomitantly with candesartan can lead to kidney failure. The use of potassium-containing supplements can lead to hyperkalemia. The use of lithium with candesartan can lead to an increase in serum lithium concentration and toxicity (HUSAIN, 2011). There is no clinical evidence demonstrating the effects of food on candesartan cilexetil therapy (GLEITER, 2002). According to the Micromedex database, candesartan cilexetil may promote the following drug interactions (MICROMEDEX DRUGDEX CANDESARTANA CILEXETILA): Increased risk of adverse events (i.e. hypotension, syncope, hyperkalemia, changes in kidney function, acute kidney failure): benazepril, captopril, enalapril, enalaprilat, fosinopril, lisinopril, moexipril, perindopril, quinapril, ramipril and trandolapril; Kidney impairment and/or increased blood pressure: bronfenac, bufexamac, celecoxib, choline salicylate, clonixin, dexibuprofen, dexketoprofen, diclofenac, diflunisal, dipyrone, droxicam, etodolac, etofenamate, etoricoxib, felbinac, fenoprofen, fepradinol, feprazone, floctafenine, flufenamic acid, flurbiprofen, ibuprofen, indomethacin, ketoprofen, ketorolac, lornoxicam, loxoprofen, lumiracoxib, meclofenamate, mefenamic acid, meloxicam, morniflumate, nabumetone, naproxen, nepafenac, niflumic acid, nimesulide, nimesulide beta cyclodextrin, oxaprozin, oxyphenbutazone, parecoxib, phenylbutazone, piketoprofen, piroxicam, proglumetacin, propionic acid, propifenazone, proquazone, rofecoxib, salicylic acid, salsalate, sodium salicylate, sulindac, tenoxicam, tiaprofenic acid, tolfenamic acid, tolmetin and valdecoxib; Increased risk of hypoglycemia: insulin; Increased risk of lithium toxicity: lithium; Reduced

efficacy of angiotensin II receptor antagonists: Ma Huang and Ioimbine; Increased risk of hyperkalemia: Trimethoprim.

Contraindications

The use of candesartan cilexetil is contraindicated in cases of hypersensitivity to the drug or to any of the components of the formula (LUND, 2018).The use of angiotensin II AT1 receptor blockers in pregnant women can cause damage to fetal development and even neonatal death. Therefore, candesartan cilexetil should be discontinued as soon as possible in pregnant women (HUSAIN, 2011).The use of candesartan in patients with mild to moderate kidney insufficiency should not exceed a daily dose of 8 mg, while in patients with severe kidney insufficiency, the daily dose should not exceed 4 mg (DE ZEEUW, 1997; MCCLELLAN, 1998).According to the Micromedex database, candesartan cilexetil is contraindicated for concomitant use with aliskiren in diabetic patients; or in those with hypersensitivity to candesartan (MICROMEDEX_DRUGDEX_CANDESARTANA CILEXETILA).

Chlorthalidone

Chlorthalidone is a sulfonamide-derived thiazide diuretic approved for the treatment of hypertension, edema and congestive heart failure (KERNDT, 2021).

Pharmacokinetics

Physicochemical properties
Chemical name: 2-chloro-5-(1-hydroxy-3-oxo-2H-isoindol-1-yl)benzenesulfonamide (PUBCHEM)

Molecular formula: C14H11CIN2O4S (PUBCHEM_clortalidona_2732).
Chemical structure:

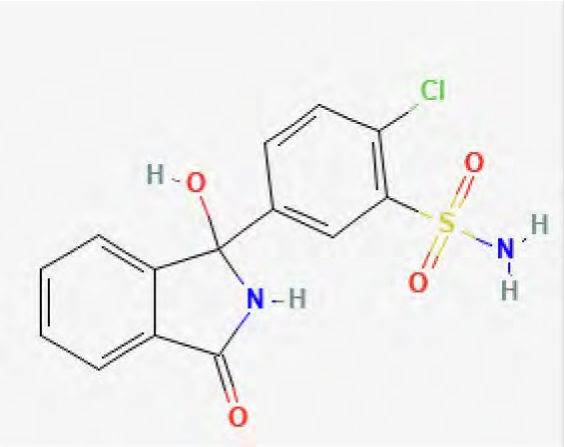

Figure 4: Chemical structure of chlorthalidone
Source: Extracted from PUBCHEM_chlortalidone_2732.

Mechanism of action:

Chlorthalidone is an inhibitor of the sodium-chloride co-transporter in the distal convoluted tubule of the nephron, reducing the reabsorption of sodium and chloride. By inhibiting sodium reabsorption, the distal convoluted tubule of the nephron retains a high concentration of sodium. The lack of sodium reabsorption alters the osmotic gradient and changes the distribution of fluid from the outside to the inside of the tubule. The increase in osmotic load due to the increase in sodium concentration leads to an increase in intratubular volume, thus promoting the diuretic effect. The increased excretion of sodium and extracellular fluids decreases the concentration of water and intravascular solutes. By reducing the intravascular volume and osmotic gradient, the patient

have a reduction in hydrostatic pressure, allowing blood pressure to be reduced (KERNDT, 2021).

Dosage

Dosage for adults: For patients with heart failure, the initial dose used is 12.5 mg or 25 mg a day, the maximum dose is 100 mg a day. For patients with generalized edema, the starting dose should be 50 mg or 100 mg a day, the maximum dose is 200 mg a day. For patients with nephrolithiasis, the recommended dose is 25 mg a day (KERNDT, 2021).
The age of patients should be an important factor to when determining the daily dose of chlorthalidone. Elderly patients aged 65 years or over should take lower doses, starting at 6.25 mg up to 12.5 mg a day, increasing gradually if necessary (KERNDT, 2021).

Dosage for pregnant women: Pregnancy risk category B. Chlorthalidone crosses the placental barrier, but does not alter the volume of amniotic fluid. It may cause hyperuricemia, hypokalemia, hyponatremia, hypomagnesemia, hypocalcemia and maternal hyperglycemia, as well as neonatal thrombocytopenia. Chlorthalidone is excreted in breast milk and can reduce milk production (OLIVEIRA, 2009).

Dosage for patients with renal insufficiency: Doses of 12.5 mg, 25 mg and 50 mg a day are safe for patients with chronic kidney disease (AGARWAL, 2014).

Adverse Events

The most significant adverse events are hypokalemia, hyponatremia, hypersensitivity reaction, and precipitation of acute gout attacks (KERNDT, 2021). Other adverse events can also be reported: Gastrointestinal effects: anorexia, gastric irritation, nausea, emesis, colic, loose stools, constipation and pancreatitis. Neurological reactions: paresthesia, dizziness and headache. Hematological reactions: aplastic anemia, leukopenia, agranulocytosis and thrombocytopenia. Cardiovascular reactions: orthostatic hypotension. Dermatological reactions: purpura, photosensitivity, skin rash, urticaria, cutaneous vasculitis and Lyell's syndrome (toxic epidermal necrolysis). Other reactions: hyperglycemia, glycosuria, hyperuricemia, muscle spasm, weakness, restlessness and impotence.

Drug interactions

Due to the decrease in serum potassium concentrations, chlorthalidone may increase digitalis toxicity and the action of neuromuscular competitive blockers with muscle relaxant action such as tuborarin or triiodide galamine. The decrease in potassium can be intensified by concomitant use with corticosteroids, corticotrophins, carbenoxolone and amphotericin B. Cardiac dysrhythmias (torsades de pointes) can be caused by a decrease in serum potassium. Chlorthalidone can also increase the effect of antihypertensive drugs such as guanethidine sulphate, methyldopa or ganglionic blocking drugs. Postural hypotension due to therapy with thiazide diuretics can be increased by concomitant intake with alcohol, barbiturates or opioids.

Concomitant administration of chlorthalidone with lithium is not recommended, as lithium concentrations in the blood may increase. The pharmacological effects of hypoglycemic drugs can be reduced by chlorthalidone. Non-steroidal anti-inflammatory drugs can antagonize the actions of chlorthalidone. The hyperglycemic, hypotensive and hyperuricemic effects of diazoxides may be potentiated by chlorthalidone.

The concomitant use of chlorthalidone with probenecid increases the excretion of calcium, magnesium and citrate, but does not affect the excretion of sodium, potassium, ammonium chloride, bicarbonate and phosphate. Chlorthalidone increases urinary pH and can decrease the excretion of amphetamines and quinidines, and decrease the effectiveness of methenamine due to alkalinization of the urine. The effects of oral anticoagulants may be reduced when used simultaneously with chlorthalidone.

Pre-anesthetic drugs and anesthetics used in surgery mayhave their effects enhanced when used simultaneously with chlorthalidone. Chlorthalidone and acetazolamine compete for the same binding site on erythrocytes. The information described in this section was taken from PUBCHEM_chlortalidone_2732 (2021) and the INCHEM database (1992). According to the Micromedex database, chlorthalidone can promote the following drug interactions (MICROMEDEX_DRUGDEX_CLORTALIDONE): Increased risk of hyperglycemia; increased need for insulin, acarbose, albiglutide, alogliptin, bromocriptine, canagliflozin, chlorpropamide,dapagliflozin, dulaglutide, empagliflozin, exenatide, glimepiride, glipizide, gliburide, insulins, linagliptin, liraglutide, lixisenatide, miglitol, nateglinide, pioglitazone, pramlintide, repaglinide, rosiglitazone, saxagliptin, sitagliptin, tolazamide, tolbutamide and vildagliptin; Reduced diuretic efficacy and possible nephrotoxicity: aceclofenac, acemetacin, amtolmetin guacil, aspirin, bronfenac,

bufexamac, celocoxib, choline salicylate, clonixin, dexibuprofen, dexketoprofen, diclofenac, diflunisal, dipyrone, droxicam, etodolac, etofenamate, etoricoxib, felbinac, fenoprofen, fepradinol, feprazone, floctafenine, fenoprofen, fepradinol, feprazone, floctafenine, flufenamic acid, flurbiprofen, ibuprofen, indomethacin, ketoprofen, ketorolac, lornoxicam, loxoprofen, lumiracoxib, meclofenamate, mefenamic acid, meloxicam, morniflumate, nabumetone, naproxen, nepafenac, niflumic acid, nimesulide, nimesulide beta cyclodextrin, oxaprozin, oxyphenbutazone, parecoxib, phenylbutazone, iketoprofen, piroxicam, proglumetacin, propifenazone, proquazone, rofecoxib, salicylic acid, salsalate, sodium salicylate, sulindac, tenoxicam, tiaprofenic acid, tolfenamic acid, tolmetin and valdecoxib; Digitalis toxicity (nausea, vomiting, arrhythmias): acetyldigoxin, deslanoside, digitalis, digitoxin, digoxin and methyldigoxin; Reduction in blood pressure: alacepril, benazepril, captopril, cilazapril, delapril, enalapril, enalaprilat, fosinopril, imidapril, lisinopril, moexipril, pentopril, perindopril, quinapril, ramipril, spirapril, temocapril, trandolapril and zofenopril; Changes in echocardiogram or hypokalemia: albuterol, levalbuterol and vilanterol;

Increased risk of phototoxic skin reaction (i.e. severe sunburn): aminolevulinic acid; Increased risk of QT interval prolongation and torsade de pointes: Arsenic trioxide; Hypokalemia and subsequent cardiotoxicity (torsades de pointes): bepridil; Increased serum calcium levels resulting in hypercalcemia: calcitriol; Increased risk of hypercalcemia: calcium; Increased risk of myelosuppression: cyclophosphamide; Increased risk of hyponatremia: desmopressin; Increased risk of hyperglycemia: diazoxide; Increased risk of cardiotoxicity (QT prolongation, torsades de pointes, cardiac arrest): dofetilide, droperidol and sotalol; Risk of hypokalemia and hypomagnesemia: dronedarone; Increased risk of electrolyte imbalance and subsequent cardiotoxicity: flecainide; Increased risk of hypokalemia: gossypol and hydrocortisone; Ventricular arrhythmias: ketanserin; Increased risk of QT interval prolongation: levometadil; Increased risk of hypokalemia and/or reduced diuretic efficacy: licorice; Increased lithium concentrations and lithium toxicity (weakness, tremor, excessive thirst, confusion): lithium; Reducing the hypotensive effect of thiazide diuretics: Ma Huang; Decreased efficacy of diuretics: morphine, morphine sulfate liposome and oxycodone; Reduced hypoprothrombinemic effect of warfarin: warfarin Reduced diuretic efficacy: Ioimbine.

Contraindications

Chlorthalidone is absolutely contraindicated for patients with hypersensitivity to it or to drugs derived from sulfonamides, patients with severe hypokalemia and hyponatremia and anuria (KERNDT, 2021).
It is relatively contraindicated for patients with advanced chronic kidney disease, orthostatic hypotension, syncope, hypercalcemia and severe hyperuricemia or gout. Elderly patients aged 65 or over should avoid it due to the risk of hyponatremia (KERNDT, 2021). It is also contraindicated for pregnant women (OLIVEIRA, 2009; KERNDT, 2021).According to Micromedex, chlorthalidone is contraindicated in cases of anuria and hypersensitivity to chlorthalidone or other sulfonamide-derived drugs (MICROMEDEX_DRUGDEX_CLORTALIDONE).

Amlodipine besylate

Amlodipine besylate is a salt of amlodipine, a synthetic dihydropyridine that inhibits the influx of calcium ions from the extracellular spaceinto the intracellular space of peripheral vascular smooth muscle and myocardial cells, preventing vasoconstriction and myocardial contraction resulting in antihypertensive and antianginal effects (PUBCHEM_besilato de anlodipino_60496).

Pharmacokinetics

Physicochemical properties

Chemical name: benzenesulfonic acid; 3-O-ethyl 5-O-methyl 2-(2-aminoethoxymethyl)-4-(2-chlorophenyl)-6-methyl-1,4-dihydropyridine-3,5-dicarboxylate (PUBCHEM_besilato de anlodipino_60496).

Molecular formula: C26H31CIN2O8S (PUBCHEM_amlodipine besylate_60496).

Chemical structure:

Libbs

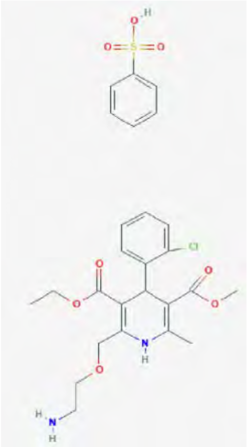

Figure 5: Chemical structure of amlodipine besylate
Source: Extracted from PUBCHEM_besilato de anlodipino_60496.

Pharmacodynamics

Mechanism of action

Amlodipine besylate is an L-type voltage-dependent calcium channel blocker, which acts by inhibiting the initial intracellular calcium influx. Reducing the concentration of intracellular calcium decreases vascular smooth muscle contraction, smooth muscle relaxation and, consequently, vasodilation. In addition, amlodipine besylate improves vascular endothelial function in hypertensive patients. It therefore lowers blood pressure by inducing vascular smooth muscle relaxation and vasodilation (BUSSARA, 2021).

Dosage

Dosage for adults

For the treatment of hypertension, the initial dose is 5 mg a day, with a maximum of 10 mg a day. Elderly patients should use a starting dose of 2.5 mg a day, with a maximum of 10 mg a day (BUSSARA, 2021).

Dosage for pregnant women

Pregnancy risk category C. There are not enough well-controlled studies in pregnant women. It crosses the placental barrier, however, in breast milk, concentrations are very low and it poses no risk to the infant (MORGAN, 2018).

Dosage for patients with renal insufficiency

The use of amlodipine in patients with chronic kidney disease at dosages of 2.5 - 5 mg is safe and used in this group of patients with arterial hypertension (IINO, 2004; SARUTA, 2009).

Adverse Events

The most significant adverse events are peripheral edema, heart failure, pulmonary edema, flushing, dizziness, headache, drowsiness, skin rash, nausea and abdominal pain (BULSARA, 2021).

Drug interactions

Strong CYP3A4 inhibitors (e.g. amiodarone, amitriptyline, atorvastatin, cimetidine, diclofenac, diltiazem, erythromycin, fluconazole, fluoxetine, norfloxacin, sertraline, verapamil) may increase plasma concentrations of amlodipine and caution should be exercised when co-administered with CYP3A4 inhibitors (BRAZ, 2018).vAccording to the Micromedex database, amlodipine may promote the following drug interactions (MICROMEDEX_DRUGDEX_ANLODIPINO): Increased CYP3A4 substrate exposure: abametapir, fexinidazole, fosnetupitant, netupitant and piperaquine; Increased amlodipine concentrations and increased risk of cardiotoxicity (prolonged PR interval): atazanavir; Increased exposure to CYP3A substrate: ceritinib and conivaptan; Increased exposure to amlodipine: clarithromycin; Decreased antiplatelet effect and increased risk of thrombotic events: clopidogrel; Increased risk of cyclosporine toxicity (renal dysfunction, cholestasis, paresthesias): cyclosporine; Severe hyperkalemia with cardiovascular collapse: dantrolene; Increased risk of complete heart block: digoxin; Increased

exposure to domperidone and increased risk of QT interval prolongation: domperidone;
Increased risk of cardiotoxicity (QT prolongation, torsades de pointes, cardiac arrest): droperidol; Increased risk of heart failure: epirubicin; Increased exposure to amlodipine; increased risk of imatinib toxicity: imatimibe; Increased exposure to amlodipine: ritonavir;Increased plasma concentrations of calcium channel blockers: indinavir; Increased risk of PR interval prolongation, AV block, bradycardia and ventricular tachyarrhythmia: lacosamide; Increased exposure to lomitapide: lomitapide; Reduction in the hypotensive effect of calcium channel blockers: Ma Huang, rifapentine and yohimbine; Reduced effectiveness of amlodipine: rifampicin; Increased exposure to simvastatin and increased risk of myopathy, including rhabdomyolysis: simvastatin; Increased exposure to tacrolimus: tacrolimus; Decreased exposure to 5-fluorouracil: tegafur; Increased plasma concentrations of amlodipine; telaprevir.

Contraindications

Amlodipine besylate must not be used in patients who are hypersensitive to amlodipine or any of the ingredients. In addition, amlodipine is relatively contraindicated in patients with cardiogenic shock, severe aortic stenosis, unstable angina, severe hypotension, heart and liver failure (BULSARA, 2021). According to Micromedex, amlodipine besylate is contraindicated in patients with known sensitivity to the drug (MICROMEDEX_DRUGDEX_ANLODIPINO).

Candesartan cilexetil, chlorthalidone and amlodipine besylate

Pharmacokinetics

Physical-Chemical Properties
Chemical name:

Candesartan cilexetil (prodrug): 1-cyclohexyloxycarbonyloxyethyl 2-ethoxy-3-[[4-[2-[2-(2H-tetrazol-5yl)phenyl]phenyl]methyl] benzimidazole-4-carboxylate (PUBCHEM_candesartana cilexetila_2540). Candesartan (active drug): 2-ethoxy-3-[[4-[2-[2-(2H-tetrazol 5-yl)phenyl]phenyl]phenyl]methyl]benzimidazole-4-carboxylic acid (PUBCHEM_candesartana_2541). Chlorthalidone: 2-chloro-5-(1-hydroxy-3-oxo-2H-isoindol-1-yl)benzenesulfonamide (PUBCHEM_chlortalidone_2732). Amlodipine besylate: benzenesulfonic acid; 3-O-ethyl 5-O-methyl 2-(2-aminoethoxymethyl)-4-(2-chlorophenyl)-6-methyl-1,4-dihydropyridine-3,5-dicarboxylate (PUBCHEM_Amlodipine besylate 60496).

Molecular formula:

Candesartan cilexetil: C33H34N6O6 (PUBCHEM_candesartan cilexetil, 2540).
Candesartan: C24H20N6O3 (PUBCHEM _candesartan_2541).
Chlorthalidone: C14H11CIN2O4S (PUBCHEM_clortalidona_2732).
Amlodipine besylate: C26H31CIN2O8S (PUBCHEM_anlodipine besylate 60496).

Chemical structure: The chemical structures of each of the isolated molecules have been recorded earlier in this document.

Molecular weight:

Candesartan cilexetil: 610.7 g/mol (PUBCHEM_candesartan cilexetil, 2540).
Candesartan: 440.5 g/mol (PUBCHEM _candesartan_2541).
Chlorthalidone: 338.8 g/mol (PUBCHEM_chlortalidone_2732).
Amlodipine besylate: 567.1 g/mol (PUBCHEM_anlodipine bezylate 60496).

Pharmacodynamics

Mechanism of action

Candesartan is a non-peptide angiotensin II receptor blocker which selectively binds to and slowly dissociates from the angiotensin II subtype 1 (AT1) receptor, thereby inhibiting the activity of angiotensin II in the receptor. Candesartan It has no activity on the subtype 2 receptor (AT2) (MCCLELLAN, 1998).

Chlorthalidone is an inhibitor of the sodium-chloride co-transporter in the distal convoluted tubule of the

nephron, reducing the reabsorption of sodium and chloride. By inhibiting sodium reabsorption, the distal convoluted tubule of the nephron retains a high concentration of sodium. The lack of sodium reabsorption alters the osmotic gradient and changes the distribution of fluid from the outside to the inside of the tubule. The increase in osmotic load due to the increase in sodium concentration leads to an increase in intratubular volume, thus promoting the diuretic effect. The increased excretion of sodium and extracellular fluids decreases the concentration of water and intravascular solutes. By reducing the intravascular volume and osmotic gradient, the patient suffers a reduction in hydrostatic pressure, allowing blood pressure to be reduced (KERNDT, 2021).

Amlodipine besylate is an L-type voltage-dependent calcium channel blocker, which acts by inhibiting the initial intracellular calcium influx. Reducing the concentration of intracellular calcium decreases vascular smooth muscle contraction, smooth muscle relaxation and, consequently, vasodilation. In addition, amlodipine besylate improves vascular endothelial function in hypertensive patients. It therefore lowers blood pressure by inducing vascular smooth muscle relaxation and vasodilation (BULSARA, 2021).

Dosage

Dosage for adults
The recommended dosage for adults is candesartan cilexetil 16 mg + chlorthalidone 12.5 mg + amlodipine 5 mg once a day.

Dosage for pregnant women
Pregnant women will not be included in the group for therapeutic indication in the treatment of gestational hypertension, as all three molecules pose a risk to the fetus. Candesartan cilexetil, chlorthalidone and amlodipine besylate are classified as risk category D, B and C in pregnancy, respectively (ALWAN, 2005; OLIVEIRA, 2009; MORGAN, 2018).

Dosage for patients with renal insufficiency
A search of the PubMed and Cochrane Library databases was carried out in October 2021 and no data was found regarding dosage for patients with renal insufficiency related to the use of the three molecules concomitantly in the combination described in this document.

Adverse Events

The adverse events reported below were those observed in the clinical studies.In the study designed to evaluate the pharmacokinetic interaction of chlorthalidone (12.5 mg tablet, marketed by EMS S.A.) administered alone or in combination with candesartan cilexetil (Atacand®, 16 mg candesartan cilexetil tablet, marketed by AstraZeneca do Brasil Ltda) and amlodipine besylate (Norvasc®, 5 mg anlodipine besylate tablet, manufactured by Laboratórios Pfizer Ltda), adverse events were observed: 1) expected - headache, nausea, vomiting; 2) change in expected laboratory tests - gamma-glutamyltranspeptidase (GGT) (81.3 U/L), glucose (129.8 mg/dL), oxalacetic transaminase (TGO) (65.9 U/L); 3) unexpected - colic; 4) unexpected laboratory test changes - Urine I (red blood cells 20000 to 60000/mL) (SCENTRYPHAR_Relatório interno_código 03/20, 2021).

In the study designed to evaluate the pharmacokinetic interaction of candesartan cilexetil (Atacand®, 16 mg candesartan cilexetil tablet, marketed by AstraZeneca do Brasil Ltda) administered alone or in combination with chlorthalidone (12.5 mg tablet, marketed by EMS S. A.) and amlodipine besylate (Norvasc®, 5 mg amlodipine besylate tablet, manufactured by Laboratórios Pfizer Ltda), the following adverse events were observed: 1) expected - headache, hypotension, dizziness, tinnitus, malaise, darkening of the eyes, intermittent dysuria, sweating, weakness; 2) changes in laboratory tests expected, pyruvic transaminase (TGO) (45.3 U/L), TGP (60.2 U/L), GGT (46.5 U/L); 3) unexpected - intermittent fever, low back pain, continuous dysuria; 4) unexpected laboratory test changes - Urine I (leukocytes 640000/mL), Urine I (red blood cells 20000 to 150000) (SCENTRYPHAR_Relatório interno_código 02/20, 2021).
In a study designed to evaluate the pharmacokinetic interaction of amlodipine besylate (Norvasc®, 5 mg amlodipine besylate tablet, manufactured by Laboratórios Pfizer Ltda) administered alone or in combination with chlorthalidone (12.5 mg tablet, marketed by EMS S.A.) and candesartan (Atacand®, 16 mg tablet, marketed by AstraZeneca do Brasil Ltda.), the results were as follows: 1) expected - headache, hypotension; 2) unexpected - asymptomatic hypertensive peak; 3) unexpected laboratory test changes - Urine I (red blood cells 30000 to 160000/mL) (SCENTRYPHAR_Relatório interno_código 04/20, 2021).

Drug Interactions

A search of the PubMed and Cochrane Library databases was conducted in October 2021 and no data was found on drug interactions related to the use of the three molecules concomitantly described in this document. In addition, all the drug

interaction data for each of the isolated molecules has been described above. In addition, pharmacokinetic interaction analysis studies (STPh 02/20, STPh 03/20 and STPh 04/20) were conducted and submitted for evaluation by the Therapeutic Equivalence Coordination - CETER on 06/15/2021 (Subject: 10839 - Pharmacokinetic Interaction Studies for Approval in Clinical Trials - Process: 25351625436202190, file: 2320536219). Proof of application can be found in the Development Plan.

The process is awaiting analysis of compliance with a requirement submitted on 01/20/2022 (Process: 25351625436202190, file: 0261276221), according to the proof of application attached to the Development Plan. CETER's final opinion (regarding the aforementioned pharmacokinetic interaction studies), once released, will be added to the application for approval in the Clinical Drug Development Case (CDDC) process.

Below are the summaries of the studies conducted:

Table: Summary of the studies

Design	Candesartan:<br>Open, randomized, 2-treatment, 2-sequence, 2-period, crossover, single dose.<br><br>Chlorthalidone:<br>Open, randomized, 2-treatment, 2-sequence, 2-period, crossover, single dose.<br><br>Amlodipine:<br>Open, randomized, 2-treatment, 2-sequence, 2-period, crossover, single dose.
Objectives	Candesartan:<br>To evaluate the pharmacokinetic interaction of candesartan cilexetil administered alone compared to the concomitant administration of candesartan cilexetil, chlorthalidone and amlodipine besylate, after oral, fasting and single dose administration. The products to be used in the pharmacokinetic interaction evaluation will be: Atacand®, 16 mg tablet containing candesartan cilexetil, marketed by AstraZeneca do Brasil Ltda; Chlorthalidone 12.5 mg tablet, marketed by EMS S.A.; and Norvasc® 5 mg tablet of anlodipine besylate, marketed by Laboratórios Pfizer Ltda.<br><br>Chlorthalidone:<br>To evaluate the pharmacokinetic interaction of chlorthalidone administered alone compared to the concomitant administration of candesartan cilexetil, chlorthalidone and amlodipine besylate, after oral administration, on an empty stomach and in a single dose. The products to be used in the pharmacokinetic interaction evaluation will be: Atacand®, 16 mg tablet containing candesartan cilexetil, marketed by AstraZeneca do Brasil Ltda; Chlorthalidone 12.5 mg tablet, marketed by EMS S.A.; and Norvasc® 5 mg tablet of amlodipine besylate, marketed by Laboratórios Pfizer Ltda.<br><br>Amlodipine:<br>To evaluate the pharmacokinetic interaction of amlodipine besylate administered alone compared to the concomitant administration of candesartan cilexetil, chlorthalidone and amlodipine besylate, after oral, fasting and single-dose administration. The products to be used in the pharmacokinetic

	interaction evaluation will be: Atacand®, 16 mg tablet containing candesartan cilexetil, marketed by AstraZeneca do Brasil Ltda; Chlorthalidone 12.5 mg tablet, marketed by EMS S.A.; and Norvasc® 5 mg tablet of amlodipine besylate, marketed by Laboratórios Pfizer Ltda.
Conclusion	Candesartan:<br>Considering that the limits of the confidence intervals (90%) of the estimates of the ratios between the geometric means of Cmax and ASC0-t, for the test formulation of candesartan, administered concomitantly with the formulations of chlorthalidone and amlodipine besylate, and the reference formulation, candesartan, administered alone, are within the range established by RE 1170 (April 19, 2006/ANVISA), it is concluded that there is no drug interaction with the administration of the formulation of Atacand®, 16 mg candesartan cilexetil tablet, marketed by AstraZeneca do Brasil Ltda. when administered concomitantly with the formulations of Norvasc®, 5 mg anlodipine besylate tablet and Chlorthalidone, 12.5 mg tablet, marketed by EMS S.A.<br><br>Chlorthalidone:<br>Considering that the limits of the confidence intervals (90%) of the estimates of the ratios between the geometric means of Cmax and ASC0-t, for the test formulation of chlorthalidone, administered concomitantly with the formulations of candesartan and amlodipine besylate, and the reference formulation, Chlorthalidone, administered alone, are within the bioequivalence range established by RE 1170 (April 19, 2006/ANVISA), it is concluded that there is no drug interaction with the administration of the formulation of Chlorthalidone, 12.5 mg tablet, marketed by EMS S. A., when administered concomitantly with the formulations of Atacand®, 16 mg candesartan cilexetil tablet, marketed by AstraZeneca do Brasil Ltda. and Norvasc®, 5 mg amlodipine besylate tablet, manufactured by Pfizer Ltda.<br><br>Amlodipine:<br>Considering that the confidence intervals (90%) of the estimates of the ratios between the geometric means of the treatments T (Test) and R (reference) for the parameters Cmax and ASC0-t are within the range established by RE 1170 (April 19, 2006/ANVISA), it can be concluded that the Norvasc® formulation administered concomitantly with the formulations of Atacand®, 16 mg tablet, containing candesartan cilexetil, marketed by AstraZeneca do Brasil Ltda. and Chlorthalidone, 12.5 mg tablet, marketed by EMS S.A., it is concluded that there is no drug interaction between amlodipine administered concomitantly with

	candesartan and chlorthalidone and the administration of this drug alone.
Comparators	Atacand®, 16 mg tablet containing candesartan cilexetil, marketed by AstraZeneca do Brasil Ltda; Chlorthalidone 12.5 mg tablet, marketed by EMS S.A.; and Norvasc® 5 mg tablet of amlodipine besylate, marketed by Laboratórios Pfizer Ltda.
Dosage of the experimental drug	There was no experimental drug as this was a pharmacokinetic interaction evaluation study.
Dosage of the comparators	Candesartan:<br>TREATMENT 1: 01 tablet of the drug Candesartan 16mg.<br>TREATMENT 2: 01 tablet of the drug Candesartan 16mg; 01 tablet of the drug chlorthalidone 12.5 mg; and 01 tablet of the drug amlodipine besylate 5mg (formulations administered concomitantly).<br><br>Chlorthalidone:<br>TREATMENT 1: 01 tablet of the drug chlorthalidone 12.5 mg.<br>TREATMENT 2: 01 tablet of the drug Candesartan 16mg; 01 tablet of the drug chlorthalidone 12.5 mg; and 01 tablet of the drug amlodipine besylate 5mg (formulations administered concomitantly).<br><br>Amlodipine:<br>TREATMENT 1: 01 tablet of the drug amlodipine besylate 5mg.<br>TREATMENT 2: 01 tablet of the drug Candesartan 16mg; 01 tablet of the drug chlorthalidone 12.5 mg; and 01 tablet of the drug amlodipine besylate 5mg (formulations administered concomitantly).
Pharmaceutical form of the experimental drug	There was no experimental drug as this was a pharmacokinetic interaction evaluation study.
Population	Healthy male and female research participants aged between 18 - 50 years old.

CONTRA INDICATIONS

A search of the PubMed and Cochrane Library databases was conducted in October 2021 and no data was found on contraindications related to the use of the three molecules concomitantly described in this document. In addition, all the contraindications for each of the isolated molecules have been described above.

REPORT OF NON-CLINICAL TRIALS

Summary
There is a possible beneficial synergy between candesartan and amlodipine (CHENG, 2018).

Acute Toxicity Study

In October 2021, the PubMed database was searched for articles evaluating the use of the combination of candesartan, chlorthalidone and amlodipine in acute non-clinical toxicity. Below is a table showing the search strategies and the number of results:

Table 1: PubMed database search strategies and number of results.

**Search strategy**	**Quantity of studies found**
chlorthalidone AND candesartan AND amlodipine AND animal	6
chlorthalidone AND candesartan AND animal	3
chlorthalidone AND amlodipine AND animal	4
candesartan AND amlodipine AND animal	30

The search did not identify any studies evaluating the acute toxicity of the combination. However, data on the acute non-clinical toxicity of each of the isolated substances was previously detailed in this document.

Repeated Dose Toxicity and Reproductive Toxicity

The same search strategies mentioned in the section on acute toxicity were used to identify studies evaluating the toxicity of repeated doses, as well as the non-clinical reproductive toxicity of the combination proposed here. The search did not identify any studies with these characteristics. In addition, data on the isolated substances evaluating these non-clinical safety parameters has already been presented.

Genotoxicity and carcinogenicity

No non-clinical studies were found evaluating the association in terms of genotoxicity or carcinogenicity. The searches were conducted using the search strategies described in the acute toxicity section. In addition, all the carcinogenicity and genotoxicity data for each of the isolated items has been described above.
Local Tolerance Study

In October 2021, a search was conducted aiming to identify studies evaluating whether the combination of candesartan, chlorthalidone and amlodipine is well tolerated. The search was conducted in the PubMed database using the strategies described above, which did not result in any relevant studies that could have been selected for this document.

Studies of Interest for the Evaluation of Pharmacological Safety

A study was conducted by researchers from the School of Pharmacy, Second Military Medical University, Shanghai, China, investigating the possible synergism of amlodipine with candesartan in reducing BP in hypertensive rats. Target organ protection was also observed. In the acute experiment, spontaneously hypertensive rats were treated with intragastric administration of amlodipine (0.5, 1, 2, 3 mg/kg), candesartan (1, 2, 3, 4, 6, 8 mg/kg) and 14 different combinations to find the possible proportion of synergistic interaction. The effects of amlodipine (1 mg/kg), candesartan (2 mg/kg) and their combination on BP reduction were also observed. In a chronic use study, rats were treated with amlodipine (1 mg/kg), candesartan (2 mg/kg) and their combination for five months. Organ damage was evaluated after recording BP. The probability sum test (q-test) was used to evaluate synergistic action. There is a synergistic interaction between amlodipine and candesartan in reducing BP. The synergistic effect was also confirmed in hypertensive rats. In the chronic study, this combination had obvious synergism in reducing BP and BP variability, as well as protection in target organs. Multiple regression analysis showed that cardiac and aortic hypertrophy indices and glomerular damage parameters were positively related to BP and BP variability.

In conclusion, the combination of amlodipine and candesartan exhibited a potent antihypertensive effect and possessed obvious synergism in BP lowering and organ protection in hypertension. BP reduction and BP variability may be an important contribution to target organ protection (CHENG, 2018).

The vascular protection effects of placebo, candesartan monotherapy (1 mg kg (-1) per day), combination therapy of candesartan (1 mg kg (-1) per day) and amlodipine (1 mg kg (-1) per day), or combination therapy of candesartan (1 mg kg (-1) per day) with hydrochlorothiazide (10 mg kg (-1) per day) for two weeks in stroke-prone, spontaneously hypertensive rats were compared. Candesartan monotherapy significantly reduced blood pressure and both combination therapies demonstrated similar results and significantly lower than monotherapy. Acetylcholine-induced

vascular relaxation was significantly stronger in all therapy groups than in the placebo-treated group. In addition, relaxation was significantly stronger in the group treated with candesartan in combination with amlodipine than in the group treated with candesartan monotherapy; however, there was no significant difference between the groups treated with candesartan and candesartan with HCTZ. Vascular gene expressions of the NADPH oxidase subunits p22 (phox), gp91 (phox), NOX1 and NOX4 were significantly attenuated in all therapeutic groups compared to the group receiving placebo, and there were no significant differences between these groups. However, a significant increase in vascular superoxide dismutase activity was observed in the group treated with candesartan associated with amlodipine, but not in the other groups. Malondialdehyde levels in vascular tissues were significantly attenuated in all therapeutic groups. Compared to the group treated with candesartan, significant attenuation was observed in the group treated with candesartan associated with amlodipine, but not in the group treated with candesartan associated with HCTZ. Immunohistological analysis showed that the areas positive for 4-hydroxy-2-nonenal were significantly reduced in all therapeutic groups, but this reduction was significantly greater for the group treated with candesartan and amlodipine than for the group treated with candesartan monotherapy. Thus, the combined therapy of candesartan and amodipine may have a powerful protective effect on vascular tissues by reducing oxidative stress (TAKAI, 2011). The experiments were conducted in accordance with the Guide for the Care and Use of Laboratory Animals (Animal Research Laboratory, Osaka Medical College, Osaka, Japan).The animal model (rat) is consistent with that recommended in the OECD guides for non-clinical toxicity studies (OECD/OECD, 2001; OECD/OECD, 2018a).

Toxicokinetics studies

No non-clinical studies were found evaluating the toxicokinetics parameters of the combination of candesartan, chlorthalidone and amlodipine. However, data on the non-clinical toxicokinetics of each of the isolated substances has been detailed earlier in this document.

Discussion and conclusions on non-clinical data

Although there is no vast non-clinical literature evaluating the use of the combination in studies, some studies have been found evaluating pharmacodynamic aspects of the combination of candesartan with amlodipine, pointing to synergy and a possible beneficial effect between these two molecules. This indicates a potential benefit in hypertensive patients (CHENG, 2018; TAKAI, 2011). The animal model used in these studies (rat) is aligned with the guidelines for OECD non-clinical toxicity studies (OECD/OECD, 2001; OECD/OECD, 2018a).

CLINICAL TRIALS REPORT

Literature search methodology

To identify the clinical studies, a systematic search was conducted on 09/21/2021 to find articles indexed in the Medline (PubMed) database, published in Portuguese, English and Spanish.
This document was composed of studies published in the last ten years evaluating the use of the combination of candesartan cilexetil, chlorthalidone and amlodipine bensilate, or two of the components of the combination, aiming to provide relevant, up-to-date and clinically relevant data.
The search strategies described below were used to identify clinical studies evaluating the use of the combination in patients with systemic arterial hypertension:

Table 2: Search strategies

Search strategies
candesartan cilexetil AND amlodipine besylate Filters: in the last 10 years
chlorthalidone AND candesartan cilexetil Filters: in the last 10 years
chlorthalidone AND amlodipine besylate Filters: in the last 10 years
candesartan cilexetil AND amlodipine besylate AND chlorthalidone

Selection of studies identified in the literature
Out of the total number of studies located (204), 19 studies that met the inclusion and exclusion criteria were selected as described below:

Table 3: Inclusion and exclusion criteria

Inclusion criteria
Randomized controlled clinical trials
Systematic reviews of randomized controlled trials
Exclusion criteria
Association with molecules other than the proposed combination
Literature review without systematic review or meta-analysis
Non-clinical studies (conducted on animals, *in vitro* or *in vivo*)
Dosage regimen different from that proposed
Observational studies
Case reports, consensus, clinical study protocols or post-hoc analyses
Studies whose main objective is not to evaluate the safety and efficacy of the molecule in the treatment of hypertension
Bioequivalence or pharmacokinetic or pharmacodynamic study
Study conducted in a population other than the one proposed

Description of the selected studies

In 2017, a study was conducted to analyze the optimal dosage of a fixed-dose combination of candesartan cilexetil (CAN) and amlodipine besylate (AML), examining the tolerability and efficacy of CAN/AML combination therapy compared to monotherapy in patients with essential hypertension. This multicenter, randomized, double-blind Phase II clinical trial was conducted in patients aged 19 and over with essential hypertension. A total of 635 patients were screened, of whom 439 were randomized to receive treatment; 425 patients were included in the full analysis set (combination therapy, 212; monotherapy, 213). Participants were randomized to receive 1 of 8 treatments: CAN (8 or 16 mg), AML (5 or 10 mg), CAN/AML (8 mg/5 mg, 8 mg/10 mg, 16 mg/5 mg or 16 mg/10 mg), once daily for eight weeks. After eight weeks of treatment, changes in msDBP were significantly greater in the groups receiving combined CAN/AML therapies compared to monotherapies at corresponding doses, with the exception of CAN 8 mg/AML 10 mg versus AML 10 mg. The response to treatment and the achievement of target BP (both msSBP and msDBP) at week eight were significantly higher overall in the groups receiving combination therapy versus monotherapy. All drugs were relatively well tolerated in each group.

The researchers concluded that administration of CAN/AML for eight weeks (8 mg/5 mg, 16 mg/5 mg and 16 mg/10 mg) resulted in a significantly greater reduction in BP than with candesartan cilexetil or amlodipine monotherapies, and was well tolerated (SOHN, 2017). Clinical study with level of evidence 1B and grade of

recommendation A, according to the Oxford criteria. As pointed out by the authors, the study was limited by its short treatment duration of 8 weeks and the relatively small number of hypertensive patients. It is worth noting that this study was previously cited in the list of studies conducted on candesartan cilexetil monotherapy, as well as on amlodipine monotherapy.

To compare the efficacy and safety of various combination therapies of ARBs with amlodipine and amlodipine monotherapy for the treatment of hypertension in the Asian population, a systematic review was conducted with randomized controlled clinical studies. This work was previously described in the list of clinical studies conducted with amlodipine monotherapy. The search was conducted in the PubMed and Cochrane Libraries.

The primary efficacy and safety endpoints were response to short-term treatment (8 - 12 weeks) and TEAE, respectively. The analysis included 1,198 Asian patients with hypertension from seven studies with six different ARBs associated with amlodipine: azilsartan, candesartan cilexetil (CAN), fimasartan, losartan, olmesartan and telmisartan. Compared to amlodipine monotherapy, azilsartan-anlodipine combination therapy was five times more likely to induce a response to treatment (OR 5.2, 95% CI: 2.5, 11.2), while the CAN-amlodipine combination was 3.9 (95% CI: 2.5, 6.4), while fimasartan-amlodipine was 3.4 (95% CI: 1.4, 8.5), telmisartan-amlodipine was 3.3 (95% CI: 1.6, 7.1), olmesartan-amlodipine was 2.7 (95% CI: 1.6, 5.0) and losartan-amlodipine was 2.0 (95% CI: 0.6, 7.3). All ARB-amlodipine combination therapies demonstrated comparable safety profiles to amlodipine monotherapy, except telmisartan-amlodipine, which demonstrated significantly lower odds of AEETs (0.26 (95% CI: 0.087, 0.70). The safety profiles between ARB-amlodipine combination therapies were broadly comparable (LEE, 2019). Systematic review of randomized controlled trials, therefore level of evidence 1A and grade of recommendation A according to Oxford criteria. Due to the limited size of the study and the small number of trials (direct evidence), the results should be better interpreted as an important exploratory effort and hypothesis generation to inform the direction of future research.

See the brochure for a table containing the main information on the clinical studies, with the association of two of the three components of this triple therapy, selected for this document. The table includes the following data: reference in ABNT format, study design, treatment, comparator, conclusion and Oxford level of evidence.

Discussion and conclusions on the clinical data

There is a notable lack of clinical studies on the association proposed here. According to a literature search in the PubMed database, following very well-defined inclusion and exclusion criteria mentioned earlier in this paper, only one study was found to make up the list of clinical studies on the association.

Even so, the aforementioned article only evaluates the combination of two of the three molecules in our association. A major limitation is the lack of literature covering all the molecules within the defined dosage regimen. This indicates the importance and necessity of conducting a clinical study with this combination.

The study evaluating the combination of amlodipine and candesartan (SOHN, 2017) is a study with a high level of evidence in the literature (1B), but its limitations are the small number of participants and consequent limited statistical power. Even so, the evidence is favorable to the association.

One study was added (LEE, 2019) which, although it was not identified in the search for studies with the association, according to the search strategies defined, was included because it was considered clinically relevant. It is a systematic review with the best level of evidence in the literature and addresses the use of amlodipine with different ARBs, including candesartan, pointing to the efficacy and safety of the association.

RATIONALE FOR THE EXPERIMENTAL DRUG

According to the World Health Organization, treatment adherence is defined as the extent to which a person's behaviors correspond to the agreed recommendations of a healthcare provider, with the adherence to the prescribed medication as an essential part of this (SABATÉ, 2003).With regards to the treatment of hypertension in Brazil, only 50% of hypertensive patients are fully aware of their condition, 42% adhere to treatment and only 10% have their blood pressure within controlled levels (ZANCHETTI, 2001). Several reasons can be listed for the failure to adhere to drug treatment for hypertension: clinical inertia, adverse events, use of ineffective protocols, non-use of combined drug strategies and treatment costs (VOLPE, 2020). The use of inefficient or ineffective protocols in the treatment of hypertension results in a constant switch of drugs and doses, higher costs for the patient, longer travel time to clinical appointments and a generalized feeling of ineffectiveness in the treatment, triggering low adherence to it (JOHNSTON, 2010; SALAM, 2014).

Libbs

Contemporary medications for the treatment of AH tend to require combined strategies of two or more drugs for BP control and prevention of future cardiovascular events, as described in the most recent guidelines for its treatment (BARROSO 2020, WILLIAMS, 2018). Based on the greater effectiveness of combination therapy, using two or more antihypertensive drugs, and greater adherence to treatment, studies have been conducted to evaluate thethe effectiveness and acceptance of a fixed-dose combination of three antihypertensive drugs (EGAN, 2012, SALAM, 2014, VOLPE, 2020, WANG, 2020a).

According to the Brazilian Hypertension Guideline, hypertension treatment should be initiated with a dual combination of drugs with different mechanisms of action. If the blood pressure target is not achieved, dose adjustments and/or a triple combination of drugs are indicated. More drugs should then be added until BP control is achieved (BARROSO 2020). In addition, the combination of drugs may potentially reduce the occurrence of adverse events, avoiding the need for successive dose increases of the components currently in use and due to the ability of one of the drugs to antagonize the adverse effects of the other.

Egan et al demonstrated, from a database of more than 100,000 hypertensive patients distributed in 180 centers in the United States, that the initiation of treatment for AH using a combination of drugs in a single pill improved the achievement of BP control by 53% compared to the corresponding drug combination (EGAN, 2012).

The combination of a calcium channel blocker drug, which inhibits the transmembrane influx of calcium ions in vascular smooth muscle and cardiac muscle, with an angiotensin II receptor blocker, which inhibits angiotensin II-mediated vasoconstriction and renal sodium retention, and a low-dose thiazide diuretic, which reduces intravascular volume and total body sodium, which inhibits angiotensin II-mediated vasoconstriction and renal sodium retention, and a low-dose thiazide diuretic which reduces intravascular volume and total body sodium, (BCC+ARB+DIU combination) has a synergistic effect of ARB+DIU and BCC+BRA, providing a superior antihypertensive effect in combination when compared to the active ingredients alone (CALHOUN, 2009; ALLEMANN, 2008; LEE, 2020; BRAMLAGE, 2018).

In addition, Volpe et al. (2020) in a literature review, state that the addition of a renin-angiotensin-aldosterone system inhibitor (ACEI or ARB) to a thiazide diuretic not only has an additive effect on BP reduction, but also balances out some of the adverse effects of diuretics related to electrolyte profile (hypokalemia), uric acid and glucose metabolism. With regards to the combination of ARBs and BCCs, ARBs cause capillary dilation, both arteriolar and venular, with increased reabsorption of interstitial fluid, thus reducing the peripheral edema promoted by CCBs. On the other hand, CCBs, like diuretics, cause a counter-regulatory increase in ARB activation with a consequent and synergistic BP-lowering effect due to greater efficacy (VOLPE, 2020).

Also based on these considerations, European guidelines suggest that triple drug combinations should generally include an ACEI or ARB, a dihydropyridine CCB and a thiazide diuretic (WILLIAMS, 2018). At the same time, the Brazilian Hypertension Guidelines - 2020 and the 7th Brazilian Hypertension Guideline recommend combining a calcium channel blocker such as amlodipine with an angiotensin receptor blocker such as candersatan and a diuretic such as chlorthalidone. It has been shown to be safe and effective (BARROSO, 2020; MALACHIAS, 2016).

Several clinical studies have reported a consistent superiority of triple therapy in terms of BP reductions in and out of the office (LAW, 2013). It should be noted that a greater number of agents used in combination is not associated with a greater number of adverse events. In this sense, the benefits of a triple combination of ARBs, CCBs and diuretics are also supported by the evidence that the rates of the most common adverse events, such as mild to moderate and well-tolerated dizziness, cough, peripheral edema and headache, are comparable to those related to the two-drug combinations used for comparisons (VOLPE, 2020).

In this context, a triple combination of amlodipine (calcium channel blocker) at a dosage of 5 mg, candesartan cilexetil (angiotensin receptor blocker) at 16 mg and the diuretic chlorthalidone at 12.5 mg, in a single pill containing the three antihypertensive drugs, is justified by the beneficial synergistic effect, as well as the increase in patient compliance.

Also, according to the Brazilian Hypertension Guidelines, the lowest dose of chlorthalidone available on the Brazilian market is 12.5 mg; higher doses increase the diuretic effect without adding pertinent antihypertensive action. In addition, in a study by Musini et al. (2014), doses of 75 mg/day of chlorthalidone did not promote a greater reduction in blood pressure compared to a dose of 50 mg/day, which was also not superior to a dose of 25 mg/day or 12.5 to 15 mg/day. The 25 mg/day dose also did not promote a greater reduction compared to the 12.5 mg/day dose (MUSINI, 2014).

According to Micromedex - a decision-making support and reference tool designed to support clinical work in the field of patient care - the dosage of amlodipine recommended by the Food and Drug Administration (FDA) for the

initial treatment of hypertension is 5 mg orally once a day (Micromedex_Drugdex_anlodipine).

As for the use of candesartan cilexetil, the Micromedex base indicates that, in the treatment of arterial hypertension, the recommended initial dosage is 16 mg orally once a day, or two 8 mg administrations (Micromedex_Drugdex_candesartan cilexetil). In addition to this data, in the Guide to the Registration of Fixed-Dose Combinations for the Treatment of Arterial Hypertension, published by Anvisa in 2010, Possible triple combinations on the positive list include both the combination of a thiazide diuretic or chlorthalidone + angiotensin II AT1 receptor blocker + dihydropyridine calcium channel blocker and the combination of a thiazide diuretic or chlorthalidone + renin inhibitor + dihydropyridine calcium channel blocker (ANVISA, 2010). In addition, in possible double combinations, amlodipine at a dosage of 5 or 10 mg may be combined with an angiotensin II AT1 receptor blocker, such as candesartan (ANVISA, 2010).

This justifies the dosage of 5 mg of amlodipine, 16 mg of candesartan cilexetil and 12.5 mg of chlorthalidone. This is because they are the lowest effective dosage (because they maintain adequate blood pressure) and the safest (because they mitigate the possibility of adverse events caused by administering high dosages). The pharmaceutical form containing the three drugs in the same tablet, in turn, favors adherence to treatment and, consequently, the efficacy of the drug.

JUSTIFICATION FOR CONDUCTING THE CLINICAL STUDY

The aim of this clinical study is to prove the efficacy and safety of the new fixed combination of candesartan cilexetil 16mg + chlorthalidone 12.5mg + aMlodipine 5mg in the treatment of systemic arterial hypertension in men and women.

As mentioned above and described in the investigator's brochure, there is a vast body of scientific literature on pre-clinical and clinical research that shows the efficacy and safety of the molecules. A non-inferiority study was therefore designed for Exforge HCT® (valsartan 160mg + hydrochlorothiazide 12.5mg + amlodipine 5mg).

Furthermore, considering situation 2 of the Guide to the Registration of New Fixed-Dose Combinations, in which the registered single-drugs formulations have a well-established efficacy and safety profile, as previously reported, and that the investigation product under study is a new fixed-dose combination that has not yet been studied, the need to conduct a phase III clinical study is justified.

It is estimated that 58% of hypertensive patients need more than one agent with differents mechanism of action to keep their blood pressure within the target (ANVISA, 2010). Associations of different agents in a single tablet are therefore preferred because they increase adherence to treatment, consequently providing better clinical results and reducing adverse events, since molecules with different mechanisms of action can antagonize the adverse reactions caused by another molecule present in the combination (ANVISA, 2010). In addition, it is possible to reduce the dosage of each of the substances, which also contributes to minimizing adverse events (BARROSO, 2020; JAMERSON, 2004; VOLPE, 2020).

To date, there are not many triple combinations for hypertension available in Brazil. Therefore, it is necessary to develop new combinations in order to provide to doctors and patients new treatment possibilities according to the needs of each patient. In addition, clinical studies have shown excellent results in blood pressure control comparing candesartan (angiotensin II receptor blocker) to valsartan (angiotensin II receptor blocker present in the triple combination available in Brazil) (NAKAJIMA,.2020). Nakajima, et.al. 2020 showed that the average change (95% confidence interval) from baseline in SBP/DPB for candesartan cilexetil and valsartan was 3.8 (2.9-4.8), 2.4 (1.1-3.8) mmHg, respectively, demonstrating satisfactory results in treatment with candesartan. The same was demonstrated in relation to chlorthalidone and hydrochlorothiazide, the former being more effective in reducing blood pressure as well as not increasing the incidence of hypokalemia, hyponatremia and any changes in blood glucose and total serum cholesterol (LIANG, 2017). Current guidelines encourage the use of pharmaceutical pills combining two or more antihypertensive drugs based on the fact that a triple combination offers greater savings and convenience for the patient, leading to greater treatment adherence (WILLIAMS, 2018; VOLPE, 2020; WEBSTER, 2018; SALAM, 2014).

The combination of a calcium channel blocker, inhibiting the transmembrane influx of calcium ions in vascular smooth muscle and cardiac muscle, with an angiotensin receptor blocker, inhibiting angiotensin II-mediated vasoconstriction and renal sodium retention, and a diuretic, reducing intravascular volume and total body sodium, (association BCC+ARB+DIU) presents a synergistic effect of ARB+DIU and BCC+ARB providing a superior antihypertensive effect in combination when compared to the active ingredients alone (CALHOUN, 2009; ALLEMANN, 2008; LEE, 2020; BRAMLAGE, 2018). Based on the information presented, the benefits in the management of pharmacological treatment and greater convenience and adherence to treatment, there is a technical justification for the

clinical development of this drug.

RISK AND BENEFITS ASSESSMENT

Drug risks and benefits assessment

Hypertension is defined as a chronic disease in which blood pressure levels are persistently increased on two or more occasions in the absence of antihypertensive drugs. It is a chronic, multifactorial disorder in which SBP is greater than or equal to 140mmHg and/or DBP greater than or equal to 90mmHg. When not treated correctly, it can have a significant impact on medical courses as it compromises various body systems and may lead to death. Some of the organs commonly affected are: theheart, increasing the risk of coronary artery disease, heart failure, atrial fibrillation and sudden death; the brain, increasing the occurrence of ischemic or hemorrhagic strokes and dementia; the kidneys, where may cause chronic kidney disease and require dialysis; and the arterial system, with the risk of causing peripheral obstructive artery disease (BARROSO, 2021).

The 2020 Brazilian Hypertension Guideline emphasizes the need for drug therapy to reduce the risks of mortality and morbidity caused by this condition (BARROSO, 2021). There are five main pharmacological classes of antihypertensive drugs: diuretics, calcium channel blockers, angiotensin-converting enzyme inhibitors (ACEIs), angiotensin II receptor blockers and beta-blockers. Alpha-blockers, centrally-acting sympatholytics, aldosterone antagonists and direct vasodilators are associated with a higher rate of adverse events and have not been widely tested in clinical trials, so more information is still needed (BARROSO, 2021; ANVISA, 2010).

Monotherapy is used as the initial treatment strategy, however, when the main objective of drug therapy - lowering blood pressure - is not achieved, it is necessary to combine two or three medications to control the disease. The rationale for combining agents is based on the association of drugs with different mechanisms of action, and there is no advantages in combining molecules whose mechanisms of action are similar (ANVISA, 2010). In addition, the combination of agents may be beneficial, considerably reducing the occurrence of side effects, since there is no need to use the highest doses of each molecule, and there may even be antagonism of the adverse effects of other drugs in the combination (MANCIA, 2019; PÓVOA, 2014).

In addition, studies show greater adherence to treatment when fixed doses are combined in a single pill, as well as a reduction in outcomes involving cardiovascular risk when compared to monotherapy. Thus, the combination of drugs in a single pill demonstrates better clinical results (BARROSO, 2021).

Therefore, in this protocol we aim to prove the therapeutic efficacy of the new combination of candesartan cilexetil 16mg + chlorthalidone 12.5mg + amlodipine 5mg in hypertensive individuals who do not respond to dual therapy. We will use Exforge HCT® (valsartan 160mg + hydrochlorothiazide 12.5mg + amlodipine 5mg) as a comparator. Both Exforge HCT® and the new combination contain the same 3 classes of agents most commonly used to the blood pressure control: angiotensin II receptor blockers + thiazide diuretics + calcium channel blockers.

Candesartan cilexetil is an angiotensin II receptor blocker and is selective for AT1-type receptors. AT1 receptor antagonism leads to an increase in plasma renin and angiotensin I and II concentrations. It also promotes a reduction in the plasma concentration of aldosterone and vasodilation, thus lowering BP. It is worth mentioning that candesartan cilexetil does not inhibit the angiotensin-converting enzyme which converts angiotensin I into angiotensin II, and also degrades bradykinin. Thus, the use of this drug is not associated with the cough commonly reported in patients using ACE inhibitors (ATACAND PACKAGE INSERT, 2015).

The adverse events reported in controlled clinical studies were mild and transient, comparable to placebo. With regards to laboratory findings, minor changes in hemoglobin levels, increases in creatinine, urea, potassium and decreases in sodium levels were reported. An increase in serum ALT levels has been reported as an adverse event, and patients with severe kidney insuficiency should be monitored (ATACAND BULA, 2015).

Few studies were found comparing the efficacy of candesartan cilexetil with other drugs in the same pharmacological class, including a systematic review by Nakajima et al. (2020) whose objective was to identify randomized clinical trials (RCTs) indexed in PubMed and Ichushi, in Japanese patients with hypertension receiving monotherapy with angiotensin II receptor blockers (azilsartan, candesartan cilexetil, irbesartan, losartan potassium, olmesartan medoxomila, telmisartan, valsartan) in at least one arm. Of the 763 studies identified, 77 met the eligibility criteria; of these, 37 reported a mean change in systolic blood pressure (SBP) and diastolic blood pressure (DBP). A fixed effects model (FEM) showed the effect of each drug in relation to the reference, azilsartan. Using the FEM, the mean

change (95% confidence interval) from baseline in SBP/DPB for candesartan cilexetil, irbesartan, losartan potassium, olmesartan medoxomil, telmisartan and valsartan was 3, 8 (2.9-4.8)/2.6 (2.0-3.1), 4.8 (2.0-7.5)/3.7 (1.8-5.6), 3.0 (0.8-5.1)/1.9 (0.5-3.3), 3.2 (1.2-5.1)/2.7(1.3-4.1), 3.2 (0.8-5.6)/2.0 (0.3-3.6) and 3.1 (1.1-5.1)/2.4 (1.1-3.8) mmHg, respectively. The results of this meta-analysis provide evidence that candesartan cilexetil showed the second greatest reduction in systolic blood pressure (mean change 3.8 mmHg) and that it has a more favorable efficacy profile in reducing SBP and DBP than most of the other drugs evaluated (NAKAJIMA, 2020).

Chlorthalidone is a long-acting thiazide diuretic whose main indication is hypertension. The molecule acts in the proximal region of the distal convoluted tubule, inhibiting NaCl reabsorption and promoting calcium reabsorption, as well as allowing potassium and hydrogen to be excreted. The most common adverse effects include: hyperkalemia, hyperuricemia, hyponatremia, hypomagnesemia, hyperglycemia, decreased appetite, vertigo, postural hypotension, abdominal discomfort, urticaria, rash and erectile dysfunction. Rare or very rare adverse reactions include: thrombocytopenia, leukopenia, agranulocytosis, eosinophilia, hypercalcemia, gout, inadequate control of diabetes mellitus, hypochloremic alkalosis, paresthesia, headache, visual problems, arrhythmias, vasculitis, non-cardiogenic pulmonary edema, nausea, vomiting, abdominal pain, constipation, diarrhea, pancreatitis, cholestasis, photosensitivity reactions, glycosuria and tubulo-interstitial nephritis (CLORTALIDONE BULA, 2021).

Several studies have been conducted to verify the efficacy of chlorthalidone compared to other thiazide diuretics, such as hydrochlorothiazide. These include the systematic review by Liang et al. (2017) who compiled the main randomized controlled clinical studies evaluating the efficacy of hydrochlorothiazide and similar thiazide diuretics (chlorthalidone and indapamide) in reducing blood pressure and their effects on blood electrolytes (incidence of hypokalemia and hyponatremia), glucose and total cholesterol. Twelve studies were identified: five comparing indapamide with hydrochlorothiazide and seven with chlorthalidone versus hydrochlorothiazide. Studies totaling 1,580 participants in 12 clinical trials were selected, of which 10 (N = 1,307) provided data on SBP reduction and 11 (N = 1,347) on DBP after treatment. In the group of similar thiazide diuretics, indapamide and chlorthalidone were evaluated in an integrated manner. A significant reduction in SBP (95% CI, -5.59 [-5.69, -5.49]; P < 0.001) and DBP (95% CI, -1.98 [-3.29, -0.66]; P = 0.003) was observed with similar thiazide diuretics (chlorthalidone and indapamide). In the analysis of adverse events, there were no statistically significant differences between the two groups in relation to the incidence of hypokalemia, hyponatremia, changes in blood glucose and total cholesterol. From these data, it was concluded that treatment with similar thiazide diuretics showed better results compared to hydrochlorothiazide in reducing blood pressure without increasing the incidence of hypokalemia, hyponatremia and any change in blood glucose and total serum cholesterol (LIANG, 2017).

Amlodipine is another first-choice drug for the treatment of hypertension. It can be used as monotherapy or in combination with thiazide diuretics, beta-blockers or ACE inhibitors. It acts by blocking calcium channels, thus inhibiting the transmembrane influx of calcium into the cardiac or vascular smooth muscle. As such, it is a direct relaxant of vascular smooth muscle, preventing vasoconstriction and the consequent rise in BP (NORVASC, 2021).

Amlodipine is generally well tolerated by patients who take it continuously. The most common adverse events are: headaches, dizziness, drowsiness, palpitations, flushing, abdominal pain, nausea, edema and fatigue. Rarely observed adverse events include: leukopenia, thrombocytopenia, hyperglycemia, insomnia, altered mood, syncope, extrapyramidal disorder, visual impairment, tinnitus, hypotension, vasculitis, cough, dyspnea, rhinitis, dry mouth, dyspepsia, pancreatitis, vomiting, urticaria, alopecia, purpura, myalgia, muscle spasms, back pain, gynecomastia, erectile dysfunction, asthenia, malaise, pain and weight gain or loss (NORVASC, 2021).

To compare the efficacy and safety of various combination therapies of angiotensin receptor blockers with amlodipine compared to amlodipine monotherapy for the treatment of hypertension, a systematic review was conducted with randomized controlled clinical trials. The primary efficacy and safety endpoints were short-term treatment response (8 - 12 weeks) and treatment-emergent adverse events, respectively. Monotherapy with amlodipine was used as a comparator. The analysis included 1198 Asian patients with hypertension from seven studies with six different angiotensin receptor blockers associated with amlodipine: azilsartan, candesartan cilexetil, fimasartan, losartan, olmesartan and telmisartan. Compared to amlodipine monotherapy, candesartan cilexetil-amlodipine combination therapy was 3.9 times more likely to induce a response to treatment (95% CI: 2.5, 6.4), making it the second-best combination, only behind azilsartan-amlodipine. The safety profiles between angiotensin-amlodipine receptor blocker combination therapies were broadly comparable (LEE, 2019).

Knowing that the treatment of arterial hypertension is still a technical challenge in daily clinical practice, we believe that a fixed-dose combination pill containing candesartan cilexetil 16mg + chlorthalidone 12.5mg + amlodipine 5mg is an important ally in the therapeutic arsenal for the treatment of this disease. This treatment follows current guidelines regarding the management of arterial hypertension, associating drugs with different mechanisms of action,

synergistic action, avoiding the need to increase the dose in a double association and maintaining good tolerability.

Therefore, the purpose of this clinical study is to demonstrate the credibility and validate the use of this combination, based on the results already presented and known, and respecting the indications and dosage of the package leaflets of the drugs that make up this combination.

STUDY RISK-BENEFIT ASSESSMENT

As previously mentioned, hypertension is a chronic, multifactorial disease which, when not properly controlled, can impair the functioning of various organs and increase the risk of morbidity and death. We will try to prove the therapeutic efficacy of the new fixed-dose combination through a clinical study in a population whose target pathology is hypertension.

The 2013 National Health Survey revealed a prevalence of 21.4% of adult Brazilians who self-declared as hypertensive. When considering the number of Brazilians who said they used antihypertensive drugs, this figure rose to 32.3% of the adults interviewed, with males being more affected by the disease. It is known that hypertension is more prevalent in the elderly. In this same survey, 71.7% of the elderly interviewed were hypertensive (MALTA, 2018). With regards to the costs to the SUS, these were higher than those attributed to obesity and diabetes mellitus, with US$ 523.7 million spent by the SUS in 2018 for the treatment of this disease (NILSON, 2018).

Thus, pharmacological treatment is a key point in the treatment of hypertension. This treatment aims to reduce systolic and diastolic blood pressure to normal levels. As mentioned, several pharmacological classes may be used to treat this disease, both in monotherapy and in combination therapies. However, according to the Guide to the Registration of Fixed-Dose Combinations for the Treatment of Arterial Hypertension, the ineffectiveness of monotherapy in many cases has already been demonstrated and the average number of patients requiring a drug combination was 58%, with most cases requiring the use of 3 antihypertensive agents (ANVISA, 2010).
The purpose of the fixed-dose combination is to provide patients a drug that simplifies administration by requiring only one tablet, as opposed to multiple concomitant drugs, enabling better adherence and, consequently, better results in blood pressure control. ANVISA's Guide to the Registration of Fixed-Dose Combinations for the Treatment of Arterial Hypertension also outlines the main advantages of drug combinations in the management of arterial hypertension, which are: increased antihypertensive efficacy; a lower incidence of adverse events; greater treatment adherence; achieving blood pressure targets; and an increase in the antihypertensive effect in specific populations (ANVISA, 2010).

In addition to the aforementioned drug-related events, participants may experience hematoma at the venipuncture site used for drawning blood for laboratory tests. Every precaution will be taken to reduce, control and/or eliminate these risks. The research participant will be constantly monitored by the study investigator, who will provide assistance in the event of any occurrence.
Regarding the collection and processing of data for scientific purposes, the study will respect current Brazilian data protection legislation and other Brazilian resolutions applicable to clinical trials, always respecting the confidentiality of data, privacy and non-stigmatization of research participants.

Any electronic or manual system is liable to fail during any clinical trial. However, the researcher responsible and his team must work continuously to ensure that there are no failures related to the confidentiality of research participants' information, and to this end eCRF will be used to mitigate the risk of breaches of data confidentiality, in addition to which participants' data will be duly coded and anonymized, guaranteeing the confidentiality of the information.

Therefore, we are aiming to prove the therapeutic efficacy of the new fixed-dose combination (candesartan cilexetil 16mg + chlorthalidone 12.5mg + amlodipine 5mg) by means of a clinical study in a population of hypertensive patients, in which the primary objective will be to evaluate the efficacy through non-inferiority of the above-mentioned combination compared to Exforge HCT® (valsartan 160mg + hydrochlorothiazide 12.5mg + amlodipine 5mg) in the average change in systolic blood pressure 12 weeks after treatment initiation compared to baseline.
In this way, we believe that conducting the clinical study will provide therapeutic benefit to the study population and could corroborate evidence suggesting that the combination of angiotensin II receptor blocker + thiazide diuretic + calcium channel blocker is an appropriate option for treating arterial hypertension.

OBJECTIVES

PRIMARY OBJECTIVE

To evaluate the efficacy through the non-inferiority of the combination of candesartan cilexetil 16 mg + chlorthalidone 12.5 mg + amlodipine 5 mg compared to Exforge HCT® (valsartan 160mg + hydrochlorothiazide 12.5mg + amlodipine 5mg) in the mean change in SBP 12 weeks after starting treatment compared to baseline.

The primary efficacy outcome is the mean change in SBP, to be measured at the research center 12 weeks (± 4 days) after the initiation of treatment, compared to baseline (V0).

SECONDARY OBJECTIVES

To evaluate the change in DBP 4, 8 and 12 weeks after the initiation of treatment; To evaluate the change in SBP 4, 8 weeks after the initiation of treatment; To evaluate the proportion of participants achieving target blood pressure (SBP <140 and DBP <90 mmHg) 4, 8 and 12 weeks after initiation of treatment; To evaluate the proportion of participants achieving the target blood pressure of SBP <120 mmHg 4, 8 and 12 weeks after initiation of treatment; To evaluate the proportion of participants achieving target SBP <140 mmHg 4, 8 and 12 weeks after starting treatment; To evaluate the proportion of participants achieving the target DBP<90 mmHg 4, 8 and 12 weeks after initiation of treatment; To evaluate the proportion of participants who demonstrate a reduction of greater than or equal to 20 mmHg in SBP 4, 8 and 12 weeks after initiation of treatment; To evaluate the proportion of participants demonstrating a reduction greater than or equal to 10 mmHg in DBP 4, 8 and 12 weeks after initiation of treatment. To evaluate treatment safety based on the occurrence of adverse events and changes in laboratory tests results, clinical/physical evaluations and vital signs.

The study's secondary efficacy outcomes were defined as:

Mean change in DBP, measured at the research center, at 4, 8 and 12 weeks (± 4 days) after the start of treatment, compared to baseline; Mean change in SBP, measured at the research site, established at 4 and 8 weeks (± 4 days) after the initiation of treatment, compared to baseline (V0); Proportion of participants who, after initiation of treatment, achieve target blood pressure (SBP <140 and DBP <90 mmHg) at 4, 8 and 12 weeks (± 4 days); Proportion of participants who, after initiation of treatment, achieve target SBP <120 mmHg at 4, 8 and 12 weeks (± 4 days); Proportion of participants who, after initiation of treatment, achieve target SBP <140 mmHg at 4, 8 and 12 weeks (± 4 days); Proportion of participants who, after initiation of treatment, achieve the target DBP<90 mmHg at 4, 8 and 12 weeks (± 4 days); Proportion of participants who, after initiation of treatment, show a reduction of greater than or equal to 20 mmHg in systolic blood pressure at 4, 8 and 12 weeks (± 4 days); Proportion of participants who, after initiation of treatment, show a reduction of greater than or equal to 10 mmHg in diastolic blood pressure at 4, 8 and 12 weeks (± 4 days). The Incidence of adverse events (AEs) recorded from the first dose of PSI until 30 days after the end of the treatment provided for in the protocol, however all all adverse events occurring after the consent obtained through the ICF should be collected and reported; Proportion of participants with changes in laboratory tests results, considered clinically relevant according to the investigator's criteria, at the screening visit (V-1) and final visit (FV) of the study**.** Proportion of participants with clinical or physical changes, considered clinically relevant according to the investigator's criteria, at visits V1, V2 and FV compared to baseline (V0); Absolute change in vital signs measured at visits V1, V2 and FV compared to the baseline visit (V0).

Note: Although only adverse events occurring after the use of the investigational/comparator product will be used in the analysis that will evaluate the safety of the treatments, all adverse events occurring after the consent is obtained through the ICF will be collected and reported.

ELIGIBILITY CRITERIA FOR STUDY POPULATION

STUDY POPULATION

Participants of both sexes, aged 18 years or older, who present with SBP measurements ≥ 140 mmHg and ≤180 mmHg and DBP ≥ 90 mmHg and ≤ 110 mmHg despite the use of dual antihypertensive therapy from different therapeutic classes for at least 8 week.

NUMBER OF PARTICIPANTS

A total of 698 study participants who meet all the inclusion criteria and do not meet any of the exclusion criteria will be randomized to one of the two study treatment groups.

INCLUSION CRITERIA

Men and women aged 18 years or older;
Patients currently using dual antihypertensive therapy, from different therapeutic classes, for at least 8 weeks and non-responders to this treatment, defined as office measurements of SBP ≥ 140 mmHg and ≤180 mmHg and DBP ≥ 90 mmHg and ≤ 110 mmHg evaluated at the screening and randomization visits (both conditions are in accordance with the Brazilian Hypertension Guideline - 2020);
Ability to understand and provide consent to participate in this clinical study, as documented by signing the Informed Consent Form (ICF).

EXCLUSION CRITERIA

Any clinical observation finding (clinical/physical evaluation and vital signs) that is interpreted by the investigator as a risk to the research participant;
Any laboratory test finding that the investigator considers to be a risk to the research participant;
Participants suspected or diagnosed with COVID-19;
Known hypersensitivity to any components of the study medications or to agents derived from sulphonamides;
Women who are pregnant or breastfeeding;
Menopausal women who do not agree to use effective contraceptive methods [oral contraceptive, injectable contraceptive, intrauterine device, hormonal implant, barrier methods, hormonal transdermal patch, vaginal ring and tubal ligation]; except those who are surgically sterile (bilateral oophorectomy or hysterectomy), those who have been menopausal for at least one (01) year and participants who declare that they are abstinent or engage exclusively in non-reproductive sexual practices; Male participants who do not agree to use effective contraceptive methods: contraceptive methods for the participant: barrier condoms, except for those who are surgically sterile (vasectomy) or for participants who are sexually abstinent during the study period or participants who declare that they engage exclusively in non-reproductive sexual practices; OR contraceptive methods for the female partner: oral contraceptive, injectable contraceptive, intrauterine device, hormonal implant, hormonal transdermal patch, tubal ligation, vaginal ring and barrier methods except for female partners who are surgically sterile (bilateral oophorectomy or hysterectomy), menopausal for at least one (1) year;
Research participants who have participated in clinical study protocols within the past 12 (twelve) months (CNS Resolution 251, of August 7, 1997, item III, sub-item J), unless the investigator. believes that the participation may offer a direct benefit to the individual;
Participants who have a relationship of up to the second degree or are related to collaborators or employees of the Sponsor and the Research Site; P
articipants with an estimated glomerular filtration rate (eGFR) of less than 45 ml/min /1.73m2 (calculated using the Chronic Kidney Disease Epidemiology Collaboration [CKD-EPI] equation) or end-stage kidney disease;
Participants with a history of severe liver dysfunction;
Participants with a history of cardiogenic shock or heart failure with reduced ejection fraction, with left ventricular ejection fraction less than or equal to 50%;
History of symptomatic congestive heart failure classes II, III or IV according to the New York Heart Association (Annex I) and/or participants with a history of infarction, unstable angina or stroke in the past 6 months prior to the initiation of the study;
History of clinically relevant ventricular cardiac arrhythmias;
Participants with a history of obstructive coronary artery disease undergoing percutaneous or surgical coronary intervention;
Participants with a history of dementia syndrome;
History of alcohol or illicit drug dependence in the six months prior to the date of consent obtained through the ICF;
Participants who use prohibited medication, as described in item 9.1";
Participants with a history of obstructive biliary disorders;
Participants with a history of refractory hypokalemia and/or conditions involving marked loss of potassium, hyperkalemia (with serum potassium levels above 5.5 mmol/L) and/or hyponatremia;
Participants with a history of symptomatic hyperuricemia (history of gout or uric acid calculation);
Participants with a history of secondary arterial hypertension;
Participants with a medical history of malignant neoplasia, without documentation of remission/cure.

Libbs

CLINICAL STUDY

RATIONALE FOR THE CLINICAL STUDY

Arterial hypertension is one of the leading causes of death in the world and is responsible for approximately 10.4 million deaths per year worldwide (UNGER, 2020). In Brazil, from 2008 to 2017 there were an estimated 667,184 deaths from hypertension or its complications (MORAGA, 2017; BARROSO, 2020; WHO, 2008). Fifty percent of hypertensive patients have other cardiovascular risk factors, which, together with hypertension, considerably increase the risk of coronary, cerebrovascular and kidney diseases in these patients (UNGER, 2020).

Cardiovascular (CV) protection is one of the main objectives of antihypertensive treatment. Reducing blood pressure (BP) is the primary goal, with the overarching objective of reducing CV outcomes and mortality associated with hypertension (AH). The results of meta-analyses of randomized clinical trials in hypertensive patients have shown that reducing systolic BP by 10mmHg and diastolic BP by 5mmHg with medication is accompanied by a significant reduction in the relative risk of major outcomes: 37% for cerebrovascular accident (CVA), 22% for coronary artery disease (CAD), 46% for heart failure (HF), 20% for CV mortality and 12% for total mortality (BARROSO, 2020).

Considering that drug treatment for arterial hypertension may be initiated with monotherapy, but 58% of hypertensive patients require combinations of 2 or 3 antihypertensives with different mechanisms of action to achieve the blood pressure target (ANVISA, 2010), research is necessary to evaluate the effectiveness of new fixed-dose drug combinations in order to facilitate and promote treatment adherence. Furthermore, in the specific case of antihypertensives, the antagonism of some drugs to the adverse events of agents from other pharmacological classes has already been demonstrated, significantly reducing the adverse events demonstrated by patients (ANVISA, 2010).

According to the Brazilian Hypertension Guideline (2020), combinations of three drugs are indicated for those who have not achieved the blood pressure target with treatment with a combination of two drugs. Therefore, a triple combination was defined as a comparator in the clinical study, since the target populations of treatments with double and triple combinations differ from each other.

At the time this project was conceived, there was only one triple combination available on the market that had the same therapeutic classes/mechanism of action as the combination under study, which was composed of valsartan + hydrochlorothiazide + amlodipine.
Some studies have demonstrated significant results in reducing blood pressure provided by candesartan cilexetil compared to valsartan (NAKAJIMA, 2020) and chlorthalidone compared to hydrochlorothiazide, and are even show superior efficacy in preventing cardiovascular events (LIANG, 2017; ROUSH, 2012).

The combination of candesartan cilexetil and amlodipine besylate has already proven effective in reducing DBP and SBP and safe compared to monotherapy of both drugs (SOHN, 2017; LEE, 2019). Considering the great social impact, the quality of life of patients and the reduction in serious complications resulting from arterial hypertension, effective pharmacological options are extremely important for the therapeutic arsenal. Given this situation, the present study proposes a fixed-dose combination consisting of candesartan cilexetil 16 mg + chlorthalidone 12.5 mg + amlodipine 5 mg, with the objective of providing therapeutic efficacy by reducing systolic and diastolic blood pressure. The proposed combination contains candesartan cilexetil, an angiotensin II receptor blocker; chlorthalidone, a thiazide diuretic; and amlodipine, a calcium channel blocker. All these medications are widely used in the treatment of hypertension and the aforementioned pharmacological classes are the main ones indicated by the Brazilian Hypertension Guidelines (BARROSO, 2020).

The combination of different classes of medications seeks to improve their antihypertensive potential as well as reduce adverse events and thus increase treatment adherence. Additionally, the combination of drugs with distinct mechanisms of action allows the use of lower doses of each compound, thus avoiding the need to use maximum doses for each pharmacological agent. Thus, the present phase III, multicenter, randomized, double-blind, parallel, controlled clinical study with Exforge HCT® (valsartan 160mg + hydrochlorothiazide 12.5mg + amlodipine 5mg) was outlined, which aims to evaluate the non-inferiority of the experimental medication in research participants aged 18 years or older and who, even on dual use of antihypertensive therapy for at least 8 weeks, have not achieved target blood pressure levels.

Participants who have used dual therapy, from different therapeutic classes, for at least 8 weeks, but who still have not achieved the target blood pressure levels and who have SBP measurements ≥ 140 mmHg and ≤180 mmHg and DBP ≥ 90 mmHg and ≤ 110 mmHg will be classified as non-responders. Furthermore, to ensure that the participant's blood pressure is stabilized, two blood pressure measurements will be taken in the office using a calibrated and validated

digital blood pressure measuring device, one measurement at the screening visit and the other at the randomization visit (before randomization), following the guidance of the Brazilian Hypertension Guideline (2020), which recommends that the diagnosis of arterial hypertension should always be validated by repeated measurements, under ideal conditions, in two or more medical visits at intervals of days or weeks (Hypertension Guideline 2020).

This study will not include the *run-in*/*washout* period, as this is a population that is unresponsive to previous treatment, with a double antihypertensive combination requiring immediate medication to maintain blood pressure within normal parameters, since sustained elevations in blood pressure correlate with an increased risk of adverse cardiovascular events. Furthermore, according to the 2020 Brazilian Hypertension Guideline, the addition of a new drug is conducted without the need for prior *washout*. Furthermore, considering the package inserts of individual medications, some antihypertensive classes cannot be used concomitantly, so the prohibition of the use of other antihypertensive classes was inserted in the item of prohibited medications only after randomization to treatment. Thus, the design of the present study promotes an improvement in external validity, contributing to a greater possibility of generalizing the results to the Brazilian population.

Meta-analysis data including studies evaluating the impact of introducing pharmacotherapy for treating hypertension suggest that approximately 50% of the maximum effect of antihypertensives in reducing systolic blood pressure occurs after the first week of treatment, with a progressive reduction in the effect in subsequent weeks, reaching a certain stability after 4 weeks (LASSERSON, 2011). Data from several randomized clinical studies confirm the stabilization of pressure values after 4 to 12 weeks. (AGARWAL, 2021; CHOW, 2021; MACDONALD, 2017). Therefore, the recommended treatment time for this study will be 12 weeks, as it will be possible to evaluate adverse events linked to the medications and, if the blood pressure target is not achieved, the participant will not remain without effective treatment for a long period (LEE, 2019; Hypertension Guideline 2020).

DURATION OF CLINICAL STUDY

The study will last up to 136 days, considering a screening visit (V-1; +4 days), with a treatment period of 12 weeks, including 01 Randomization Visit (V0;), 02 Follow-up Visits (V1 and V2; ± 4 days) and a Final Visit (FV; ± 4 days). A telephone contact will be made 30 days (+7) after the end of the treatment provided for in the protocol.

CLINICAL STUDY DESIGN

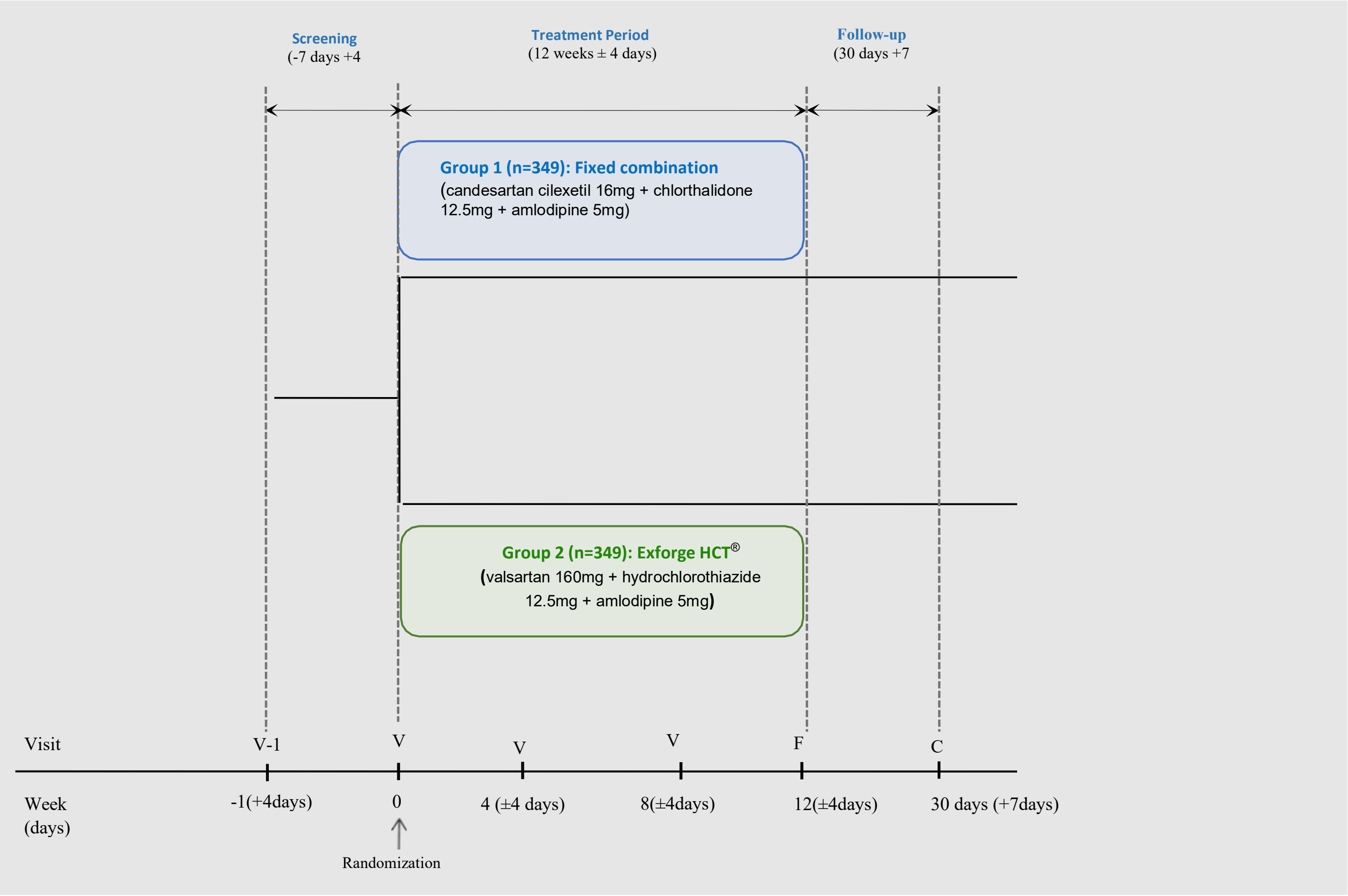

Figure 6**:** Illustration of clinical study design

STUDY PLAN

The study aims to evaluate the efficacy and safety of the experimental drug, a triple combination of candesartan cilexetil 16mg + chlorthalidone 12.5mg + amlodipine 5mg compared to the comparator Exforge HCT® (valsartan 160mg + hydrochlorothiazide 12.5mg + amlodipine 5mg) in the treatment of systemic arterial hypertension.

Women and men aged 18 years or older who, even if currently using dual antihypertensive therapy from different therapeutic classes, have maintained SBP ≥ 140 mmHg and ≤180 mmHg and DBP ≥ 90 mmHg and ≤ 110 mmHg measured in the office using a digital blood pressure measuring device calibrated, validated and approved by the Brazilian Society of Hypertension for at least 8 weeks will be identified for participation in the clinical study protocol. The selection of the research participant will occur after the consent of the research participant obtained through the ICF, which must be applied before any procedure related to the study. All study procedures must be explained to the research participant, and any questions they may have must be answered by the investigator assigned to conduct the study at the research site or by the person responsible for such activity and recorded on the activity delegation form.
The study includes a Screening Visit (V-1), a Randomization Visit (V0), two Follow-up Visits (V1 and V2), a Final Visit (FV) and a telephone contact (CT).

The Screening visit (V-1) will be the first visit of the study, and all procedures should only be performed after the participant's consent has been obtained through the ICF. The purpose of this visit is to verify the eligibility criteria that may be evaluated at that time. The Randomization Visit (V0) will occur one week (+ 4 days) after the Screening Visit (V-1). At this visit, all eligibility criteria must be verified and, if the participant is eligible, he/she must be randomized and allocated to one of two treatment groups:

Group 1:
Candesartan cilexetil 16mg + chlorthalidone 12.5mg + amlodipine 5mg;

Group 2:
Exforge HCT®(valsartan 160mg + hydrochlorothiazide 12.5mg + amlodipine 5mg)

Follow-up Visits (V1 and V2) will occur four and eight weeks (± 4 days) respectively after the date of the Randomization Visit (V0). The purpose of this visit is to evaluate the efficacy and safety of the Investigational Product over the course of the study. During this visit, laboratory tests will be performed to monitor possible kidney and/or electrolyte changes.

The Final Visit (FV) will take place twelve weeks (± 4 days) after the date of the Randomization Visit (V0). This visit aims to evaluate the effectiveness and safety of PSI after the end of treatment. This visit will be the final in-person visit of the study, therefore, the research participant must be informed about the study conclusion.

Telephone Contact (CT) will be made 30 (+7) days after the end of treatment with PSI. The purpose of this contact is to evaluate the possible occurrence of adverse events during this period.

Research participants who do not meet any of the study's inclusion criteria and/or meet any of the exclusion criteria will be considered screen failure. They may be reevaluated (re-screened) at a later time, as long as inclusion is open. Each participant may undergo a re-screening only once. The research participant who is re-screened will receive a new identification number.

The results of examinations performed at all visits should be evaluated by the investigator or sub-investigator as soon as they are available. In the event of a clinically relevant abnormality, an Uneschedule Visit (UV) may be scheduled, if the investigator/sub-investigator deems it necessary for guidance, treatment and evaluation of discontinuation criteria.

If the research participant is prematurely discontinued from the study, he/she must complete the Final Study Visit (FV).
Participants may be replaced when the number of participants who discontinue the study or who have had their treatment permanently interrupted is higher than the previously established *dropout* rate. However, all efforts and training will be taken with the sites to ensure adherence to the protocol.

ASSESSMENTS

The assessments described below must be conducted in accordance with the study schedule, during regulatory and uneschedule visits, when applicable, so that there is accurate data collection, improving the process of following-up the clinical assessments of research participants. If any assessment does not correspond to what is accepted as normal and is clinically relevant, this must be related to the participant's medical history or must be documented as an adverse event.

The term “non-clinically relevant abnormal” (NCR Abnormal) used in this study refers to situations in which the change is minimal and does not have an impact on the health or safety of the participant, such as, for example, when the result of a laboratory test falls slightly outside the reference range, but the investigator considers that the change is not significant. If a change in a laboratory test is recorded in the e-CRF that does not qualify as “non-clinically relevant abnormal”, as per the rationale described above, it must be related to a previously recorded adverse event/medical history or a new adverse event must be reported in the e-CRF. If necessary, an Uneschedule Visit (UV) may be scheduled, at the discretion of the investigator for guidance, treatment and evaluation of discontinuation criteria.

CLINICAL AND PHYSICAL ASSESSMENT

Baseline Medical HistoryThe Investigator must perform, on the first study visit, an baseline medical history to ascertain the history of the current clinical condition and medical history of the research participant, such as, but not limited to: history of cardiovascular diseases (atrial fibrillation, left ventricular hypertrophy, etc.), respiratory diseases, kidney, urological, endocrine,neurological diseases, etc. The medical history of family antecedents related to the study (father, mother and siblings) must also be verified, such as: early coronary disease or hypertension. The participant must be evaluated regarding contraceptive methods, as well as previous treatments, surgical procedures and medications. Only those medications/treatments whose administration began before the start of treatment (V0) should be considered as prior medications/treatments, and these are subdivided into: discontinued previous medications (whose last dose was administered before V0) and ongoing previous medications (medications that were maintained after V0). If the medication/treatment in question is initiated on the day of the start of treatment or after that date, limited to the date of the participant's last visit to the study, they will be considered concomitant medications. Social history regarding smoking (classified as: smoker, former smoker and never smoked) and alcohol consumption (number of doses of alcohol consumed on average per week) should also be obtained.

CLINICAL ASSESSMENT

The Investigator must perform a clinical assessment to determine the current clinical condition, concomitant pathologies, evaluation of contraceptive methods, as well as treatments, surgical procedures and/or concomitant medications.

General Physical Assessment : The Investigator must perform the physical examination, considering the following parameters: Weight (kg); Height (m) (only on the first visit); Body Mass Index (BMI); Respiratory System; Cardiovascular System. Vital Signs The Investigator must check vital signs: Blood pressure in a sitting position; Heart Rate (HR); Respiratory Rate (FR).

Blood pressure and heart rate will be evaluated after the research participant has rested for at leat 5 minutes in a calm environment. Before the measurement, it must be certified that the participant does not have a full bladder, has not exercised in the last 60 minutes, has not consumed alcoholic beverages, coffee or food in the last 120 minutes and has not smoked in the last 30 minutes. The procedure must be explained to the research participant and he or she must be instructed not to talk during the measurement. Any questions you may have should be clarified before or after the procedure.

The participant must be in a seated position, with legs uncrossed, feet resting on the floor, back leaning back in the chair and relaxed. The arm should be at heart level, supported, with the palm facing upwards, and clothing should not strangle the limb. On all visits, measurements must be conducted by the same delegated team, trained and qualified for such activity and whenever possible at the same time of day, using a validated and calibrated blood pressure device. All research sites included in the study will use device of the same model HEM 7122 provided by the sponsor. It must be described in the source document that the device provided by the sponsor was used.

Regarding heart rate, the last reading obtained must be recorded in the source document and electronic clinical record.
In the first measurement of the first visit, blood pressure measurements should be obtained in both arms, preferably simultaneously, to detect possible differences between the arms and, in the event of a difference, the arm with the highest systolic BP value should be used as a reference for subsequent measurements and visits.

In total, three measurements should be taken, with an interval of around 1 to 2 minutes between measurements. However, additional measurements should be taken if there is a discrepancy of more than 10 mmHg in systolic blood pressure between previous measurements. Additional readings should be obtained until stabilization occurs. The BP must be recorded, but it will be the average of the last two readings that must be considered for evaluating the eligibility criteria. The table below shows the classification of blood pressure according to casual measurements in the office for subjects aged 18 years or older (table 2).

Table: Blood pressure classification according to casual or office measurement (≥18 years)
Source: Brazilian Society of Cardiology - Brazilian Guidelines for Arterial Hypertension 2020)

Classification*	SBP (mHg)		DBP (mHg)
**Optimal BP**	< 120	and	< 80
**Normal BP**	120 - 129	and/or	80 - 84
**Pre-hypertension**	130 - 139	and/or	85 - 89
**AH Stage 1**	140 - 159	and/or	90 - 99
**AH Stage 2**	160 - 179	and/or	100 - 109
**AH Stage 3**	≥ 180	and/or	≥ 110

*AH: arterial hypertension; BP: blood pressure; SBP: systolic blood pressure; DBP: diastolic blood pressure. *Classification i s d efined according to office BP and the highest level of BP, systolic or diastolic. **Isolated systolic AH, characterized by SBP ≥ 140 mm Hg and DBP < 90 mmHg, is classified as 1, 2 or 3, according to SBP values in the indicated ranges. ***Isolated diastolic AH, characteriz ed by SBP < 140 mmHg and DBP ≥ 90 mmHg, is classified as 1, 2 or 3, according to the DBP values in the indicated ranges.*

Therefore, the following normal values will be considered for research participants aged 18 or over: Systolic Blood Pressure (SBP) ≤129 mmHg; Diastolic Blood Pressure (DBP) ≤84 mmHg.

LABORATORY TESTS

The laboratory profile of research participants will consist of the following tests, depending on the visit, and must be evaluated by the investigator or sub-investigator:

Visit V-1 and FV:
Complete blood count, including platelet count, random blood glucose, glycated hemoglobin, serum sodium, serum potassium, uric acid, glutamic oxaloacetic transaminase (TGO), glutamic pyruvic transaminase (TGP), alkaline phosphatase, total bilirubin and fractions, serum creatinine, urea, total cholesterol and fractions (lipid profile)*, total proteins and fractions, creatine phosphokinase (CPK), thyroid-stimulating hormone (TSH) and blood BhCG (only for women of childbearing potential);
Urine type I.

Visit V1 and V2:
Serum creatinine, serum sodium, serum potassium, urea and blood BhCG (only for women of childbearing potential).
(*) When the TG (triglycerides) value is above 400mg/dL, the research site whose laboratory does not issue the LDL (*Low Density Lipoprotein*) result must calculate the LDL using the Martin formula, 2013 (LDL = CT - HDL - (Trig/NF), as described in the annex IV. A note to the file should preferably be prepared by the laboratory or, if this is not possible, by the research site and filed in the investigator's folder.

At these visits (V-1, V1, V2 and FV) the estimated glomerular filtration rate (eGFR) should be calculated using the *Chronic Kidney Disease Epidemiology Collaboration* [CKD-EPI]) equation. This value will be calculated by the eCRF, however the value found must be described in the source document.

Laboratory tests and identification of sample collection material must be performed in accordance with the Laboratory Procedures Manual available for consultation at the research site, if applicable. The necessary preparations for collecting the tests must be respected in accordance with the Procedures Manual, if applicable. The collection will be performed by a trained and authorized professional, using sterile and disposable materials. Collection tubes must be identified in order to maintain traceability of the research participant. There will be no fasting period for the test, the collection must be collected casually, the blood sample must be collected through the participant's arm. All blood samples from participants will be discarded after analysis by the laboratory. Blood biological samples will only be used for the purpose of evaluating the safety of this clinical study. Therefore, they will not be used for any purpose other than those described. The results of laboratory tests performed at all visits should be evaluated by the investigator or sub-investigator as soon as they become available.

ADVERSE EVENT ASSESSMENT

All adverse events occurring during the clinical study after informed consent has been obtained through the ICF, must be documented in both source document and Clinical Record (e-CRF) related to the research participant, characterizing them in terms of severity, intensity and relationship with the investigational product. The action taken regarding the use of the medications under study and the progression of the adverse event must also be recorded, as described in the Clinical Protocol.
Note: Although all adverse events occurring after informed consent has been obtained through the Informed Consent Form (ICF) must be collected and reported, only adverse events occurring after the use of the investigational product will be used in the analysis that will evaluate the safety of the treatments.

TREATMENT ADHERENCE ASSESSMENT

At all visits after the randomization visit (V0), the research participant will be asked to return the blister packs of the study medications in order to verify their adherence to the established treatment. The following formula should be used for calculation:

(Expected amount of use) ----------100%
(Amount of use performed**) --------X

*The value of X refers to the percentage of adherence to treatment.

**In all visits, after the Randomization visit (V0), the research participant will be asked to return the IP packaging, in order to verify their adherence to the established treatment. Adherence assessment will be performed by checking the tablets returned in their respective packaging, as per the guidance provided by the delegated professional at the time of dispensing, and also through the report of the research participant. In the event of discrepancies in information between the quantity of medication returned and the quantity reported by the research participant, the investigator or the person(s) responsible for the information delegated by him/her must consider the following premises for this assessment:

In situations where the research participant reports having used a greater quantity than that found through the accounting of the returned experimental/comparator/placebo medication tablets, the accounting of the tablets must be considered mandatory; In cases where the research participant reports having used a smaller quantity of the experimental/comparator/placebo medication than that found through accounting, the participant's report with due justification for the absence of these tablets must be considered mandatory; In cases where the research participant reports the absence of the experimental/comparator/placebo medication packaging at the time of the visit, every effort must be made to retrieve this packaging. However, if return is not possible due to loss, the participant's report will be considered mandatory; In other situations, the investigator must conduct an evaluation of each case, recording it in a source document; The Investigator or the person(s) responsible for the Investigator must account for the PSI, recording the data and possible discrepancies in the source document.

PROCEDURES DURING CLINICAL STUDY VISITS

Any study procedure may only be conducted after the research participant has been informed of his/her participation in the clinical study and expressed his/her consent through the respective ICF.
All procedures related to study visits must be conducted by qualified and duly delegated persons by the Principal Investigator and documented in the List of Delegation of Responsibilities and made available to the Monitor. The procedures must be documented immediately after they are conducted, in a source document duly signed, stamped and dated by the Investigator. Transcription for the CRF should preferably be completed within five working days. The record of all actions relating to the clinical study protocol must be made in the research participant's document.

The processes described in the protocol must be followed within the defined intervals for study visits. If the investigator believes that it is necessary to eliminate an imminent risk to the research participant or if unexpected circumstances arise that require deviation from specific protocol procedures, the investigator should consult with the sponsor/CRO to determine the appropriate action. Any action that falls outside the procedures described in the protocol, such as, but not limited to, conducting visits outside the window, not perform visit tests, failure to dispense the PSI, will be considered a protocol deviation and must be communicated to the Ethics Committee (EC) and the Sponsor. Any action that falls outside the procedures described in the protocol and is considered a serious deviation and may affect the quality of the data, compromise the integrity of the study, affect the safety or rights of the study participants, such as, but not limited to, the incorrect assessment of the eligibility criteria and dispensing of the incorrect medication for the treatment group, will be considered a violation of the protocol, and must be communicated to the Ethics Committee (EC) and the Sponsor/CRO.

1. SCREENING VISIT – V(-1) (DAY -7 (+4)/WEEK - 1

The procedures to be conducted are: Informed Consent process through the ICF; Baseline medical history, as per item 5.1.1; Clinical evaluation, as per item 5.1.2; General physical examination, as per item 5.1.3; Verification of vital signs, as per item 5.1.4; Evaluation of the use of previous medications and treatments; Verification of inclusion and exclusion criteria that may be evaluated during this visit, as per items 3.3 and 3.4; Collection of laboratory tests, as per item 5.2; Test results must be available for evaluation at V0. Provision of male barrier condoms and guidance to research participants on the use of contraceptive methods throughout the study, if applicable; Evaluation of the occurrence of adverse events occurring after consent has been obtained through the ICF; Scheduling the Randomization visit [V0; 07 days (+4) after V(-1)] and instructing the participant not to use their usual antihypertensive medication on the day of V0.

2. RANDOMIZATION VISIT – V0 (WEEK 0)

The procedures to be conducted are: Clinical evaluation, as per item 5.1.2; General physical examination, as per item 5.1.3; Verification of vital signs, as per item 5.1.4; Verification of inclusion and exclusion criteria, as per items 3.3 and 3.4; Performing randomization and allocating participants to one of the treatment groups, according to the randomization (if the research participant does not meet any of the inclusion criteria and/or meets any exclusion criteria, he/she will not be randomized and the investigator must consider it as a screen failure); Dispensing of IP and guidance to the participant on its correct use (preferably the first dose should be administered at the site under the guidance of the delegated team, however, if the participant has used the usual antihypertensive medication on the same date, he/she should be advised to use the IP the following day, in the morning); Evaluation of the use of previous/concomitant/prohibited medications and treatments used since the last visit; Dispensing of the package leaflet regarding guidance on the use of IP to the research participant (annex III); Provision of male barrier condoms and guidance to research participants on the use of contraceptive methods throughout the study, if applicable; Guidance to the participant regarding the interruption of the usual antihypertensive medication treatment and regarding prohibited medications, described in item 9.1; Evaluation of the occurrence of adverse events that have occurred since the last visit; Assessment of discontinuation criteria, as per items 6.7 and 6.8; Scheduling of follow-up visit (V1) 04 weeks ± 4 days after V0.

3. FOLLOW-UP VISITS – V1 and V2 (WEEK 4 and WEEK 8 ± 4 days)

The procedures to be conducted are: Clinical evaluation, as per item 5.1.2; General physical examination, as per item 5.1.3; Verification of vital signs, as per item 5.1.4; Collection of laboratory tests, as per item 5.2; Evaluation of the use of previous/concomitant/prohibited medications and treatments used since the last visit; Collect the packages of IP dispensed during the previous visit, as well as all unused medication, and conduct an accounting of these and a treatment adherence assessment; Dispensing of IP and guiding the participant on its correct use. The package leaflet must be given to the research participant with instructions on how to use the IP (annex III); Provision of male barrier condoms and guidance to research participants on the use of contraceptive methods throughout the study, if applicable; Assessment of the occurrence of adverse events that have occurred since the last visit; Evaluation of discontinuation criteria, as per items 6.7 and 6.8; At V1, schedule Follow-up Visit 2 (V2) 8 (± 4 days) weeks from V0. At V2, schedule the Final Visit (FV) 12 (± 4 days) weeks from V0.

4. FINAL VISIT – FV WEEK 12 (± 4 days)

The procedures to be conducted are: Clinical evaluation, as per item 5.1.2; General physical examnination, as per item 5.1.3; Verification of vital signs, as per item 5.1.4; Evaluation of the use of previous/concomitant/prohibited medications and treatments used since the last visit; Performing laboratory test collection, as per item 5.2; Collect the study medication (IP) packages dispensed during the previous visit, as well as all unused medication, and conducting accounting of these as well as evaluate adherence to treatment; Provision of male barrier condoms and guidance to research participants on the use of contraceptive methods until the time of telephone contact, if applicable; Assessment of the occurrence of adverse events that have occurred since the last visit; Guiding the research participant regarding the conclusion of their participation in the clinical study and referring them to the outpatient clinic for medical follow-up; Scheduling of telephone contact 30 (+7) days after the end of treatment.

5. TELEPHONE CONTACT (CT)

Telephone contact will be made (CT). The research participant will also be monitored through CT, which must be conducted 30 (+7) days after the end of the treatment provided for in the protocol. The purpose of the contact is to check for adverse events and general health status. If the participant is prematurely and permanently discontinued from treatment due to discontinuation criteria d (from item 6.8), the CT must be performed 30 (+7) days after the interruption of treatment with the study medications.

6. UNESCHEDULED VISIT (UV)

An unescheduled visit must be conducted if the research participant presents any adverse event, clinically relevant changes in signs, symptoms or laboratory tests during the study period, for guidance, treatment and evaluation of discontinuation criteria, at the discretion of the Investigator. This visit must be numbered after the last regular visit, recorded in the source document and in the study's clinical record. For example, an unescheduled visit after Visit 1 will be numbered as Visit 1.1 and so on until Visit 2 is performed. If the unescheduled visit results in the research participant leaving the clinical protocol or discontinuing the use of the PSI, the FV must be performed.

7. TEMPORARY OR PERMANENT INTERRUPTION OF THE INVESTIGATIONAL PRODUCT

In cases where any of the situations below occur, the research participant must interrupt the treatment, but will be instructed to attend all regular study visits to complete the safety procedures outlined in the protocol, if the participant agrees. The interruption of the IP does not, therefore, constitute the withdrawal of the participant from the clinical study.

IP should be permanently discontinued in the following situations: Occurrence of pregnancy during the conduct of the clinical study; Use of prohibited treatments/medications as detailed in item 9.1; Violation of the protocol that, in the opinion of the investigator, affects the safety of the research participant; Any finding, clinical condition, laboratory test or adverse event that, at the discretion of the investigator, precludes the research participant from continuing treatment, describing the reason, with respective proof; Diagnosis of disease or treatment during the study that is part of the exclusion criteria.

8. PARTICIPANT DISCONTINUATION FROM THE STUDY

In cases where research participants present any reason for discontinuing the study protocol, Libbs Pharmaceuticals/CRO must be informed by the investigator. In these cases, the FV must be conducted, covering all the procedures foreseen for this visit. The criteria for discontinuation of a clinical study research participant are: Withdrawal of Consent: research participants who, for any reason, withdraw their informed consent; Abandonment of the protocol/loss of follow-up by the

research participant; Death of research participant; Violation of protocol that in the opinion of the investigator affects the safety of the participant.

In cases where the research participant is lost to follow-up, defined as "the research participant does not return on the scheduled date", the research site must make telephone contact within a maximum period of one (1) business day after the scheduled visit. The procedure must be documented in a source document and the new visit scheduled, complying with the maximum period of five (5) working days.
In situations where telephone contact is not effective, two (2) more attempts must be made. If these are also not effective, a telegram, WhatsApp or text message must be sent to the research participant requesting that they contact them urgently. If there is no response within 3 days, the research participant will be classified as having abandoned the research. Attempts at telephone contact as well as confirmations of receipt of telegrams must be recorded/attached to the research participant's source document.

MATERIALS AND METHODS

TREATMENT GROUP

Treatments will be assigned to research participants in a 1:1 ratio, based on the code generated in the randomization list.

RESEARCH PARTICIPANT IDENTIFICATION

Research participants will have their identity preserved and will be identified by letters corresponding to their first, second and last initial, including "junior", "son" and "grandson". For example: José Marques da Silva Júnior (JMJ). If the participant has only two components in their name, only the two initials will be considered. For example: Maria de Souza (MS).

In addition to identification by initials, research participants will also be identified by a code. At the screening visit, after consent has been obtained through the ICF, each research participant will be identified by the study code (LB2009), followed by two digits defined sequentially by the sponsor, referring to the order of inclusion of the research site in the study and by a sequential four-digit numeric code corresponding to the order of inclusion of the participant in each center. For example: the first person screened from research site 01 will be identified by "LB*2009* 010001", the second person screened from the same site will be identified by "*LB2009* 010002", the first person screened from research site 03 will receive the code "*LB2009* 030001", and so on. After the research participant is randomized, he/she will continue to have the same identification number, corresponding to the randomization. This identification must be applied to all documentation of research participants (medical records, correspondence) as well as to IP cartridges (boxes) and other study elements for which participant identification is necessary.

STUDY MEDICATIONS

DESCRIPTION OF THE INVESTIGATIONAL PRODUCT

Generic name: fixed combination of candesartan cilexetil, chlorthalidone and amlodipine; Manufacturer: Libbs Pharmaceuticals Pharmaceutical form: tablet; Concentration: candesartan cilexetil 16mg + chlorthalidone 12.5mg + amlodipine 5mg; Form: at each visit (V0, V1 and V2) 2 blue blister packs will be dispensed, both containing 30 tablets of the active experimental drug or placebo and 2 white blister packs both containing 30 tablets of the active comparator drug or placebo; Dosage: administer one (1) tablet of the active experimental drug (blue blister pack) and then one (1) tablet of the placebo of the comparator drug (white blister pack), orally, once a day, in the morning; If the research participant forgets to take a dose of the medication, it should be administered as soon as he/she remembers and then the next dose should be administered at the usual time. However, if it is close to the time for the next dose, the participant should not take the missed dose, and the minimum interval of 6 hours between doses must be respected. The participant should not take a double dose to make up for a missed dose.

COMPARATOR PRODUCT DESCRIPTION

Generic name: valsartan, hydrochlorothiazide and amlodipine; Brand: Exforge HCT; Manufacturer: Novartis Biociências SA Pharmaceutical form: coated tablet; Concentration: valsartan 160mg + hydrochlorothiazide 12.5mg + amlodipine 5mg Form: at each visit (V0, V1 and V2) 2 blue blister packs will be dispensed, both containing 30 tablets of the active experimental drug or placebo and 2 white blister packs both containing 30 tablets of the active comparator drug or

placebo. Dosage: administer one (1) tablet of the experimental placebo drug (blue blister pack) and then one (1) tablet of the active comparator drug (white blister pack), orally, once a day, in the morning. If the research participant forgets to take a dose of the medication, it should be administered as soon as he/she remembers and then the next dose should be taken at the usual time. However, if it is close to the time for the next dose, the participant should not take the missed dose; the minimum interval of 6 hours between doses must be respected. The participant should not take a double dose to make up for a missed dose.

SUPPLY OF STUDY MEDICATIONS

The study medications will be provided by Libbs Pharmaceuticals, in sufficient quantity to meet the treatment of research participants during the study in appropriate packaging and duly identified in accordance with the Manual for submitting quality data regarding investigational products used in clinical studies – synthetic and semi-synthetic medications (ANVISA, 2019).

PACKAGING AND LABELING

The investigational products will be supplied in packaging that will have space for the identification of the research participant, the site code, the corresponding visit and the return visit. They will also have recorded storage and conservation precautions, instructions for use, content, expiration date and random number.

The investigational products will be dispensed in blister packs. Participants randomized to the experimental drug will receive at V0, V1 and V2 two blue blister packs (figure 8) which will both contain 2 blisters with 15 tablets of the experimental drug (totaling 30 active tablets in each) and two white cartons (figure 7) which will contain 3 blisters with 10 placebo tablets of the comparator drug (totaling 30 placebo tablets in each). Participants randomized to the comparator drug will receive two blue blister packs (figure 8) containing 2 blisters with 15 placebo tablets of the experimental drug (totaling 30 placebo tablets in each) and two white blister packs (figure 7) containing 3 blisters with 10 active tablets of the comparator drug (totaling 30 active tablets in each). Pay attention to the correct identification of the information on the cartridge, as the identification of the returned study medications is necessary for their accounting and evaluation of the research participant's adherence to treatment.

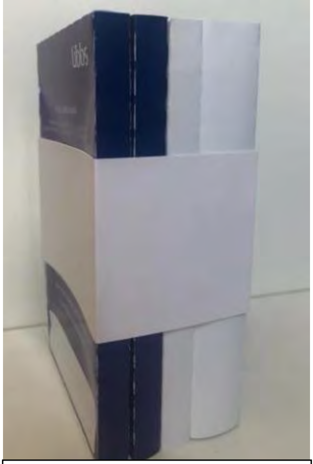

Figure 7: illustrative figure of the Kit, contains 1 box of experimental drug and 1 box of comparator drug.

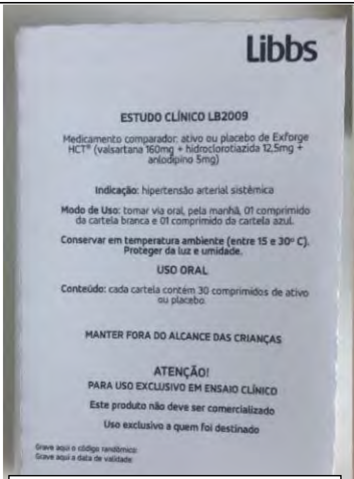

Figure 8: illustrative figure of the comparator drug.

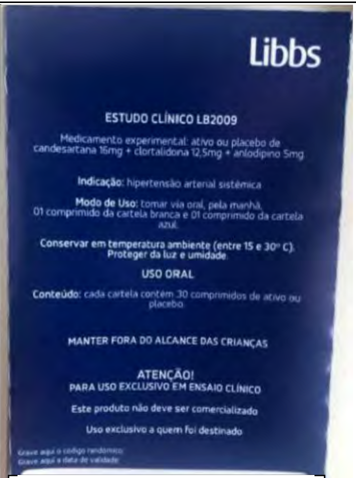

Figure 9: illustrative figure of the experimental drug.

STORAGE

Study medications must be stored in a restricted access location, in locked cabinets. Only authorized persons may have access to these products and access control to the storage location must be available for verification during Monitoring or Auditing. Study medications should be stored at room temperature (between 15°C and 30°C), protected from light and moisture.

Temperature deviation of medication stored at the research site

Temperature deviation or change occurs when, for any period of time, the investigational products are exposed to temperatures outside acceptable storage conditions (less than 15ºC or greater than 30ºC). If a temperature deviation occurs at the research site, the following procedure must be conducted: Quarantine the affected IP in; Inform the monitor responsible for the site by phone or email about the deviation and send a document via email with a record of the deviated temperatures, as well as the list of affected medications. Under no circumstances study medication impacted by a temperature deviation be dispensed/administered to the research participant before it has been evaluated by Libbs Pharmaceuticals. The site must separate the medication from the rest of the stock, always maintaining storage conditions and temperature control, in addition to preserving the integrity of the packaging. No direct identification should be inserted into the medication cartridges until it has been evaluated by Libbs Pharmaceuticals. For any temperature deviation reported, an instruction/notification will be sent by Libbs Pharmaceuticals or ARO as quickly as possible, defining the procedure that the site must conduct regarding quarantined medications. This instruction/notification should be filed together with the other study documents. If all available medication is quarantined, the site will need to replenish supplies immediately.

Temperature deviation of study medications dispensed to the participant

Whenever the site is contacted by the participant, notifying the occurrence of a temperature deviation of the medication dispensed (during transportation or storage at the participant's home), the following procedure must be performed: Inform the monitor responsible for the site by phone or email about the deviation and send a document via email with the report of the deviation that occurred and wait for a response regarding authorization to use the study medication or not; Inform the participant of the conduct defined by Libbs Pharmaceuticals:
Medication authorized for use: participant may use the medication as prescribed; Medication not approved for use: participant must return to the research site to return medication and receive a new one. All actions must be documented in a source document. Under no circumstances may study medications impacted by a temperature deviation be used by a study participant without prior authorization from Libbs Pharmaceuticals and/or ARO.

RECEIPT, DISPENSING AND ACCOUNTING OF STUDY MEDICATIONS

Study medications will be transported to the research site accompanied by an invoice and shipping form describing the product in question. If the quantity, validity or identification of the medication sent is discrepant in relation to the quantity described in its invoice or shipping form, or if the product is damaged in any way, Libbs Pharmaceuticals must be immediately contacted and the products segregated until the sponsor's evaluation.

The investigator, or a professional delegated by him/her, will be responsible for delivering the study medications to the research participant, for their collection, storage and accounting by completing the IP control forms. In cases of discrepancies, these must be documented with the appropriate reason. The dispensing will be conducted respecting the randomization result, when the research participant is included in the study.

During monitoring visits, study medication checks will be performed. All used or unused study medications must be returned to Libbs Pharmaceuticals or the authorized partner, together with the packaging, by the end of the study and will be destroyed in accordance with current legislation.

STUDY BLINDING

Due to the fact that the experimental/comparator drug has different organoleptic characteristics, to allow blinding of the study, the *Double-Dummy* method will be used, where both groups will administer two forms of the medications (active and placebo), thus preventing any violation of blinding. Thus, a placebo of the investigational product (combination candesartan cilexetil 16mg + chlorthalidone 12.5mg + amlodipine 5mg) and a placebo of the comparator product Exforge HCT® (valsartan 160mg + hydrochlorothiazide 12.5mg + amlodipine 5mg) were developed, ensuring the same organoleptic characteristics of the active ingredients, respectively, as shown in the table below.

Table 3. Organoleptic characteristics and description of the comparator drug and placebo of the comparator drug:

	**Specification**

	Comparator drug	Placebo of the comparator drug
Description	Appearance: White, biconvex, oblong coated tablet, engraved with “NVR” on one side and “VCL” on the other.	Appearance: White, biconvex, oblong coated tablet, engraved with “NVR” on one side and “VCL” on the other.
Primary packaging material:	Aluminum blister – aluminum	Aluminum blister – aluminum
Image:	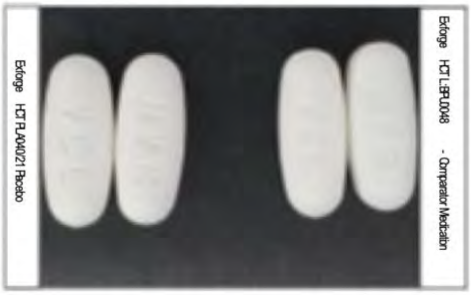	

Table 4. Organoleptic characteristics and description of the experimental drug and placebo of the experimental drug:

	**Specification**	
	**Experimental drug**	**Placebo of the experimental drug**
Description	Appearance of double-layer tablet: circular, biconvex tablet, salmon-colored in one layer and white in the other.	Appearance of the double-layer placebo tablet: circular, biconvex tablet, salmon-colored in one layer and white in the other.
Primary packaging material:	Aluminum blister – aluminum	Aluminum blister – aluminum
Image:	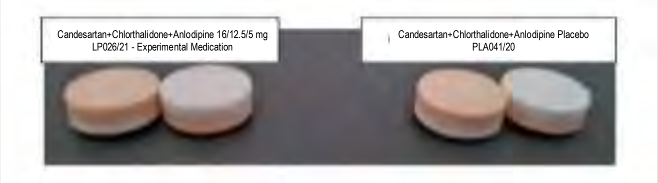	

Treatment of Group 1 (combination candesartan cilexetil 16mg + chlorthalidone 12.5mg + anlodipine 5mg): The research participant will administer, once a day, 01 **active** tablet of the experimental drug (combination candesartan cilexetil 16mg + chlorthalidone 12.5mg + amlodipine 5mg), plus 01 **placebo** tablet of the comparator Exforge HCT® (valsartan 160mg + hydrochlorothiazide 12.5mg + amlodipine 5mg), both orally.

Treatment Group 2 Exforge HCT® (valsartan 160mg + hydrochlorothiazide 12.5mg + anlodipine 5mg): The research participant will administer 01 tablet of the **active** comparator Exforge HCT® (valsartan 160mg + hydrochlorothiazide 12.5mg + amlodipine 5mg) plus 01 tablet of the **placebo** of the experimental drug (combination candesartan cilexetil 16mg + chlorthalidone 12.5mg + amlodipine 5mg), both orally.

To facilitate the understanding of the research participants regarding the dosage guidelines, a blue blister pack was created to be used for the experimental drug and its placebo, and a white blister pack was created to be used for the comparator drug and its placebo. In this case, the research participant should be instructed to simultaneously administer 1 tablet from the blue blister pack and 1 tablet from the white blister pack.

To enable blinding, the comparator drug Exforge HCT® will undergo a repackaging process, so that the information present on the blister of the original packaging of the comparator drug does not allow the identification of the treatment by the research participant and to enable a similar form between the comparator and its placebo, ensuring the blinding of the same. The material originally used for the packaging of the comparator drug is aluminum/aluminum blister and the proposal is to maintain the same material as the primary packaging, adapting the quantity of tablets per blister.

The repackaging process consists of removing the tablets from the original aluminum/aluminum blister (with engraving), to an aluminum/aluminum blister without engraving, in a certified area, complying with current good manufacturing practice standards. The packaging material used will be of the same composition (polyamide/PVC/aluminum) as the comparator drug, maintaining protection and guaranteeing the quality of the product during the expiration date informed by the manufacturer on the product packaging. Finally, the comparator drug will undergo the secondary packaging process to ensure the blinding of the study.
The transport of medications to the research site will be done by a company specialized in the transport of clinical research products. The medications will be stored in the research centers according to the sponsor's guidelines, described in the protocol. As described above, regarding the comparator drug, it will not undergo any change/modification in the integrity of the tablet. The modification only refers to the removal of the tablets from the original blister to a new blister without engraving, maintaining the same composition of aluminum/aluminum packaging material. Therefore, the

company ensures that there will be no pharmacokinetic changes to the comparator drug.

To ensure that this change occur safely, a risk analysis of the entire process was conducted and described in detail in document 08 “Description of placebo and comparator”, incorporated into the DCDD, thus ensuring that there was no impact on the quality of the product. According to the Ishikawa diagram below, the risks related to methodology, materials, operation, machines, environment and measurement were identified and mapped and for each of these risks a discussion was held in the document and actions to mitigate them were presented.

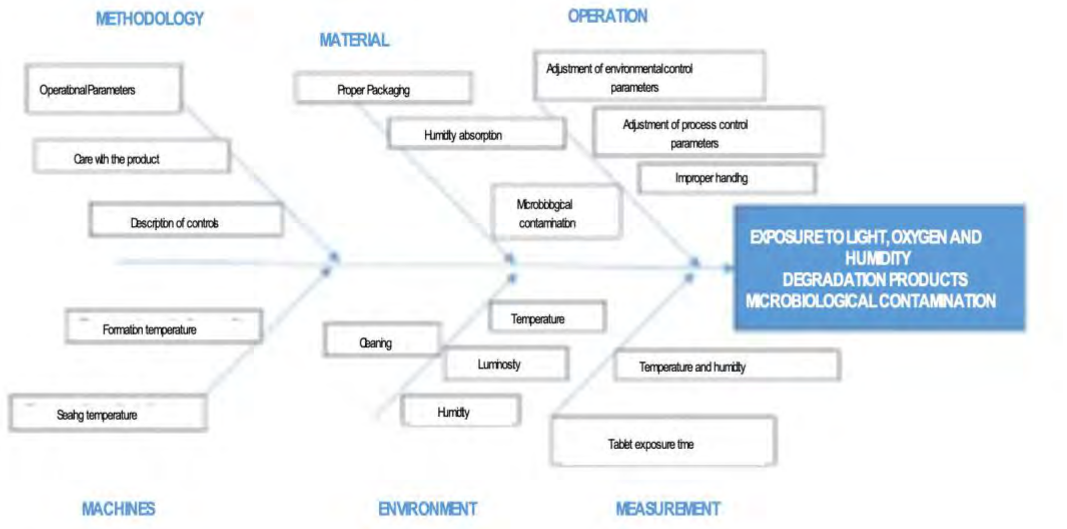

Figure 10: Ishikawa diagram based on multidisciplinary risk analysis.

As previously reported, the blisters of the experimental/comparator drug and their respective placebos were placed in different blister packs, which prevents the viewing of the batch numbers and the actual expiration date engraved on the blister. Therefore, each blister pack will have a unique random number, which will allow tracking of the batch and treatment group in case it is necessary to break the study's blinding.

This procedure ensures that during the study, if there is a need to unblind the code for a participant for safety reasons, the corresponding random number will allow the treatment to be revealed while maintaining the omission of the batch number, and consequently preserving the blinding of the other participants who received medication from the same batch.

Each participant will receive from the professional, delegated by the Principal Investigator, the tablets corresponding to the treatment, according to the allocation generated by the randomization list. They will be dispensed in blister packs containing the same information for all treatment groups. At the research site, there must be a double check of the random code engraved on the blister packs before dispensing the IP to the research participant. The blister packs that each participant will receive will be non-transferable and confidential to the other research participants, therefore the research site must guide this conduct at the time of dispensing. To accounting and verify treatment adherence , the professional delegated by the investigator must collect the blister packs and count the returned tablets.

UNBLINDING STUDY CODE

The treatment given to a given participant may be revealed during the study when knowledge of this is essential to ensure the participant's safety. Unblinding the nature of the study for other reasons will be considered a protocol violation. The principal investigator should, if possible, contact the study sponsor before unblinding randomization code. After the database is locked, the randomization codes will be unblided so that statistical analysis can be performed.

MEDICATIONS / TREATMENTS

All medications, procedures and treatments performed within the period of thirty (30) days prior to the start of the clinical study (previous) and during the progress of the clinical study (concomitant) must be documented in both medical record and CRF.

PROHIBITED MEDICATIONS/TREATMENTS

The research participant may not take part in the study if he/she is using the medications described in Table 5 for the periods prior to the randomization visit [V0], or if he/she is using any medication or treatment that the investigator considers to interfere with the treatment, in the evaluation of the research results, or that pose a risk to the participant. If the use was made before V0 and the *washout* period established for each medication was not respected, the participant cannot be randomized; if the use occurred after randomization, the participant must be discontinued from treatment, as mentioned below. According to the eligibility criteria, study participants will be undergoing prior treatment with dual antihypertensive therapy, from different therapeutic classes, prior to randomization. Therefore, they should be instructed in V-1 not to use the medication on the date of the randomization visit. However, if the participant has used the usual antihypertensive medication on the same date as the visit, but before randomization, he/she should be instructed to start using the IP the following day, in the morning.

The use of antihypertensives of any class (except IP) is prohibited from V0 onwards. However, participants with a hypertensive pseudocrisis and who have used ONE dose of antihypertensive medication not provided for in the protocol in the interval between V0 and V2 will not be discontinued from treatment, if this medication has not been used for a period of less than or equal to 24 hours before the BP measurement during the visit. The use of any other antihypertensive medication is not permitted in the period between V2 and FV. Therefore, if the research participant uses any antihypertensive medication after V2, the treatment must be discontinued.

Table 5: Prohibited medications

Drug/Therapeutic class	Expiration Date (before V0)
Anorectics	4 days
Antineoplastic agents that inhibit VEGF (vascular endothelial growth factor)	500 days
Intravenous human recombinant erythropoietin	6 days
Subcutaneous human recombinant erythropoietin	12 days
Levometadil	25 days
Atazanavir	8 days
Domperidone	4 days
Droperidol	20 hours
Lacosamide	6 days
Lithium	15 days
Antihypertensives of any class including propranolol, (except only IP)	from V0

9. MEDICATIONS ALLOWED WITH RESTRICTION

The medications reported in items 1.2.1.2.4, 1.2.2.2.4 and 1.2.3.2.4 of this protocol may be permitted, but with restrictions, according to the investigator's analysis. Due to the possibility of drug interactions, the principal investigator must evaluate the need to use these medications and whether the benefits outweigh the risks involved, ensuring the safety of the research participant.

ADVERSE EVENTS

The occurrence of adverse events must be documented by the investigator in the medical record (source document) and in the clinical record (e-CRF) of the research participant, at any time during the study.

IDEFINITION AND CLASSIFICATION

An adverse event (AE) is defined as any adverse medical occurrence in a patient or clinical trial participant to whom a

pharmaceutical product has been administered, and which does not necessarily have a causal relationship to the treatment. As a result, an AE can be any temporarily unfavorable and unintended sign, symptom, or illness (including laboratory test results outside the reference range) associated with the use of an investigational product, whether related to it or not. All adverse events (Serious and Non-Serious) observed or reported after consent has been obtained through the ICF, regardless of the possible causal relationship with the study medication, must be reported on the Adverse Events page(s) of the Clinical Record (e-CRF), as well as in the medical record (source document) of the research participant. Events involving adverse medication reactions, diseases manifested during the study, or exacerbations of preexisting diseases must be reported. In the case of research participants with a relevant prior clinical history, the participant's report must be considered and this condition must be reported in the medical record, in the clinical record of the initial visit specifying the date of diagnosis, follow-up history (frequency, intensity of crises) and the treatment followed. Previous conditions will not be considered adverse events unless there is an exacerbation or worsening of the condition. Elective procedures planned before the start of the study will not be considered adverse events, even when performed during the study period, excep forcomplications occurring during the procedure.

CLASSIFICATIONS AND EVALUATIONS MADE BY THE INVESTIGATOR REGARDING THE ADVERSE EVENT

The investigator must evaluate each adverse event according to the following parameters described below and according to the following definitions: Classification of severity of adverse events: A Serious Adverse Event is one that results in any of the following outcomes: death; life threatening; persistent or significant disability/incapacity; requires hospital stay or prolongs hospitalization; congenital anomaly or birth defect; any suspicion of transmission of an infectious agent through a medication or; clinically significant event. A clinically significant event is considered any occurrence that, based on appropriate medical evaluation, may harm the participant and/or require medical or surgical intervention to prevent any of the other occurrences mentioned in items a – f mentioned above.

Note: hospitalization is medical care that requires hospitalization. The participant's stay for a period of 24 hours or more in a hospital unit is also considered hospitalization. To define clinically significant events, the investigator must use medical evaluation, considering the clinical relevance of the adverse event to the participant's health. The *European Medicines Agency* (EMA) Important Medical Events List can be consulted to support the evaluation of the clinical relevance of the adverse event. All adverse events that do not fall within the situations described in items a – g above are considered non-serious.

Upon receiving the report, the sponsor must also evaluate the severity of the adverse event, following the same criteria indicated above. In the event of discrepancies in the evaluations of the investigator and the sponsor regarding severity, the situation should be discussed so that the severity to be considered for the adverse event in question can be defined. If the divergence is maintained, the report should be considered serious, i.e., a more conservative scenario.

Intensity Classification of Adverse Events
Mild: asymptomatic adverse event or with mild symptoms; only clinical or diagnostic observations; intervention not required.
Moderate: adverse event with indication for minimal, local or non-invasive intervention; limitation in instrumental activities of daily living (*Activities of Daily Living* (ADL))*.
Severe: clinically significant but not immediately fatal adverse event; indication for hospitalization or prolonged hospitalization; participant disability; limitation in self-care activities of daily living (ADL)**.

Activities of Daily Living (ADL)
Instrumental ADL: refers to preparing meals, shopping for groceries or clothes, using the phone, managing money, etc.
** Self-care ADLs refer to bathing, dressing and undressing, eating, using the toilet, taking medications, and staying bedridden.

Causality Classification of Adverse Events: To evaluate causality, the causality evaluation method of the World Health Organization (WHO) or *WHO-UMC (World Health Organization - Upssala Monitoring Centre)* must be used. The WHO classification categories are:

Table 6: WHO classification categories

Classification	Description	Presence of other causes
Definite	Clinical event or laboratory test abnormality that occurs within a plausible time frame in relation	No

	to product administration;<br>It cannot be explained by an underlying disease or by other products or even chemicals;<br>The response to product withdrawal must be plausible (pharmacologically and pathologically);<br>The event is pharmacologically or phenomenologically defined (e.g., a medical disorder or pharmacologically recognized diagnosis);<br>Confirmed using a satisfactory rechallenge procedure, if necessary.	
Probable	Clinical event or abnormality in laboratory test, which occurs within a reasonable period of time after administration of the product;<br>Unlikely to be attributed to a concomitant disease or other products or chemicals;<br>Presents a clinically reasonable response to product withdrawal (dechallenge);<br>Rechallenge information is not required to complete these criteria.	Yes
Possible	Clinical event or abnormality in laboratory test, which occurs within a reasonable period of time after administration of the product;<br>It can also be explained by concomitant illness or other products or chemicals;<br>Information about discontinuing use of the product (dechallenge) may be missing or unclear.	Yes
Unlikely	Clinical event or abnormality in a laboratory test, which, in relation to the time of administration of the product, makes a time relationship unlikely (but not impossible);<br>Other products, chemicals and/or underlying diseases provide plausible explanations.	Yes
Conditional	Clinical event or laboratory test abnormality;<br>More data are needed for a proper evaluation;<br>Additional data is under evaluation.	Not applicable.<br>Insufficient data
Not assessable*	Report suggests an adverse reaction<br>Cannot be evaluated because the information is insufficient or	Not applicable.<br>Insufficient or contradictory data

	contradictory<br>Data cannot be supplemented or verified	

In the event of discrepancies in the investigator's and sponsor's evaluations regarding causality, the situation must be discussed so that the causality to be considered for the adverse event in question can be defined. If the divergence is maintained, the more conservative causality should be considered. Predictability Classification of adverse events The sponsor's Pharmacovigilance is responsible for evaluating the predictability of adverse events based on the investigator's brochure.

Information on the need for treatment of the adverse event

The investigator must report whether there was a need for clinical, pharmacological or surgical treatment, as well as any other medical conduct, to resolve the adverse events presented, as follows: No action; Medication administered; Non-drug therapy administered; Surgery performed; Hospitalization; Others such as observation without any other intervention, clinical follow-up, repetition of tests, referral to specialized medical service, etc.

Information on action taken regarding the investigational product because of the adverse event

The investigator must record the action taken regarding the Investigational Product following the classification below: Unmodified dose; Medication interrupted (when there is a temporary interruption of the medication); Discontinued medication (when the medication is permanently discontinued); Dose increase; Dose reduction; Not applicable (when the adverse event occurs before the first administration of the investigational product).

Information on the progression of the adverse event presented.

The investigator must report the evolution of the adverse event (clinical outcome), considering the classification: Recovered; Not Recovered; In recovery; Recovered with sequelae; Got worse; Resulted in death. In cases of death, it is essential to record the *causa mortis* data.

REPORTING OF SERIOUS ADVERSE EVENTS (SAEs)

The investigator must forward by email to farmacovigilancia@libbs.com.br and drugsafety.aro@einstein.br, and/or via e-CRF, within a maximum period of 24 hours of awarereness of the event, the SERIOUS ADVERSE EVENT REPORTING FORM, completed and signed, with a copy to the ARO Clinical Research (monitor responsible for the site). Additionally, the e-CRF must be completed in the adverse event tab, preferably within 24 hours, with a maximum period of 5 working days, from the date the event was known.
Note: Additionally, the Research Site is responsible for notifying any SAE to the EC/CONEP system, in accordance with current regulations.

The sponsor must notify Anvisa of unexpected serious adverse events occurring in Brazil, the causality of which is possible, probable or defined in relation to the investigational product. Adverse events that are fatal or that pose a threat to life must be reported to Anvisa within a maximum of seven (7) calendar days from the date the sponsor becomes aware of the case. Additional information on following-up of the previously mentioned adverse events must be shared with Anvisa within eight (8) calendar days from the date of notification to the sponsor.

All other adverse events that are serious, unexpected and with possible, probable or defined causality in relation to the investigational product (except death and threat to life) must be reported to Anvisa within fifteen (15) calendar days from the sponsor becoming aware of the case. The sponsor must inform investigators involved in the clinical trial about the occurrence of serious and unexpected adverse events (whose causality is possible, probable or defined) and adopt procedures for updating the Investigator's Brochure, in addition to risks and benefits assessment for study participants. If necessary, in emergency cases during an SAE, the randomization code may be broken without authorization from the sponsor. In these cases, the responsibility unblinding lies exclusively with the investigator, without any involvement of the sponsor in the decision. Instructions for unblinding should be clearly described in the study protocol and the sponsor should be notified as soon as possible about the unblinding.

FOLLOWING-UP ADVERSE EVENT REPORTS

The investigator must follow up on all adverse events until there is an outcome: full recovery, clinically acceptable

stability or documented loss to follow-up (minimum of three attempts). At the time of database locking, all information must be up to date.
After locking the database, following-up of open adverse events should be conducted by the investigator in accordance with clinical practice. All adverse events must be managed and affected participants followed by the principal investigator and his/her team until resolution or stabilization.

After the completition of the study, the investigator must inform Libbs Pharmacovigilance within 24 hours of becoming aware, using the SERIOUS ADVERSE EVENT REPORTING FORM, of the SAE reported to him spontaneously, which occurred within 30 days after the participant completed or discontinued the study, if there is a possibility of a causal relationship between the adverse event and the investigational product.

REPORTS OF DIRECT AND INDIRECT PREGNANCY

The investigator must send by email to farmacovigilancia@libbs.com.br and drugsafety.aro@einstein.br within a maximum period of 24 hours from awareness of the event, the PREGNANCY NOTIFICATION FORM completed and signed with a copy to the ARO Clinical Research (monitor responsible for the center). Additionally, the e-CRF must be completed in the adverse event tab, preferably within 24 hours, with a maximum period of 5 working days, from the date the awareness.

Pregnancy follow-up

The investigator must follow-up the progress of the pregnancy, postpartum period and psychomotor development of the child, even after the interruption of the treatment of the research participant in the study, considering that such information is important for evaluating the safety profile.
For research participants and partners of participants who eventually become pregnant after consent has been obtained through the informed consent form, quarterly pregnancy follow-up will be required, as described: End of the 1st quarter: preferably between the 12th and 16th weeks; End of 2nd quarter: preferably between 24th and 28th weeks; End of 3rd quarter: preferably between 36th and 40th weeks; Postpartum: preferably 15 to 20 days after birth; In the 6th month of the child's life; In the child's first year of life.

The investigator must send the information by email to Pharmacovigilance, with a copy to ARO Clinical Research (monitor responsible for the center), by completing the PREGNANCY AND POST-PARTUM FOLLOW-UP FORM. If the investigator identifies complications during pregnancy such as spontaneous abortion, elective interruption, abnormal birth or congenital anomaly, he/she must immediately report to the Libbs Pharmacovigilance department with a copy of the clinical research within 24 hours of awareness, by filling out the PREGNANCY AND POST-PARTUM FOLLOW-UP FORM.

Note: Complications during pregnancy such as spontaneous abortion, elective interruption, abnormal birth or congenital anomaly should be classified as a serious adverse event and evaluated as such.

If a research participant eventually becomes pregnant after consent has been obtained through the informed consent form, but before the administration of the investigational product, the investigator must forward only the initial report of the occurrence of the pregnancy, without the need to conduct pregnancy follow-up described above. In this case, the participant must discontinue treatment, i.e., not start using the IP.

Notification to the EC/CONEP system

The notifying investigator will receive from the ARO the NI (Notification to Investigators) in CIOMS format, of the SAE evaluation.All participating investigators should be notified of unexpected serious adverse events related.
After receiving the CIOMS form, the investigator must notify the EC/CONEP system in accordance with circular letter 13/2020.

Note: Libbs Pharmacovigilance will forward the NI in CIOMS format to the ARO with a copy to Libbs Clinical Research.

STATISTICAL ANALYSIS PLAN

The sample size is calculated based on the primary outcome of the study: Mean change in systolic blood pressure (SBP) 12 weeks after starting treatment compared to baseline (FV – V0).

Hypotheses:
H0: Difference between treatment effects is greater than 3 (H0: ΔT–ΔC > 3 mmHg)
H1: Difference between treatment effects is not greater than 3 (H1: ΔT–ΔC ≤ 3 mmHg), where:
Δ T = Mean change in SBP in the Treatment group;
Δ C = Mean change in SBP in the Control group;
Margin of non-inferiority between changes: Δ = 3 mmHg.
Based on data obtained from literature considered relevant by physicians (references below), the following assumptions are made for the calculations: Margin of non-inferiority: Δ = 3 mmHg; Standard deviation of reduction: SD = 14 mmHg; Unilateral alpha = 0.05; Power of the test = 85%.

The power of the test was set slightly above the generally used value of 80%, with the intention of ensuring the lowest possible type II error. The non-inferiority margin of 3 mmHg was defined based on a non-clinically relevant difference in the evaluation of systolic blood pressure. It is also noteworthy that the margin of 3 mmg Hg in systolic blood pressure preserves at least 50% of the magnitude of the effect observed in randomized clinical trials comparing combined triple therapy in a single tablet versus usual treatment or combined double therapy (Table 7)

Table 7. Descriptive analysis of randomized studies including triple therapy in a single tablet.

Study	Year	N	Medication	Comparator	Titration	Δ SBP	Δ DBP	Highest SD SBP	Length
Calhoun et al.	2009	2271	Anl/Val/HCTZ (10/320/ 25 mg)	Anl/Val (10/320 mg) or Val/HCTZ (320/25 mg) or Anl/HCTZ (10/25 mg)	Yes (forced up-titration)	8.2-6.2 (LSM)	5.3-3.3 (LSM)	14.5	9 weeks
Chrysant et al.	2010	2492	Anl/Olm/ HCTZ (10/40/ 25 mg)	Olm/Anl (40/10 mg) or Olm/HCTZ (40/25 mg) or Anl/HCTZ (10/25 mg)	No	9.6-7.1 (LSM)	6.7-3.8 (LSM)	15.1	12 weeks
Ferdinand et al.	2011	412	Anl/Ali/HCTZ (5/150/ 12.5 mg-10/300/25m g)	Anl/Ali (5/150 mg-10/300mg)	Yes (forced up-titration	7.0 (LSM)	3.1 (LSM)	9.0	8 weeks
Maladkar et al.	2012	220	Anl/Tel/HCTZ (5/40/12,5 mg)	Tel/HCTZ (40/12,5 mg)	No	7.88	3.07	16.0	12 weeks
Webster et al.	2018	700	Tel/Anl/CLOR (20-40/2.5-5.0/12.5-25)	At the discretion of the investigator/recommendation of SAH guidelines	Yes	8.8	4.6	11.6	6 months

Keys: Anl, anlodipine; Ali, aliskiren; CLOR, chlorthalidone; SD, standard deviation; SAH, systemic arterial hypertension; HCTZ, hydrochlorothiazide; LSM, *least square means*; Olm, olmesartan; Tel, telmisartan; Val, valsartan. The assumed standard deviation was found in the literature, particularly in Smith, T. R, 2010 (*Table 2. Mean changes from core baseline in MSSBP*) and in Calhoun, D.A, 2009, who uses this standard deviation value in calculating the sample size. Estimation of the standard deviation is planned in an interim analysis, with the objective of confirming the value estimated for the calculation.

Conclusion:
Using a Student's t-test, assuming the above premises, the non-inferiority of the Treatment in relation to the Control can be demonstrated with the evaluation of 314 participants in each group, with a power of 85% (total = 628 evaluable participants). Considering approximately 10% of losses/violations, 698 (349 per group) should be randomized. In order to ensure that the number of evaluable participants is achieved, if the *dropout* rate or selection failure is higher than expected, participants may be replaced.

Formula used:
Sample size was calculated using the PASS 2021 software, version 21.0.2, considering the test of means for two independent groups and balanced samples. The sample size can be obtained by the following expression (Chow, (2003),

$$n = \frac{(z_\alpha + z_\beta)^2 * \sigma^2}{(\varepsilon - \delta)^2}$$

where, α is the one-tailed significance level; 1-β is the power of the test; Z_k is the (1- k)-th percentile of the standard Normal distribution; is the standard deviation of the SBP variable, assumed equal for both groups; $\varepsilon=\mu 2 - \mu 1$ is assumed equal to 0, and $\Delta > 0$ is the non inferiority margin.

ANALYSIS POPULATIONS

The following populations are defined for the analyses:

Safety Analysis Population: population in which all randomized participants who receive at least one dose of the investigational product will be included. Research participants will be classified in the safety analyses according to the medication they received during the study.

Intention-to-Treat (ITT) Analysis Population: All randomized participants will be included in the ITT Analysis Population and will be analyzed according to the treatment group to which they were randomized, regardless of the treatment received.

Per-Protocol Analysis Population (PP): all research participants from the ITT population will be included in the Per-Protocol Analysis Population (PP), except participants with: Major protocol violations that interfere with efficacy of treatments to be defined prior to locking the database; Lack of treatment adherence (adherence less than 80% or greater than 120%) between V0 – V1, V1-V2 and/or V2–;
Use of other antihypertensives after randomization; Other protocol violations that may affect efficacy evaluation, to be described and duly justified in the final/statistical report. All protocol violations will be documented during the study.

The primary efficacy analysis will be performed considering primarily the ITT Population and additionally the PP Population. Secondary efficacy analyses will be performed considering the ITT Population, and safety analyses will be performed considering the Safety Population. Although per-protocol analysis was previously used in non-inferiority studies because it is more conservative (in this specific non-inferiority situation), its quality has been greatly questioned by more recent analyses (Hernán, Miguel, 2017; Mo, Yin, et al, 2020). It is currently not considered a truly randomized analysis, since the groups are not defined by a random method, but rather by characteristics defined after randomization such as adherence to both protocol and treatment. More recently, some journals such as the New England Journal of Medicine have avoided this type of (per protocol) analysis. The ITT analysis maintains the benefit of randomization by preventing selection bias, in particular confusion, maintaining true comparability between the study groups.

STATISTICAL METHODS

Participant disposition and sample characterization

Statistical analyses will be performed after the database lock and clousure of the data collection system (clinical record), using SAS® software (version 9.4 or later). The disposition of participants who were screened, randomized, received medication, discontinued prematurely or completed the study, and the reasons for discontinuation will be summarized by treatment group. Number (and percentage) of participants with protocol deviations will be summarized by treatment group with details of the type of deviation. At least the following protocol deviations below will be described:
Participants randomized who do not meet any inclusion or exclusion criteria;
Participants who received the incorrect treatment at any point during the study; Participants who received prohibited concomitant medication.

Demographic characteristics, pre-existing clinical conditions, medical history, previous treatments reported at baseline will be summarized by treatment group, considering the ITT Population. Descriptive statistics will be calculated to summarize the data according to the type of each variable: absolute and relative frequency, and 95% CI, when appropriate, will be used for categorical and ordinal numerical variables, and number of valid observations, mean, standard deviation (SD), median, minimum and maximum values will be calculated for continuous numerical variables.

Safety Analyses

The following safety outcomes will be considered: Incidence of adverse events (AE) documented from the first dose of PSI up to 30 days after the conclusion of the treatment provided for in the protocol; Incidence of any AE occurring after obtaining consent through the ICF up to 30 days after the conclusion of the treatment provided for in the protocol;

Proportion of participants with changes in laboratory tests considered clinically relevant according to the investigator's criteria, at the baseline visit (V0) and at the final visit (FV) of the study. Proportion of participants with clinical or physical alterations, considered clinically relevant according to the investigator's criteria, at visits V1, V2 and FV compared to baseline (V0); Absolute change in vital signs measured at visits V1, V2 and FV compared to the baseline visit (V0).

Number and percentage of participants with adverse events will be presented along with the respective 95% CIs, by treatment group, and according to severity, intensity, causality with study medications, action taken and outcome. Adverse events reported during the treatment period will be coded using the MedDRA (Medical Dictionary for Regulatory Activities) dictionary. Serious adverse events or those that led to permanent discontinuation of treatment will be listed. All adverse events recorded in the study will be organized in a list, using the MedDRA (PT and SOC terms), containing information on the date of onset, severity, intensity, causality, action taken, outcome, with identification of the participant and treatment group. Concomitant medications will be coded using the ATC (Anatomical Therapeutic Chemical) classification system and will be summarized descriptively by treatment group.

Vital signs, physical examinations results, and laboratory tests will be summarized using descriptive statistics for each treatment group at each evaluation time. The absolute change in vital signs from visit 1, visit 2, and final visit to visit 0 will be calculated and displayed by treatment group. The percentages of participants with clinically relevant changes will be summarized by treatment group: in laboratory tests from the final visit to visit -1; in laboratory tests from visits 1 and 2; in clinical and physical examination from visits 1, 2, and final visit to visit 0;

Effectiveness Analyses

SBP and DBP measurements will be summarized using appropriate descriptive statistics, as outlined in Section 11.2.1, in tables and graphs, by time point and by treatment group. The number of participants achieving target pressures and respective proportions will be described and displayed in tables and graphs, at each time point and for each treatment.

Primary Efficacy Analysis

The primary hypothesis of efficacy is the non-inferiority of Group 1 compared to Group 2 (control), in the mean change of systolic blood pressure (SBP), 12 weeks after the start of treatment (FV) compared to the baseline (V0). With ΔT and ΔC being the change obtained in SBP (FV – V0) of Groups 1 and 2, respectively, the conclusion of non-inferiority will be given if the upper limit of the CI (90%) of the difference between the groups does not exceed 3 mmHg: $\Delta T - \Delta C \leq 3$.

The data will be analyzed and the confidence interval constructed using a mixed model for repeated measures (MMRM), with a random effect of site, fixed effect of treatment, time and treatment by time interaction, evaluation times as repeated measures and SBP valuesat V0 as a covariate. This analysis will be performed using the ITT Population. As a sensitivity analysis, the 95% confidence intervals for this difference between the groups will be presented in the ITT and PP Populations. Furthermore, participants without post-baseline data will not contribute to analyses performed with the MMMR approach, but will contribute to sensitivity analysis using data imputation. Because of this, no additional population, such as a modified ITT Population including only participants with post-baseline data, will be defined.

Secondary Efficacy Analyses

Analyses of SBP and DBP change will be performed using the same MMRM model described for the primary efficacy analysis. The results of the model for the effects of treatment group, time and interaction will be presented, as well as the CI (95%) of the differences between the groups at weeks 4, 8 and 12 for DBP and at weeks 4 and 8 for SBP. The following proportions of participants will be compared between treatment groups at Weeks 4, 8 and 12, using the Chi-Square test: Participants achieving target blood pressure SBP <140 and DBP <90 mmHg; Participants achieving SBP <120 mmHg; Participants achieving SBP <140 mmHg; Participants achieving DBP <90 mmHg; Participants with a reduction greater than or equal to 20 mmHg in SBP; Participants with a reduction greater than or equal to 10 mmHg in DBP.

The proportions will be calculated by the ratio between the number of participants reaching each outcome and the number of participants in the ITT population, at each time point. The MMRM model proposed for analyses of the primary objective and secondary outcomes related to numerical changes in SBP and DBP is used under the assumption of data normality. If normality is not confirmed, the generalized linear model proposed in Section 11.5 will be used as sensitivity analysis.

INTERIM ANALYSIS

When 50% of the study participants achieve the primary outcome of 12 weeks, the standard deviation (SD) value of the SBP changes (FV-V0) will be estimated, with the sole purpose of confirming the variability used in the sample size calculation. This estimate must occur in a blinded procedure (analysis of aggregated data). As comparative analyses will not be performed between the groups, there will be no correction of alpha and beta expenses. The study sample size may be recalculated using the estimated SD, if it is above the assumption applied for the sample size calculation. However, regardless of the new standard deviation found during the interim analysis conducted on the analyzed sample, the current sample size (698 randomized participants) will not be reduced, and may only be maintained or increased.

The SD will be estimated through a mixed model adjusted to the change in SBP, with baseline as covariate, time as repeated measures and site as random effect. The estimate of the standard error of the mean least squares resulting from the model in week 12 will be considered for the calculation of the S.D. If S.D. > 14, the sample size should be recalculated using the S.D. estimated by the model. Otherwise, it will be maintained. For this interim analysis there will be no independent data evaluation committee. The calculations will be performed by the study's blind statistics, who will have restricted access to the e-CRF, guaranteed by the CRO responsible for implementing the clinical record and managing the data.

RANDOMIZATION AND ALLOCATION CONCEALMENT

Participants eligible for the study will be allocated in a 1:1 ratio to one of two treatment groups (Fixed combination of candesartan cilexetil 16mg + chlorthalidone 12.5mg + amlodipine 5mg or Exforge HCT®) according to the randomization list generated by means of a computational algorithm, available in R or SAS, according to a completely randomized regiment. Participants will be allocated via the Internet, using a module dedicated to randomization, integrated into the eCRF system, with access controlled by username and password. After the participant has registered and the site has completed the eligibility criteria in the system, a randomization number and the treatment to be assigned to the research participant, respecting the order of the randomization list generated prior to the start of the study, will be informed by the system. A randomization receipt with participant information, randomization number, treatment to be administered, process control and traceability transformation, the smallest value among the changes, in module, is planned to be added to all data. The Gamma distribution will be assumed as an alternative assumption to the Normal distribution, and its adherence to data will be verified through the Univariate procedure of the SAS statistical software. The assumptions will be made regarding primary and secondary numerical variables involved in statistical models, specifically the changes of Systolic Blood Pressure (SBP) and Diastolic Blood Pressure (DBP). The other outcomes (proportions) do not require evaluation in relation to data distribution. Further details are described in the SAP.

TREATMENT OF MISSING OR INVALID DATA

Data imputation will be used when there is no post-baseline data in the primary and secondary efficacy analyses, in relation to SBP change, as a sensitivity analysis for the ITT population. For multiple data imputation, techniques based on Pattern Mixture Models (PMM) (Ratitch et al., 2011) will be applied, under the assumption of missing data at random (MAR). Missing data from participants who permanently discontinue IP due to the use of another antihypertensive medication will be imputed using the *last observation carried forward* (LOCF) method from week 8 onwards, as they cannot be classified as missing data at random (MNAR). There will be no data imputation for analyses of categorical variables. All details about the methodology used for data imputation are described in the SAP.
Following the guidelines of ICH-E9 (Statistical Principles for Clinical Trials), if any data is statistically and clinically identified as spurious, the analyses will be performed with and without this data. If a difference is found between the results of the two analyses, an evaluation of the interference of the spurious data at the end of the study will be presented in the final report.

DATA MANAGEMENT

A system for randomization, data capture and management (eCRF), in compliance with FDA Code of Federal Regulations (21 CFR part 11), will be provided to the sites by the sponsor. Participant data will be entered into the system by the investigator or the delegated person(s). The system will be provided with features that contribute to improving the quality and consistency of data, enabling the traceability of changes made and ensuring the safety and integrity of the information collected.
Prior to the start of the study, processes will be established to check for inconsistencies and data coding that will be followed throughout the data collection phase. After solving all inconsistencies, coding medical terms and medications using pre-established dictionaries, obtaining the investigator's consent and identifying the analysis populations, the

database will be locked for statistical analyses. Data management of clinical studies conducted by Libbs Pharmaceuticals is conducted in compliance with current regulations.

ETHICAL AND REGULATORY CONSIDERATIONS

ETHICAL-REGULATORY ASPECTS OF RESEARCH

This study will be conducted in accordance with the protocol and research presented, following the bioethical references of Resolution No. 466 of December 12, 2012 of the National Health Council (MS), Operational Standard 001/2013 of the National Health Council (MS), as well as all its complementary resolutions. The protocol and all its contents, which include objectives, design, methodology, statistical considerations and organization of the study, were prepared and must be conducted in accordance with the international guidelines of the *International Council for Harmonization of Technical Requirements for Pharmaceuticals for Human Use* (ICH), in particular, the *Guideline for Good Clinical Practice* (ICH E6(R2), 2016) and the Resolution of the Collegiate Board of ANVISA – RDC No. 09 of February 20, 2015 and its updates, which provides for the regulations for conducting clinical trials with medications in Brazil.

According to the Resolution of the Collegiate Board – ANVISA – RDC No. 38, of August 12, 2013, amended by RDC No. 311 of October 10, 2019, the free provision of medications after the conclusion of the clinical trial must be made available to research participants in accordance with the Resolutions of the National Health Council. Furthermore, CNS Resolution No. 466 of 2012, item III.3.d, states that research must “ensure that all participants at the end of the study, on the part of the sponsor, have free and indefinite access to the best prophylactic, diagnostic and therapeutic methods that have proven to be effective”. It also adds in subitem (d1) that “access will also be guaranteed in the interval between the conclusion of individual participation and the end of the study, and in this case, this guarantee may be given through an extension study, in accordance with a duly justified analysis by the participant's attending physician”. It is in the sponsor's interest that foreseeable harms are avoided and that the burden on vulnerable research participants is minimized.

Ethical principles

According to National Health Council Resolution 466/2012, Operational Standard 001/2013 and complementary standards, research participants will be guaranteed full freedom to refuse to participate or withdraw their consent, at any stage of the research, without any penalty, and the maintenance of confidentiality and privacy during all stages of the research. The project will be registered on the Plataforma Brasil and will only be started after approval by the EC/CONEP system. The data collected from participants will be kept confidential and only the lead researcher and his team will have access. Before the initiation of the study, the protocol, the Informed Consent Form (ICF) and other information will be submitted for approval by the Ethics Committee. Any changes or additions to the protocol must be notified via amendments and submitted for evaluation by the same EC.

Regulatory requirements

The study will have all its clinical development in Brazil for registration purposes and may only be initiated after submission and approval of the Clinical Drug Development Case (CDDC) and the Specific Clinical Trial Dossier (SCTD) by ANVISA. The authorizing nature of the study will be granted through the issuance of a Special Notice (SN) issued by ANVISA, after analysis and approval.

ETHICS COMMITTEE (EC)

The clinical study must be previously approved in writing, in accordance with applicable legislation, by a EC duly registered with the National Research Ethics Commission (CONEP) through the issuance of the Consolidated Opinion. Submission of the clinical study protocol to the EC is the responsibility of the Principal Investigator (lead researcher), as well as following-up the status of this evaluation. No activities involving research participants may be initiated before approval by the Ethics Committee. Any changes to the clinical protocol, the Informed Consent Form (ICF) or other written information provided to research participants must be approved by the EC and documentation of this new approval forwarded to Libbs Pharmaceuticals. Records of the review and approval of all study documents must be maintained on file by the investigator and may be subject to inspection during the study and after its completion. In the event of changes that are necessary due to safety, in situations where there may be imminent risks to participants, these may be made prior to approval by the EC.

PROTOCOL/AMENDMENTS

All amendments to a clinical trial protocol must be documented, identifying the part of the protocol to be modified and its justifications, dated, signed between the parties (sponsor and principal investigator) and will be forwarded for approval by the EC and ANVISA (substantial amendments). Amendments may only be implemented after receiving approvals from the EC and ANVISA (substantial amendments), except when it is necessary to eliminate immediate risks to the safety of clinical trial participants, which may be implemented and notified to the applicable ethical-regulatory authorities.

Note: Non-substantial amendments must be submitted to ANVISA as part of the annual clinical study report.

Changes of an administrative or logistical nature (changes of address, phone numbers, errata, spelling corrections, among others) must be sent to the EC as an administrative letter for notification only and to ANVISA in the annual clinical study report.

INFORMED CONSENT FORM (ICF)

Informed consent is the agreement of the research participant and/or their legal representative, given freely and withouth mistakes (simulation, fraud or error), dependence, subordination or intimidation, after complete and detailed clarification about the nature of the study, its objectives, methods, expected benefits, potential risks and the inconvenience that it may cause. (CNS RESOLUTION No. 466, OF DECEMBER 12, 2012; CNS RESOLUTION No. 510, OF APRIL 7, 2016). No clinical study procedure should be performed without the research participant having been informed about the clinical trial and having signed and dated the ICF. The ICF previously approved by the EC must be applied to the research participant if he/she is 18 years of age or older and is legally capable, or to the legal guardians (guardian, curator or attorney) with registration in a registry office in the case of a research participant who is legally incapable.
If the research participant and/or his/her legal representative are unable to read and understand, an impartial witness must be present during the consent discussion. After reading and explaining the ICF, if the participant and/or his/her legal representative is unable to provide a written signature, they must give their consent orally and the impartial witness must sign the ICF. The ICF must be applied by the principal investigator or delegated sub-investigator in two copies (one for the research site's files and the other for the research participant), all pages must be initialed and the last page must be dated and signed by all parties involved in the process. The ICF of research participants who are screened and not randomized is also part of the study documentation and, therefore, must be filed and made available for verification by study monitors.

ICF Intended for Pregnant Partners of Study Participants

Pregnant partners will be asked to provide information about their pregnancy, due to having become pregnant while their partner was participating in the above study. Although we know the risks and adverse events of these medications alone (candesartan cilexetil 16mg + chlorthalidone 12.5mg + amlodipine 5mg), we do not know whether this new combination may have any other effects on sperm, pregnancy or the baby, before or after birth. Therefore, collecting information about the pregnancy and the baby's health throughout the pregnancy and up to 1 year after birth is essential to see if anything occurs that may be related to this new proposed fixed-dose combination.

To this purpose, pregnant partners must sign the Informed Commitment Term previously approved by the EC and intended for the purpose set out above. The consent process and control of these documents must follow the same premises described for the ICFs of the participants in this study and described in item 13.4.

RESEARCH PARTICIPANT CONFIDENTIALITY

All research participant data will be identified only by the initials of their name and the code corresponding to their identification in the clinical study, and the participant's personal data (such as name and address) will be omitted throughout the analysis of results, thus maintaining their confidentiality. However, the investigator must allow the clinical study monitor (in the presence or not of the investigator) to review the research participant's records related to the clinical study. This should include all documentation related to the research participant. Although the monitor will have access to the research participant's personal data, this information will be confidential.

The sponsor, study monitors, principal investigator and his/her team will process personal data in accordance with the authorizing hypotheses listed in Law No. 13,709/2018 (General Personal Data Protection Law – "LGPD"), mainly to comply with the legal or regulatory obligation of Libbs (study sponsor), compliance with Good Clinical Practices and other current Brazilian regulations, with the objective of ensuring the protection, confidentiality of data

and well-being of participants and other people involved in this study.
The data of each study participant will only be used for the purpose set out in this protocol and will be kept in a physical or digital file for at least 5 years after the conclusion of the study.

CONFIDENTIAL INFORMATION AND PUBLICATIONS

All information that has not been previously published and provided to the investigator and members of his/her team by the sponsor is considered confidential. This includes, but is not limited to, the investigator's brochure, basic scientific data, clinical study protocol, protocols/amendments (where applicable), clinical records and experimental methods. Any data obtained during the clinical study is also considered confidential. All confidential information is the property of the sponsor and information from this clinical study will be available to government health agencies.

The sponsor recognizes as legitimate the investigator's interest in publishing the results of the study. However, if the publication implies the disclosure of the information and materials referred to in the item above, the Investigator undertakes to forward to the sponsor for approval a proposed text for publication, forty-five (45) days before the scheduled date for delivery to the vehicle that will publish it.
The investigator must disclose the results of the study to the research participants and institutions where the data was obtained.

In compliance with CNS Resolution No. 466 of 2012 and CNS Operational Standard No. 001 of 2013 of the National Health Council, the Ministry of Health and other complementary standards of the EC/CONEP System, the results obtained from this research will be published by the principal investigator, whether favorable or not, with due credit to the researchers and technical staff involved and attached to the Plataforma Brasil in the form of a Research Report. The data will be published in a grouped manner to ensure data confidentiality, participant privacy and anonymity in information transfers, meeting ethical and regulatory requirements.

CARE

Libbs Pharmaceuticals undertakes to provide immediate assistance, as well as to be responsible for the full assistance to research participants with regards to complications and damages arising from the research, as recommended by CNS Res. No. 466/12 and 510/16.

REGULATORY ASPECTS OF CLINICAL RESEARCH

The clinical study will be conducted in Brazil, with the objective of subsidizing, with ANVISA, the registration of the experimental drug and, therefore, it is necessary to obtain the “Single Special Notice for conducting Research in Brazil”. The development of this study is in accordance with the international and national guidelines and regulatory standards for research involving human beings, in particular, the guidelines of the International Conference on Harmonization of Good Clinical Practices (ICH-GCP, 2016), the Good Clinical Practices Guide of the Americas Document (Pan American Health Organization, 2005), CONEP/CNS/MS Resolutions No. 466/2012, operational standard 001/2013, and other applicable current regulations, including guidelines from CONEP in its circular letters, operational standards and normative instructions, as well as compliance with RDC Resolution No. 09, of February 20, 2015 of ANVISA and its updates for conducting clinical trials in Brazil for the purpose of registration.

QUALITY CONTROL AND ASSURANCE

The quality of the clinical study conducted under the sponsorship of Libbs Pharmaceuticals will be ensured in accordance with the specific SOPs of the Clinical Research area, which meet the regulatory determinations of the ICH, Good Clinical Practices (GCPs) and current national standards.

SOURCE DOCUMENTS

Source documents are all original documents in which the first record of the collected data will be made, such as, but not limited to: Medical records; ICF; Laboratory test report; Medication Dispensing and Return Control Form. Source documents must be stored in locked cabinets with restricted access and must be available for inspection by the monitor designated by the sponsor during monitoring visits. All documents related to the clinical study, including, but not limited to, the source documents and physical files of the clinical study, must be archived under the responsibility of the research site for a period of five (05) years from the closing visit of the clinical study. These documents will be subject to audit by

the sponsor or regulatory authority at any time within the above period. In case of archiving outside the research site, the storage location must be informed to the sponsor at the time of the closing visit by means of a formal declaration.

CLINICAL STUDY MONITORING

A designated and trained monitor will oversee the progress of the clinical study, ensuring that it is conducted in accordance with the clinical study protocol, the sponsor's standard operating procedures, GCP and other applicable regulatory requirements. Monitoring visits will be scheduled during the conduct of the study at appropriate intervals, therefore, adequate times and spaces for such visits must be made available by the research site staff. This visit will be scheduled and confirmed in advance by telephone and through the monitoring visit confirmation letter sent by email. During the monitoring visit, the monitor aims to verify:That the rights and well-being of research participants are protected; That the data reported on the clinical study are complete, accurate and verifiable against the source documents; That the clinical study is conducted in accordance with the current clinical protocol, the GCPs and the applicable regulatory requirements.

AUDIT

During or after the clinical study is conducted, the research site may be audited by the sponsor, in a visit independent of the monitoring, with the objective of evaluating the conduct of the clinical study and compliance with the clinical protocol. Compliance with current SOPs, GCPs and applicable regulatory requirements will also be evaluated. This audit must be scheduled in accordance with the current procedure of Libbs Pharmaceuticals or the contracted third- party company.

INDEPENDENT DATA SECURITY MONITORING COMMITTEE

To enhance the safety and integrity of study data, a review committee of independent experts will be convened to periodically review accumulated safety data for the study. Committee meetings will occur at three points throughout the study: Initial meeting to present the protocol: initial meeting of the committee with the study leadership/sponsor for the due approval of the procedures described in the manual, review of the literature on the combination of drugs for the treatment of arterial hypertension, review of the protocol and the informed consent form. Safety Analysis: when 170 participants are included (approximately 25% of the sample) and they complete the 12-week follow-up (85 participants in each group). Safety Analysis: when 340 participants are included (approximately 50% of the sample) and they complete the 12-week follow-up (170 participants in each group). Unscheduled meetings may be requested if any concerns about participant safety arise during the course of the clinical trial (e.g., a series of adverse events attributed to the same event, such as emergency or hypertensive crisis). Further details along with the committee's bylaws that outline its responsibilities and composition are described in the document Data and Safety Committee Manual.

REFERENCES

AGARWAL, Rajiv et al. Chlorthalidone for poorly controlled hypertension in chronic kidney disease: an interventional pilot study. American journal of nephrology, v. 39, n. 2, p. 171-182, 2014.

ALLEMANN, Yves et al. Efficacy of the combination of amlodipine and valsartan in patients with hypertension uncontrolled with previous monotherapy: the Exforge in Failure after Single Therapy (EX-FAST) study. The Journal of Clinical Hypertension, v. 10, n. 3, p. 185-194, 2008.

ALWAN, S.; POLIFKA, J. E.; FRIEDMAN, J. M. Angiotensin II receptor antagonist treatment during pregnancy. Birth Defects Research Part A: Clinical and Molecular Teratology, v. 73, n. 2, p. 123-130, 2005.

ANVISA. Agência Nacional de Vigilância Sanitária. Guia para Registro de Associações em Dose Fixa para o Tratamento da Hipertensão Arterial**.**1ª edição, Brasilia, DF, 2010.

ANVISA. Manual de submissão dos dados de qualidade referente aos produtos sob investigação utilizados em ensaios clínicos: medicamentos sintéticos e semissintéticos, 2019.

ATACAND - bula: comprimido. Responsável técnico Dra. Gisele H. V. C. Teixeira. Cotia: AstraZeneca do Brasil Ltda., 2015. Available at: https://consultas.anvisa.gov.br/#/bulario/q/?nomeProduto=ATACAND. Accessed on: Jan. 19 2022.

BARROSO, Weimar Kunz Sebba et al. Diretrizes Brasileiras de Hipertensão Arterial–2020. Arquivos Brasileiros de Cardiologia, v. 116, p. 516-658, 2021.

BRAMLAGE, Peter; SCHMIDT, Stefanie; SIMS, Helen. Fixed-dose vs free-dose combinations for the management of hypertension—An analysis of 81 958 patients. The Journal of Clinical Hypertension, v. 20, n. 4, p. 705-715, 2018.

BRASIL. MINISTÉRIO DA SAÚDE (MS). CONSELHO NACIONAL DE SAÚDE. Resolução nº 466, de 12 de dezembro de 2012. Diário Oficial da União, 2013.

BRASIL. ANVISA. RESOLUÇÃO DA DIRETORIA COLEGIADA - RDC Nº 38, de 12 de agosto de 2013.

BRASIL. ANVISA RESOLUÇÃO DA DIRETORIA COLEGIADA RDC N° 9, de 20 de fevereiro de 2015.

BRASIL. MINISTÉRIO DA SAÚDE (MS). CONSELHO NACIONAL DE SAÚDE. Resolução n°510, de 07 de abril de 2016.

BRASIL. MINISTÉRIO DA SAÚDE(MS). CONSELHO NACIONAL DE SAÚDE. RESOLUÇÃO DA DIRETORIA COLEGIADA RDC N°251, de 7 de agosto de 1997.

Brochura do Investigador, 2022. Documento 3 do DDCM.

BULSARA, K.G.; CASSAGNOL, M. Amlodipine. 8 ago 2021. In: StatPearls [Internet]. Treasure Island (FL): StatPearls Publishing; 2021. Available at: <https://www.ncbi.nlm.nih.gov/books/NBK519508/>

CALHOUN, David A. et al. Triple antihypertensive therapy with amlodipine, valsartan, and hydrochlorothiazide: a randomized clinical trial. Hypertension, v. 54, n. 1, p. 32-39, 2009.

CAREY, Robert M.; WHELTON, Paul K. Prevention, detection, evaluation, and management of high blood pressure in adults: synopsis of the 2017 American College of Cardiology/American Heart Association Hypertension Guideline. Annals of internal medicine, v. 168, n. 5, p. 351-358, 2018.

CHENG, Yan-Qiong et al. Synergism of amlodipine and candesartan on blood pressure reduction and organ protection in hypertensive rats. Clinical and Experimental Pharmacology and Physiology, v. 45, n. 6, p. 514-524, 2018.

CHOW, Clara K et al. QUARTET Investigators. Initial treatment with a single pill containing quadruple combination of quarter doses of blood pressure medicines versus standard dose monotherapy in patients with hypertension (QUARTET): a phase 3, randomised, double-blind, active-controlled trial. Lancet, v. 398, n. 10305, p. 1043-1052, 2021.

CLORANA – bula (hidroclorotiazida): Comprimido. Sanofi Medley Pharmaceuticals. Sanofi Medley Pharmaceuticals. Available at: https://consultas.anvisa.gov.br/#/bulario/q/?nomeProduto=clorana

CLORTALIDONA - bula: comprimido. Responsável técnico Dr. Ronoel Caza de Dio. Hortolândia - SP: EMS S/A., 2021. Available at: https://consultas.anvisa.gov.br/#/bulario/q/?nomeProduto=clortalidona. Accessed on: Jan. 19 2022.

CHRYSANT, Steven G et al. Triple-Combination therapy with olmesartan, amlodipine, and hydrochlorothiazide in black and non-black study participants with hypertension: the TRINITY randomized, double-blind, 12-week, parallel- group study. American journal of cardiovascular drugs : drugs, devices, and other interventions, vol. 12, n.4, p. 233-243, 2012.

DE LIMA BRAZ, Cyntia et al. Medicamentos com atividade sobre o citocromo P450 utilizados por idosos em domicílio. Rev Med Minas Gerais, v. 2018, n. 28, 1927.

DE ZEEUW, D.; REMUZZI, G.; KIRCH, W. Pharmacokinetics of candesartan cilexetil in patients with renal or hepatic impairment. Journal of human hypertension, v. 11, 1997.

Diovan – bula (valsartana): Comprimidos. Novartis Biociências AS. Farm. Resp.: Flavia Regina Pegorer. Available at: https://consultas.anvisa.gov.br/#/bulario/q/?nomeProduto=Diovan

DRUGBANK. Clortalidona. 20 Sep 2021. Available at: <https://go.drugbank.com/drugs/DB00310> Accessed on: 25 Sep 2021.

DRUGBANK. Besilato de anlodipino. 21 Sep 2021. Available at: <https://go.drugbank.com/drugs/DB00381> Acesso em: 27 Sep 2021.

EGAN, Brent M. et al. Initial monotherapy and combination therapy and hypertension control the first year. Hypertension, v. 59, n. 6, p. 1124-1131, 2012. GAVRAS, Haralambos. Update on the clinical pharmacology of candesartan cilexetil. American journal of hypertension, v. 13, n. S1, p. 25S-30S, 2000.

Exforge HTC - bula (valsartana + hidroclorotiazida + besilato de Anlodipino): comprimidos. Novartis Biociências SA. Farm. Resp.: Flavia Regina Pegorer. Available at: https://consultas.anvisa.gov.br/#/bulario/q/?nomeProduto=Exforge

FERDINAND, Keith C et al. Efficacy and safety of aliskiren-based dual and triple combination therapies in US minority patients with stage 2 hypertension. Journal of the American Society of Hypertension: JASH, v. 5, n. 2, p. 102-13, 2011.

FLYNN, Joseph T. et al. Clinical practice guideline for screening and management of high blood pressure in children and adolescents. Pediatrics, v. 140, n. 3, 2017.

FOROUZANFAR, Mohammad H. et al. Global burden of hypertension and systolic blood pressure of at least 110 to 115 mm Hg, 1990-2015. Jama, v. 317, n. 2, p. 165-182, 2017.

GLEITER, Christoph H.; MÖRIKE, Klaus E. Clinical pharmacokinetics of candesartan. Clinical pharmacokinetics, v. 41, n. 1, p. 7-17, 2002.

GRADMAN, Alan H. Rationale for triple-combination therapy for management of high blood pressure. The Journal of Clinical Hypertension, v. 12, n. 11, p. 869- 878, 2010.

Hernán, Miguel A., and James M. Robins. "Per-protocol analyses of pragmatic trials." N Engl J Med 377.14 (2017): 1391-1398.

HIGAKI, Jitsuo et al. The efficacy and long-term safety of a triple combination of 80 mg telmisartan, 5 mg amlodipine and 12.5 mg hydrochlorothiazide in Japanese patients with essential hypertension: a randomized, double-blind study with open-label extension. Hypertension research: official journal of the Japanese Society of Hypertension, v. 40, n. 1, p. 51-60, 2017.

HUSAIN, Asif et al. A review on candesartan: pharmacological and pharmaceutical profile. Journal of Applied Pharmaceutical Science, v. 1, n. 10, p. 12-17, 2011.

IINO, Yasuhiko et al. Renoprotective effect of losartan in comparison to amlodipine in patients with chronic kidney

disease and hypertension—a report of the Japanese Losartan Therapy Intended for the Global Renal Protection in Hypertensive Patients (JLIGHT) study. Hypertension Research, v. 27, n. 1, p. 21- 30, 2004.

INTERNATIONAL PEER REVIEWED CHEMICAL SAFETY INFORMATION (INCHEM)_chlortalidone, 1992. Available at: https://inchem.org/documents/pims/pharm/chlortha.htm. Accessed in: October 2021.

JAMERSON, Kenneth A. et al. Rationale and design of the avoiding cardiovascular events through combination therapy in patients living with systolic hypertension (ACCOMPLISH) trial: the first randomized controlled trial to compare the clinical outcome effects of first-line combination therapies in hypertension. American journal of hypertension, v. 17, n. 9, p. 793-801, 2004.

JOHNSTON, Colin I.; RISVANIS, John. Preclinical pharmacology of angiotensin II receptor antagonists: update and outstanding issues. American journal of hypertension, v. 10, n. S9, p. 306S-310S, 1997.

JOHNSTON, Atholl; STAFYLAS, Panagiotis; STERGIOU, George S. Effectiveness, safety and cost of drug substitution in hypertension. British journal of clinical pharmacology, v. 70, n. 3, p. 320-334, 2010.

KERNDT, C.C.; PATEL, J. Chlorthalidone. May 17, 2021. StatPearls [Internet]. Treasure Island (FL): StatPearls Publishing; Available at: <https://www.ncbi.nlm.nih.gov/books/NBK553174/> Accessed on: 21 Sep 2021.

LASSERSON, Daniel S et al. How quickly should we titrate antihypertensive medication? Systematic review modelling blood pressure response from trial data. Heart, v. 97, n.21, p. 1771-5, 2011.

LAW, MR MORRIS, J. K.; WALD, N. J. Use of blood pressure lowering drugs in the prevention of cardiovascular disease: meta-analysis of 147 randomised trials in the context of expectations from prospective epidemiological studies. Bmj, v. 338, 2009.

LAW, M. R. et al. Value of low dose combination treatment with blood pressure lowering drugs: analysis of 354 randomised trials. Bmj, v. 326, n. 7404, p. 1427, 2003

LEE, Dae Wook et al. Systematic Review with Network Meta-Analysis: Comparative Efficacy and Safety of Combination Therapy with Angiotensin II Receptor Blockers and Amlodipine in Asian Hypertensive Patients. International journal of hypertension, v. 2019, 2019.

LEE, Hae Won et al. Pharmacokinetics and bioequivalence of fixed-dose combination of candesartan cilexetil/amlodipine besylate (16/10 mg) versus coadministration of individual formulations in healthy subjects. Translational and Clinical Pharmacology, v. 28, n. 2, p. 92, 2020.

LIANG, Wenjing et al. Comparison of thiazide-like diuretics versus thiazide-type diuretics: a meta-analysis. Journal of cellular and molecular medicine, v. 21, n. 11, p. 2634-2642, 2017.

LUND, Lars H. et al. Heart failure with mid-range ejection fraction in CHARM: characteristics, outcomes and effect of candesartan across the entire ejection fraction spectrum. European journal of heart failure, v. 20, n. 8, p. 1230-1239, 2018.

MACDONALD, Thomas M et al. British Hypertension Society Programme of Prevention And Treatment of Hypertension With Algorithm - based Therapy (PATHWAY). Combination Therapy Is Superior to Sequential Monotherapy for the Initial Treatment of Hypertension: A Double-Blind Randomized Controlled Trial. J Am Heart Assoc, v. 6, n. 11, p. e006986, 2017.

MALACHIAS, Marcus Vinicius Bolivar et al. 7ª Diretriz Brasileira de Hipertensão Arterial: Capítulo 1-Conceituação, Epidemiologia e Prevenção Primária. Arquivos Brasileiros de Cardiologia, v. 107, p. 1-6, 2016.

MALADKAR, Manish et al. Triple drug combination of telmisartan, amlodipine and hydrochlorothiazide in the treatment of essential hypertension. Open Journal of Internal Medicine, v.2, n. 2, p. 67-71, 2012.

MALTA, Deborah Carvalho et al. Prevalence of high blood pressure measured in the Brazilian population, National Health Survey, 2013. Sao Paulo Medical Journal, v. 134, p. 163-170, 2016.

MALTA, Deborah Carvalho et al. Prevalência da hipertensão arterial segundo diferentes critérios diagnósticos, Pesquisa

Nacional de Saúde. Revista Brasileira de Epidemiologia, v. 21, p. e180021, 2018.

MANCIA, Giuseppe et al. Two-drug combinations as first-step antihypertensive treatment. Circulation research, v. 124, n. 7, p. 1113-1123, 2019.

MARTIN, Seth S et al. Comparison of a Novel Method vs the Friedewald Equation for Estimating Low-Density Lipoprotein Cholesterol Levels From the Standard Lipid Profile. JAMA, p. 2061-2068.

MCLEAN, Gary et al. Digital interventions to promote self-management in adults with hypertension systematic review and meta-analysis. Journal of hypertension, v. 34, n. 4, p. 600, 2016.

MCCLELLAN, Karen J.; GOA, Karen L. Candesartan cilexetil. Drugs, v. 56, n. 5, p. 847-869, 1998

MICROMEDEX PHARMACEUTICAL KNOWLEDGE_DRUGDEX_ANLODIPINO. Available at: https://www.micromedexsolutions.com/micromedex2/librarian/CS/035EF6/ND_PR/evidencexpert/ND_P/evidencexpert/DUPLICATIONSHIELDSYNC/66E05A/ND_PG/evidencexpert/ND_B/evidencexpert/ND_AppProduct/evidencexpert/ND_T/evidencexpert/PFActionId/pf.HomePage?navitem=topHome&isToolPage=true Acesso em: novembro 2021.

MICROMEDEX PHARMACEUTICAL KNOWLEDGE_DRUGDEX_CANDESARTANA CILEXETILA. Available at: https://www.micromedexsolutions.com/micromedex2/librarian/CS/035EF6/ND_PR/evidencexpert/ND_P/evidencexpert/DUPLICATIONSHIELDSYNC/66E05A/ND_PG/evidencexpert/ND_B/evidencexpert/ND_AppProduct/evidencexpert/ND_T/evidencexpert/PFActionId/pf.HomePage?navitem=topHome&isToolPage=true. Accessed in: November 2021.

MORAGA, Paula et al. Global, regional, and national age-sex specific mortality for 264 causes of death, 1980-2016: a systematic analysis for the Global Burden of Disease Study 2016. The Lancet, v. 390, n. 10100, p. 1151-1210, 2017.

MORGAN, Jamie L. et al. Pharmacokinetics of amlodipine besylate at delivery and during lactation. Pregnancy hypertension, v. 11, p. 77-80, 2018.

Mo, Yin, et al. "Non-adherence in non-inferiority trials: pitfalls and recommendations." bmj 370 (2020).

MUSINI, Vijaya M. et al. Blood pressure-lowering efficacy of monotherapy with thiazide diuretics for primary hypertension. Cochrane Database of Systematic Reviews, n. 5, 2014.

NATIONAL HIGH BLOOD PRESSURE EDUCATION PROGRAM et al. The seventh report of the Joint National Committee on prevention, detection, evaluation, and treatment of high blood pressure. 2004.

NAKAJIMA, Tadashi *et al.* Comparative Effectiveness of Angiotensin II Receptor Blockers in Patients with Hypertension in Japan — Systematic Review and Network Meta-Analysis —. Circulation Reports, v. 2, n. 10, p. 576-586, 2020.

NILSON, Eduardo Augusto Fernandes et al. Custos atribuíveis a obesidade, hipertensão e diabetes no Sistema Único de Saúde, Brasil, 2018. Revista Panamericana de Salud Pública, v. 44, p. e32, 2020.

NORVASC - bula: comprimido. Responsável técnico Adriana L. N. Heloany. Itapevi - SP: Laboratórios Pfizer Ltda, 2021. Available at: https://consultas.anvisa.gov.br/#/bulario/q/?nomeProduto=Norvasc. Accessed on: Jan. 19 2022.

OLIVEIRA, Gláucia Maria Moraes de et al. 2017 guidelines for arterial hypertension management in primary health care in Portuguese language countries. Arquivos brasileiros de cardiologia, v. 109, p. 389-396, 2017.

OLIVEIRA, Maria Hebe Nóbrega de et al. 6. Fármacos cardiovasculares na gestação e amamentação. Arquivos Brasileiros de Cardiologia, v. 93, p. 120-126, 2009.

ORGANISATION FOR ECONOMIC CO-OPERATION AND DEVELOPMENT (OECD). OECD GUIDELINE FOR TESTING OF CHEMICALS. Repeated dose 90-day oral toxicity study in rodents. Guidance 408, 2018a. Available at: https://www.oecd-ilibrary.org/docserver/9789264070707-en.pdf?expires=1633520510&id=id&accname=guest&checksum=E63865002C2 DDCA9126C29D27C3B4B74 .

PASTORE, C. A. et al. Diretrizes da Sociedade Brasileira de Cardiologia sobre análise e emissão de laudos eletrocardiográficos. Arquivos Brasileiros de Cardiologia, v. 106, n. 4, p. 1-23, 2016.

PICON, Rafael V. et al. Trends in prevalence of hypertension in Brazil: a systematic review with meta-analysis. PLOS one, v. 7, n. 10, p. e48255, 2012.

PÓVOA, Fernando Focaccia; PÓVOA, Rui. EXISTEM DIFERENÇAS ENTRE OS DIURÉTICOS TIAZÍDICOS?. Rev Bras Hipertens, v. 27, n. 3, p. 103-5, 2020.

PÓVOA, Rui et al. I brazilian position paper on antihypertensive drug combination. Arquivos brasileiros de cardiologia, v. 102, p. 203-210, 2014.

PUBCHEM. Bethesda (MD): National Library of Medicine (US), National Center for Biotechnology Information; 2004. PubChem Compound Summary for CID 2540, Candesartan cilexetil; Available at: <https://pubchem.ncbi.nlm.nih.gov/compound/Candesartan-cilexetil>

PUBCHEM. Bethesda (MD): National Library of Medicine (US), National Center for Biotechnology Information; 2004. PubChem Compound Summary for CID 2541, Candesartan; Available at: <https://pubchem.ncbi.nlm.nih.gov/compound/Candesartan>

PUBCHEM. Bethesda (MD): National Library of Medicine (US), National Center for Biotechnology Information; 2004. PubChem Compound Summary for CID 2732, Chlorthalidone. Available at: <https://pubchem.ncbi.nlm.nih.gov/compound/Chlorthalidone>

PUBCHEM. Bethesda (MD): National Library of Medicine (US), National Center for Biotechnology Information; 2004. PubChem Compound Summary for CID 60496, Amlodipine besylate. Available at: <https://pubchem.ncbi.nlm.nih.gov/compound/Amlodipine-besylate>Accessed on September 27, 2021.

QUEIROZ, Mônica Estuque Garcia; FOZ, Mary Lee Faria Norris Nelsen. Atenção integrada a pessoa com insuficiência cardíaca na perspectiva terapêutico- ocupacional e psicológica: um relato de experiência. Revista Família, Ciclos de Vida e Saúde no Contexto Social, v. 6, n. 1, p. 123-129, 2018.

ROUSH, George et al. Chlorthalidone Compared With Hydrochlorothiazide in Reducing Cardiovascular Events.Hypertension, p. 1110-1117; 2012.

SABATÉ, Eduardo et al. (Ed.). Adherence to long-term therapies: evidence for action. World Health Organization, 2003.

SALAM, Abdul et al. TRIple pill vs usual care management for patients with mild- to-moderate hypertension (TRIUMPH): study protocol. American heart journal, v. 167, n. 2, p. 127-132, 2014.

SARUTA, Takao et al. Effects of candesartan and amlodipine on cardiovascular events in hypertensive patients with chronic kidney disease: subanalysis of the CASE-J Study. Hypertension Research, v. 32, n. 6, p. 505-512, 2009.

Smith, T. R., et al. "Combination therapy with amlodipine/valsartan in essential hypertension: a 52-week, randomised, open-label, extension study." International journal of clinical practice 64.10 (2010): 1367-1374.

SOHN, Il Suk et al. Efficacy and tolerability of combination therapy versus monotherapy with candesartan and/or amlodipine for dose finding in essential hypertension: a phase II multicenter, randomized, double-blind clinical trial. Clinical therapeutics, v. 39, n. 8, p. 1628-1638, 2017.

SCENTRYPHAR – PESQUISA CLÍNICA LTDA. Estudo de Interação Farmacocinética de besilato de anlodipino administrada isoladamente em comparação com a administração concomitantes de candesartana cilexetila, clortalidona e besilato de anlodipino, em participantes da pesquisa sadios, sendo as formulações a serem empregadas no estudo: Atacand®, comprimido de 16 mg contendo candesartana cilexetila, comercializado pela AstraZeneca do Brasil Ltda.; Clortalidona comprimido de 12,5 mg, comercializada pela EMS S.A.; e Norvasc® comprimido de 5 mg de besilato de anlodipino, comercializado pelo Laboratórios Pfizer Ltdastph. Código 04/20. Apr 24, 2021. Relatório interno. Libbs Pharmaceuticals.

SCENTRYPHAR – PESQUISA CLÍNICA LTDA. Estudo de Interação Farmacocinética de clortalidona administrada isoladamente em comparação com a administração concomitantes de candesartana cilexetila, clortalidona e besilato de anlodipino, em participantes da pesquisa sadios, sendo as formulações a serem empregadas no estudo: Atacand®, comprimido de 16 mg contendo candesartana cilexetila, comercializado pela AstraZeneca do Brasil Ltda.; Clortalidona comprimido de 12,5 mg, comercializada pela EMS S.A.; e Norvasc® comprimido de 5 mg de besilato de anlodipino, comercializado pelo Laboratórios Pfizer Ltdastph. Código 03/20. Apr 17, 2021. Relatório interno. Libbs Pharmaceuticals.

SCENTRYPHAR – PESQUISA CLÍNICA LTDA. Estudo de Interação Farmacocinética de candesartana cilexetila administrada isoladamente em comparação com a administração concomitantes de candesartana cilexetila, clortalidona e besilato de anlodipino, em participantes da pesquisa sadios, sendo as formulações a serem empregadas no estudo: Atacand®, comprimido de 16 mg contendo candesartana cilexetila, comercializado pela AstraZeneca do Brasil Ltda.; Clortalidona comprimido de 12,5 mg, comercializada pela EMS S.A.; e Norvasc® comprimido de 5 mg de besilato de anlodipino, comercializado pelo Laboratórios Pfizer Ltdastph. Código 02/20. Apr 12, 2021. Relatório interno. Libbs Pharmaceuticals.

TAKAI, Shinji et al. Candesartan and amlodipine combination therapy provides powerful vascular protection in stroke-prone spontaneously hypertensive rats. Hypertension Research, v. 34, n. 2, p. 245-252, 2011.

UNGER, Thomas et al. 2020 International Society of Hypertension global hypertension practice guidelines. Hypertension, v. 75, n. 6, p. 1334-1357, 2020.

VOLPE, Massimo; GALLO, Giovanna; TOCCI, Giuliano. New approach to blood pressure control: triple combination pill. Trends in cardiovascular medicine, v. 30, n. 2, p. 72-77, 2020.

VI Diretrizes Brasileiras de Hipertensão. JORNAL, S. B. C. et al. VI Diretrizes Brasileiras de Hipertensão. 2010.

WEBSTER, Ruth et al. Fixed low-dose triple combination antihypertensive medication vs usual care for blood pressure control in patients with mild to moderate hypertension in Sri Lanka: a randomized clinical trial. Jama, v. 320, n. 6, p. 566-579, 2018.

WILLIAMS, Bryan et al. 2018 ESC/ESH Guidelines for the management of arterial hypertension: The Task Force for the management of arterial hypertension of the European Society of Cardiology (ESC) and the European Society of Hypertension (ESH). European heart journal, v. 39, n. 33, p. 3021- 3104, 2018.

WANG, Nelson et al. Association of low-dose triple combination therapy with therapeutic inertia and prescribing patterns in patients with hypertension: a secondary analysis of the TRIUMPH trial. JAMA cardiology, v. 5, n. 11, p. 1219- 1226, 2020a.

WORLD HEALTH ORGANIZATION et al. Causes of Death 2008 [online database]. Geneva. World Health Organization, 2008.

WORLD HEALTH ORGANIZATION (WHO). Hypertension, 2015. Available at: https://www.who.int/health-topics/hypertension#tab=tab_1. Accessed on: September 23, 2021.

WORLD HEALTH ORGANIZATION. (2018). Noncommunicable diseases country profiles 2018. World Health Organization.

ZANCHETTI, Alberto. Consenso Latinoamericano sobre hipertensión arterial. Journal of hypertension, v. 6, n. 2, p. 1-28, 2001.

ZHOU, Bin et al. Worldwide trends in hypertension prevalence and progress in treatment and control from 1990 to 2019: a pooled .

ANNEXES

ANNEX I – NEW YORK HEART ASSOCIATION (NYHA) CLASSIFICATION

New York Heart Association (NYHA) Classification	
I	Lack of symptoms (dyspnea) during daily activities
II	Mild symptoms during daily activities
III	Symptoms triggered by less intense activities or minor exertion
IV	Symptoms with minimal effort or at rest

Source: Adapted from Queiroz, 2018.

ANNEX II – PSI - EXFORGE HCT® PACKAGE LEAFLET

Exforge HCT®

(valsartan + hydrochlorothiazide + anlodipine besylate)

Novartis Biociências SA

Coated Tablets

160/12,5/5 mg, 160/12,5/10 mg, 160/25/5 mg, 160/25/10 mg ou 320/25/10 mg

Professional Package Insert

NOVARTIS

EXFORGE HCT®
valsartan + hydrochlorothiazide + anlodipine besylate

PRESENTATIONS
Exforge HCT® 160/12.5/5 mg or 320/25/10 mg: packs containing 14 or 28 coated tablets.
Exforge HCT® 160/12.5/10 mg, 160/25/5 mg or 160/25/10 mg: packs containing 28 coated tablets.

ORAL ROUTE
ADULT USE

COMPOSITION
Each coated tablet of Exforge HCT® 160/12.5/5 mg contains 160 mg of valsartan, 12.5 mg of hydrochlorothiazide and 6.94 mg of anlodipine besylate (equivalent to 5 mg of anlodipine).

Each coated tablet of Exforge HCT® 160/12.5/10 mg contains 160 mg of valsartan, 12.5 mg of hydrochlorothiazide and 13.87 mg of anlodipine besylate (equivalent to 10 mg of anlodipine).

Each coated tablet of Exforge HCT® 160/25/5 mg contains 160 mg of valsartan, 25 mg of hydrochlorothiazide and 6.94 mg of anlodipine besylate (equivalent to 5 mg of anlodipine).

Each coated tablet of Exforge HCT® 160/25/10 mg contains 160 mg of valsartan, 25 mg of hydrochlorothiazide and 13.87 mg of anlodipine besylate (equivalent to 10 mg of anlodipine).

Each coated tablet of Exforge HCT® 320/25/10 mg contains 320 mg of valsartan, 25 mg of hydrochlorothiazide and 13.87 mg of anlodipine besylate (equivalent to 10 mg of anlodipine).

Excipients: microcrystalline cellulose, crospovidone, silicon dioxide, magnesium stearate, hypromellose, macrogol, talc, titanium dioxide (only the 160/12.5/5 mg, 160/12.5/10 mg and 160/25/5 mg tablets), red iron oxide (only the 160/12.5/10 mg tablets), yellow iron oxide (except the 160/12.5/5 mg tablets).

TECHNICAL INFORMATION FOR HEALTHCARE PROFESSIONALS

1. INDICATIONS
Exforge HCT® is indicated for the treatment of essential hypertension.
The fixed combination medication is not indicated as initial therapy in hypertension (see "Dosage and instructions for use").

2. EFFICACY RESULTS
Exforge HCT® was evaluated in a double-blind, active, controlled study in hypertensive patients. A total of 2,271 patients with moderate to severe hypertension (mean initial systolic/diastolic pressure of 170/107 mmHg) received treatment with anlodipine/valsartan/HCT 10/320/25 mg, valsartan/HCT 320/25 mg, anlodipine/valsartan 10/320 mg or HCT/anlodipine 25/10 mg. At the start of the study, patients received lower doses of their combination treatment and were titrated up to their full dose by week 2. A total of 55% of the patients were men, 14% over 65 years of age, 72% Caucasian and 17% black.
At week 8, the reductions in mean systolic/diastolic blood pressure were 39.7/24.7 mmHg with Exforge HCT® (n=571), 32.0/19.7 mmHg with valsartan/HCT (n=553), 33.5/21.5 mmHg with anlodipine/valsartan (n=558) and 31.5/19.5 mmHg with anlodipine/HCT (n=554). Triple combination therapy was statistically superior to each of the three double combinations in reducing diastolic and systolic blood pressure. The reductions in systolic/diastolic pressure with Exforge HCT® were 7.6/5.0 mmHg greater than with valsartan/HCT, 6.2/3.3 mmHg greater than with anlodipine/valsartan and 8.2/5.3 mmHg greater than with anlodipine/HCT. The full effect of reducing blood pressure was achieved within two weeks of treatment at the maximum dose of Exforge HCT®. A statistically significant proportion of patients achieved pressure control (< 140/90 mmHg) with Exforge HCT® (71%) compared to each of the three dual therapies combined (45-54%).
A subgroup of 268 patients was evaluated with ambulatory blood pressure monitoring. Within 24 hours, clinically and statistically superior reductions in systolic and diastolic blood pressure were observed with the triple combination when compared to valsartan/HCT, valsartan/anlodipine and HCT/anlodipine[1]

In a double-blind controlled study, age, gender and race did not significantly influence the response to Exforge HCT®.

References
1. An 8-week, multicenter, randomized, double-blind, parallel-group study to evaluate the efficacy and safety of the combination of valsartan/HCTZ/amlodipine compared to valsartan/HCTZ, valsartan/amlodipine, and HCTZ/amlodipine in patients with moderate to severe hypertension. A2302. Novartis Pharma AG. 1-May-08 [54].

3. PHARMACOLOGICAL CHARACTERISTICS
Pharmacotherapeutic group: angiotensin II antagonist (valsartan), combinations with dihydropyridine derivatives (anlodipine) and thiazide diuretics (hydrochlorothiazide), ATC code: C09DX01.

Pharmacodynamics
Exforge HCT® is a combination of three antihypertensive compounds with complementary blood pressure control mechanisms in patients with hypertension: anlodipine belongs to the class of calcium channel blockers, valsartan to the class of angiotensin II (Ang II) antagonists and hydrochlorothiazide to the class of thiazide diuretics. The combination of these three substances has an additive antihypertensive effect, reducing blood pressure to a greater extent than when compared to the components alone.

anlodipine
The anlodipine in Exforge HCT® inhibits the transmembrane influx of calcium ions into vascular and cardiac smooth muscle. The mechanism of antihypertensive action is due to the direct relaxing effect on vascular smooth muscle, which causes a reduction in peripheral vascular resistance and blood pressure. Experimental data suggest that anlodipine binds to both dihydropyridine and non-dihydropyridine binding sites. The process of vascular and cardiac smooth muscle contraction depends on the entry of extracellular calcium ions into these cells via specific ion channels.

After administering therapeutic doses to patients with hypertension, anlodipine produces vasodilation, which results in a reduction in blood pressure in the supine or upright position. In chronic doses, these reductions in blood pressure are not accompanied by a significant change in heart rate or plasma levels of catecholamines.

There is a correlation between plasma concentrations and effect in both young and elderly patients.

In hypertensive patients with normal renal function, therapeutic doses of anlodipine result in a reduction in renal vascular resistance and an increase in glomerular filtration rate and effective renal plasma flow with no change in filtration fraction or proteinuria.

As with other calcium channel blockers, hemodynamic measurements of cardiac function when at rest and during exercise or physical activity in patients with normal ventricular function treated with anlodipine have generally shown a small increase in cardiac index with no significant influence on dP/dt or left ventricular end-diastolic pressure and volume. In hemodynamic studies, anlodipine was not associated with a negative inotropic effect when administered at the therapeutic dose range in healthy animals and humans, even when coadministered with beta-blockers in humans.

Anlodipine does not change sinoatrial node function or atrioventricular conduction in healthy animals and humans. In clinical studies in which anlodipine was administered in combination with beta-blockers in patients with hypertension or angina, no adverse effects on electrocardiographic parameters were observed.

Anlodipine has been shown to have beneficial clinical effects in patients with chronic stable angina, vasospastic angina and angiographically documented coronary artery disease.

valsartan
Valsartan is a potent and specific angiotensin II receptor antagonist, active orally. It acts selectively on the AT_1 subtype receptor, which is responsible for the well-known actions of angiotensin II. Elevated plasma levels of Ang II following AT_1 receptor blockade with valsartan may stimulate the unblocked AT_2 receptor, which apparently counterbalances the effect of the AT_1 receptor. Valsartan has no partial agonist activity on AT_1 receptors and has a much higher affinity (around 20,000 times) for AT_1 receptors than for AT_2 receptors.

Valsartan does not inhibit ACE, also known as kininase II, which converts angiotensin I into angiotensin II and degrades bradykinin. Given that angiotensin II antagonists have no effect on ACE and do not potentiate the effects of bradykinin or substance P, they are unlikely to be associated with cough. In clinical studies in which valsartan was compared with ACE inhibitors, the incidence of dry cough was significantly lower ($p < 0.05$) in patients treated with valsartan than in those treated with ACE inhibitors (2.6% versus 7.9%, respectively). In a clinical study of patients with a history of dry cough during therapy with ACE inhibitors, 19.5% of patients receiving valsartan and 19.0% of those receiving a thiazide diuretic had episodes of cough, compared to 68.5% of those treated with ACE inhibitors ($p < 0.05$). Valsartan does not bind to or block other hormone receptors or ion channels important in cardiovascular regulation.

The administration of valsartan to patients with hypertension reduces blood pressure without affecting heart rate. In most patients, after administration of a single oral dose, the onset of antihypertensive activity occurs within two hours and the peak reduction in blood pressure is reached in 4-6 hours. The antihypertensive effect persists for 24 hours after administration. During repeated administrations, the maximum reduction in blood pressure with any dose is usually achieved in 2-4 weeks and is maintained during long-term therapy.

Abrupt discontinuation of valsartan is not associated with rebound hypertension or other clinical adverse effects. Valsartan has been shown to significantly reduce hospitalizations in patients with chronic heart failure (NYHA classes II-IV). The benefits were greater in patients who were not receiving ACE inhibitors or beta-blockers. Valsartan also reduced cardiovascular mortality in clinically stable patients with heart failure or left ventricular dysfunction after myocardial infarction.

hydrochlorothiazide

The site of action of thiazide diuretics is mainly in the distal convoluted tubule of the kidneys. It has been shown that there is a high affinity for receptors in the renal cortex, as the main binding site for the action of thiazide diuretics by inhibiting NaCl transport in the distal convoluted tubule. The mechanism of action of thiazide diuretics is the inhibition of Na^{+} and Cl- ion transport, probably through competition for the Cl- binding site, which affects electrolyte reabsorption mechanisms in the kidneys. Thus, there is an increased excretion of sodium and chlorine in approximately equal quantities. Indirectly, the diuretic action reduces plasma volume, with a consequent increase in plasma renin activity, increased aldosterone secretion, leading to an increase in urinary potassium loss and a reduction in plasma potassium.

Non-melanoma skin cancer (NMSC)

Based on the data available from epidemiological studies, a cumulative dose-dependent association was observed between HCTZ and NMSC. One study included a population comprising 71,533 cases of basal cell carcinoma (BCC) and 8,629 cases of squamous cell carcinoma (SCC) matched to 1,430,833 and 172,462 population controls, respectively. High HCTZ use (≥50,000 mg cumulative) was associated with an adjusted odds ratio (OR) of 1.29 (95% CI: 1.23-1.35) for BCC and 3.98 (95% CI: 3.68-4.31) for BCC. A clear cumulative dose-response relationship was observed for both BCC and SCC. Another study showed a possible association between lip cancer (SCC) and exposure to HCTZ: 633 cases of lip cancer were matched with 63,067 population controls using a risk sampling strategy. A clear cumulative dose-response relationship was demonstrated with an adjusted OR 2.1 (95% CI: 1.7-2.6) increasing to OR 3.9 (3.0-4.9) for high utilization (~ 25,000 mg) and OR 7.7 (5.7-10.5) for the highest cumulative dose (~ 100,000 mg). For example: A cumulative dose of 100,000 mg corresponds to more than 10 years of daily use with a defined daily dose of 25 mg (see "Warnings and Precautions" and "Adverse Reactions").

Pharmacokinetics

Linearity

Anlodipine, valsartan and hydrochlorothiazide have linear pharmacokinetics.

anlodipine

- Absorption

After oral administration of therapeutic doses of anlodipine alone, peak plasma concentrations are reached between 6 and 12 hours. Absolute bioavailability has been estimated at between 64% and 80%. Bioavailability is not altered by food intake.

- Distribution
The volume of distribution is approximately 21 L/Kg. In vitro studies with anlodipine have shown that approximately 97.5% of the circulating drug is bound to proteins. Anlodipine crosses the placental barrier and is excreted in breast milk.

- Biotransformation/ Metabolism
Anlodipine is extensively metabolized in the liver (approximately 90%) into inactive metabolites.

- Elimination
The elimination of anlodipine from plasma is biphasic with a terminal elimination half-life of around 30 to 50 hours. Steady-state plasma levels are obtained after 7-8 days of consecutive doses. 10% of unchanged anlodipine and 60% of anlodipine metabolites are excreted in the urine.

valsartan
- Absorption
After oral administration of valsartan alone, peak plasma concentrations are reached in 2-4 hours. The average absolute bioavailability for valsartan is 23%. Food reduces exposure to valsartan (measured by ASC) by around 40% and peak plasma concentration (C_{max}) by around 50%, although around 8 hours after administration, plasma concentrations of valsartan are similar in patients who have taken the product on an empty stomach or with food. The reduction in ASC, however, is not accompanied by a clinically significant reduction in therapeutic effects, and valsartan can therefore be used with or without food.

- Distribution
The steady-state volume of distribution of valsartan after intravenous administration is around 17 liters, indicating that valsartan is not widely distributed to the tissues. Valsartan has a high binding rate to serum proteins (94 – 97%), mainly to serum albumin.

- Biotransformation/Metabolism
Valsartan is not extensively transformed, with only 20% of the dose being recovered as metabolites. A pharmacologically inactive hydroxy metabolite has been identified in plasma at low concentrations (less than 10% of valsartan ASC).

- Elimination
Valsartan has a multiexponential kinetic decay ($t_{1/2}\alpha < 1$h and $t_{1/2}\beta$ around 9 h). Valsartan is mainly eliminated via the feces (around 83% of the dose) and urine (around 13% of the dose) in its unchanged form. After intravenous administration, valsartan's plasma clearance is around 2 L/h and its renal clearance is 0.62 L/h (around 30% of total clearance). The half-life of valsartan is 6 hours.

hydrochlorothiazide
- Absorption
The absorption of hydrochlorothiazide after an oral dose is rapid (t_{max} around 2 hours). The increase in mean ASC is linear and dose-proportional over the therapeutic range. Concomitant administration with food can either decrease or increase the systemic availability of hydrochlorothiazide, compared to fasting administration. The magnitude of this effect is small and of little clinical importance. The absolute bioavailability of hydrochlorothiazide is 70% after oral administration.

- Distribution
The distribution and elimination kinetics are generally described by a biexponential decay function. The apparent volume of distribution is 4-8 L/Kg. Circulating hydrochlorothiazide is bound to serum proteins (40-70%), mainly serum albumin. Hydrochlorothiazide also accumulates in erythrocytes at approximately 3 times the level in plasma.

- Biotransformation/ Metabolism
Hydrochlorothiazide is eliminated predominantly as unchanged drug.

- Elimination
Hydrochlorothiazide is eliminated from plasma with an average half-life of 6 to 15 hours in the final elimination phase. There are no changes in the kinetics of hydrochlorothiazide with repeated administrations and accumulation is minimal when administered as a single daily dose. More than 95% of the absorbed dose is excreted as an unchanged compound in the urine.

anlodipine/ valsartan/ hydrochlorothiazide
After oral administration of Exforge HCT® in normal healthy adults, peak plasma concentrations of anlodipine, valsartan and HCT are reached in 6-8 hours, 3 hours and 2 hours respectively. The rate and extent of absorption of anlodipine, valsartan and HCT from Exforge HCT® are the same as when administered as isolated pharmaceutical forms.

Special patient populations
Children
There are no pharmacokinetic data available in pediatric patients with Exforge HCT®.

Geriatric patients
The time taken to reach the peak plasma concentration of anlodipine is similar in young and elderly patients. In elderly patients, anlodipine clearance tends to decrease, leading to an increase in ASC and elimination half-life.

A slightly higher systemic exposure to valsartan was observed in elderly subjects than in young subjects; however, this proved to be of no clinical significance.

Limited data suggest that the systemic clearance of hydrochlorothiazide is reduced in both healthy elderly and hypertensive elderly compared to healthy young volunteers.

Patients with renal impairment
The pharmacokinetics of anlodipine are not significantly influenced by renal impairment.
There is no apparent correlation between renal function (measured by GFR) and valsartan exposure (measured by ASC) in patients with different degrees of renal impairment. Patients with mild or moderate renal impairment can therefore receive the usual starting dose (see "Dosage and instructions for use" and "Warnings and precautions").

In the presence of renal impairment, the mean peak plasma levels and ASC values of hydrochlorothiazide are increased and the urinary excretion rate is reduced. In patients with mild to moderate renal impairment, the elimination half-life is almost doubled. The renal clearance of hydrochlorothiazide is also greatly reduced when compared to the renal clearance of 300 mL/min of patients with normal renal function. Therefore, Exforge HCT® should be used with caution in patients with severe renal impairment (GFR < 30 mL/min) (see "Warnings and precautions").

Patients with hepatic impairment
Patients with hepatic impairment have reduced clearance of anlodipine, with a consequent increase in ASC of approximately 40-60%. On average, exposure to valsartan is twice as high in patients with mild to moderate chronic liver disease (measured by ASC values) than in healthy volunteers (combined by age, gender and weight). Liver disorders do not significantly affect the pharmacokinetics of hydrochlorothiazide, so no dose reduction is necessary. However, Exforge HCT® should be used with special caution in patients with obstructive biliary disorders and severe hepatic impairment (see "Warnings and precautions").

Pre-clinical safety data
anlodipine/ valsartan/ hydrochlorothiazide
In several pre-clinical safety studies carried out on various animal species with anlodipine/valsartan/hydrochlorothiazide (Exforge HCT®), no relevant findings were found that could exclude the use of therapeutic doses of Exforge HCT® in humans.

Pre-clinical safety studies were conducted with anlodipine/valsartan/hydrochlorothiazide in rats, lasting up to 13 weeks, and the no-observed-adverse-effect dose (NOAEL) was determined to be 0.5/8/1.25 mg/Kg/day. Higher doses of this combination (≥ 2/32/5 mg/Kg/day) resulted in an expected reduction in the mass of red blood cells (erythrocytes,

hemoglobin, hematocrit and reticulocytes), increased plasma urea, creatinine and potassium, juxtaglomerular hyperplasia in the kidney and focal erosions in the glandular stomach in rats. All these changes were reversible after a 4-week recovery period, and the pharmacological effects were considered exaggerated.

The combination of anlodipine/valsartan/hydrochlorothiazide has not been tested for mutagenicity, carcinogenicity, clastogenicity and reproductive performance and there is no evidence of interaction between these three drugs, which have been on the market for some time.

anlodipine
The safety data for anlodipine are well established both clinically and non-clinically. No relevant findings were observed in carcinogenicity and mutagenicity studies.
There were no effects on fertility in rats treated with anlodipine (males for 64 days and females 14 days before mating) at doses up to 10 mg/kg/day (8 times the maximum recommended human dose of 10 mg on a mg/m2 basis, based on patients weighing 50 kg).
Anlodipine has been individually tested for mutagenicity, clastogenicity, reproductive performance and carcinogenicity with negative results.

valsartan
Preclinical data revealed no special risks for humans, based on conventional studies of pharmacological safety, genotoxicity, carcinogenic potential and effects on fertility.

Pharmacological safety and long-term toxicity: In a variety of preclinical safety studies conducted in different animal species, there were no findings that could exclude the use of therapeutic doses of valsartan in humans. In the preclinical safety studies, high doses of valsartan (200 to 600 mg/kg/day of body weight) caused a reduction in red blood cell parameters (erythrocytes, hemoglobin and hematocrit) in rats and evidence of changes in renal hemodynamics (slightly increased blood urea nitrogen and renal tubular hyperplasia and basophilia in males). These doses in rats (200 and 600 mg/kg/day) are approximately 6 and 18 times the maximum recommended dose for humans on a mg/m^2 basis (calculations assume an oral dose of 320 mg/day and a 60 kg patient). At comparable doses, in marmoset monkeys, the changes were similar, although more severe particularly in the kidneys, where the changes evolved into nephropathy which included an increase in blood urea nitrogen and creatinine. Hypertrophy of the renal juxtaglomerular cells was also observed in both species. All the alterations were considered to be caused by the pharmacological action of valsartan, which produces prolonged hypotension, particularly in marmoset monkeys. For therapeutic doses of valsartan in humans, hypertrophy of renal juxtaglomerular cells does not appear to be of any relevance.

Reproductive toxicity: In a fertility study in rats, valsartan showed no adverse reactions on the reproductive performance of male or female rats at oral doses of up to 200 mg/kg/day. Approximately 6 times the maximum recommended human dose in mg/m^2 (calculations assume an oral dose of 320 mg/day in a 60kg patient).

Mutagenicity: Valsartan was free of mutagenic potential in genotoxicity studies, either at the gene or chromosome level, when investigated in multiple in vitro and in vivo patterns.

Carcinogenicity: There was no evidence of carcinogenicity when valsartan was administered in the diet to mice and rats for 2 years at doses of up to 160 and 200 mg/kg/day, respectively.

hydrochlorothiazide
Hydrochlorothiazide was individually tested for mutagenicity, clastogenicity, reproductive performance and carcinogenicity, with negative results. According to the available experimental data, hydrochlorothiazide showed no evidence of carcinogenic activity in rats and mice (hepatocellular tumors were observed only in highly dosed male mice; the incidence did not exceed the levels historically found in controls). The mutagenic potential was evaluated in a series of in vitro and in vivo test systems. Although some positive results were obtained in vitro, all in vivo studies provided negative results. Hydrochlorothiazide improved UVA-induced pyrimidine dimer formation in vitro and in mouse skin after oral treatment. It is therefore concluded that there is no relevant mutagenic potential in vivo, although hydrochlorothiazide may potentiate the genotoxic effects of UVA light.

valsartan and hydrochlorothiazide
In several pre-clinical safety studies carried out on various species of animals, there were no findings to exclude the use of therapeutic doses of valsartan and hydrochlorothiazide in humans. High doses of valsartan:hydrochlorothiazide (100:31.25 to 600: 187.5 mg/kg body weight) caused a reduction in red blood cell parameters (erythrocytes, hemoglobin and hematocrit) in rats and showed evidence of alterations in renal hemodynamics (moderate to severe increase in plasma urea, increase in plasma potassium and magnesium, slight increase in urinary volume of electrolytes, minimal to slight tubular basophilia and hypertrophy of the afferent arteriole with the highest dose). In marmoset monkeys (doses of 30:9,375 to 400:125 mg/kg), the changes were similar, but more pronounced, particularly with the highest dose, and especially in the kidneys, where the changes evolved into a nephropathy with elevated urea and creatinine. Marmosets also had alterations in the gastrointestinal mucosa at 30:9,373 to 400:125 mg/kg.

Hypertrophy of the renal juxtaglomerular cells was also observed in rats and marmosets. It was considered that all the alterations were caused by the pharmacological action of valsartan: hydrochlorothiazide which is synergistic (potentiation of the effect about 10 times when compared to that of valsartan alone) rather than additive, producing prolonged hypotension, particularly in marmoset monkeys. For therapeutic doses of valsartan: hydrochlorothiazide in humans, hypertrophy of renal juxtaglomerular cells does not appear to be of any relevance. The main pre-clinical safety findings are attributed to the pharmacological action of the compounds, which appear to act synergistically, without any evidence of interaction between the two compounds. In the clinic, the action of the two compounds is additive and the pre-clinical findings do not prove to have any clinical significance.

The combination valsartan: hydrochlorothiazide has not been tested for mutagenicity, clastogenicity, reproductive performance and carcinogenicity, as there is no evidence for any interaction between the two compounds.

valsartan and anlodipine
In a variety of pre-clinical safety studies conducted in many animal species with valsartan/anlodipine, no findings were found that could exclude the use of therapeutic doses of valsartan/anlodipine in humans. Animal studies lasting 13 weeks were conducted with this combination in rats and marmosets, as well as studies in mice to investigate the development of embryonic and fetal toxicity.

In a 13-week oral toxicity study in rats, valsartan/anlodipine-related glandular stomach inflammation was observed in males at doses ≥ 48/3 mg/kg/day and in females at doses ≥ 7.5/120 mg/kg/day. No effect was observed in the 13-week study in marmoset monkeys at any dose, although inflammation of the large intestine was observed at high doses in marmoset monkeys only (no effect at doses < 80/5 mg/kg/day). The gastrointestinal adverse reactions observed in clinical studies with Exforge HCT® were no more frequent with the combination than with the respective monotherapies.

The combination of valsartan/anlodipine was not tested for mutagenicity, clastogenicity, carcinogenicity and reproductive performance as there was no evidence of any interaction between the two compounds.

4. CONTRAINDICATIONS
Known hypersensitivity to valsartan, hydrochlorothiazide, anlodipine, dihydropyridines, other sulfonamide derivatives or to any of the excipients.

Pregnancy (see "Pregnancy, lactation, women and men of childbearing age").
This medication belongs to risk category D in pregnancy, so **this medication should not be used by pregnant women without medical advice. Inform your doctor immediately if you suspect you may be pregnant. Contraindicated for use when breastfeeding or donating human milk.**

Due to hydrochlorothiazide, Exforge HCT® is contraindicated in patients with anuria.
Concomitant use of angiotensin receptor blockers (ARBs) - including valsartan - or angiotensin-converting enzyme inhibitors (ACEIs) with aliskiren in patients with type 2 diabetes (see "Drug-drug interactions").

NOVARTIS

5. WARNINGS AND PRECAUTIONS

Patients with sodium depletion and/or hypovolemia

In a controlled study in patients with moderate to severe uncomplicated hypertension, excessive hypotension was observed, including orthostatic hypotension in 1.7% of patients treated with the maximum dose of Exforge HCT® (320/25/10) compared to 1.8% of patients with valsartan/HCT (320/25), 0.4% of patients with anlodipine/valsartan (10/320) and 0.2% of patients with HCT/anlodipine (25/10 mg). In patients with severe sodium depletion and/or hypovolemia, such as those receiving high doses of diuretics, symptomatic hypotension may occur in rare cases after starting therapy with Exforge HCT®. Exforge HCT® should only be used after correcting any pre-existing sodium depletion and/or hypovolemia, otherwise treatment should be started under medical supervision.

If excessive hypotension occurs with the use of Exforge HCT®, keep the patient in the supine position and, if necessary, administer intravenous infusion of physiological saline solution. Treatment can be restarted once blood pressure has stabilized.

Patients with renal impairment

Due to the hydrochlorothiazide component, Exforge HCT® should be used with caution in patients with severe renal impairment (GFR < 30 mL/min). Thiazide diuretics can precipitate azotemia in patients with chronic kidney disease. They are ineffective as monotherapy in severe renal impairment (GFR < 30 mL/min), but may be useful when used with caution in combination with loop diuretics even in patients with GFR < 30 mL/min (see "Warnings and precautions" and "Pharmacological characteristics"). No dose adjustment of Exforge HCT® is necessary in patients with mild to moderate renal impairment.

The use of ARBs - including valsartan - or ACE inhibitors together with aliskiren should be avoided in patients with severe renal impairment (GFR < 30 mL/min) (see "Drug-drug interactions").

Patients with renal artery stenosis

Exforge HCT® should be used with caution to treat hypertension in patients with unilateral or bilateral renal artery stenosis or single kidney stenosis, as blood urea and serum creatinine concentrations may increase in these patients.

Kidney transplant patients

There is no experience of using Exforge HCT® in patients with a recent kidney transplant.

Patients with hepatic impairment

Valsartan is mainly eliminated via bile in unchanged form, while anlodipine is largely metabolized by the liver. Due to the components valsartan, hydrochlorothiazide and anlodipine, special caution should be exercised when administering Exforge HCT® to patients with hepatic impairment or obstructive biliary disorders (see "Dosage" and "Pharmacological characteristics").

Angioedema

Angioedema has been reported in patients treated with valsartan, including swelling of the larynx and glottis, causing airway obstruction and/or swelling of the face, lips, pharynx and/or tongue. Some of these patients had previously experienced angioedema with other drugs, including ACE inhibitors. Exforge HCT® should be immediately discontinued in patients who develop angioedema and should not be re-administered.

Patients with heart failure/post-myocardial infarction

In general, calcium channel blockers, including anlodipine, should be used with caution in patients with severe congestive heart failure (New York Heart Association (NYHA) functional class III-IV).

In patients in whom renal function may depend on the activity of the angiotensin-renin-aldosterone system (e.g. patients with severe congestive heart failure), treatment with angiotensin-converting enzyme inhibitors or angiotensin receptor blockers has been associated with oliguria and/or progressive azotemia, and in rare cases, with acute renal impairment and/or death. The evaluation of patients with heart failure or post-myocardial infarction should always include an assessment of renal function.

Patients with acute myocardial infarction

Worsening of angina pectoris and acute myocardial infarction may develop after starting or increasing the dose of anlodipine, particularly in patients with severe obstructive coronary artery disease.

Patients with aortic and mitral valve stenosis, hypertrophic obstructive cardiomyopathy
As with other vasodilators, special caution is required when anlodipine is used in patients who have aortic or mitral stenosis or hypertrophic obstructive cardiomyopathy.

Heart failure
anlodipine
In a long-term, placebo-controlled study of anlodipine (PRAISE-2) in patients with NYHA III and IV heart failure of non-ischemic etiology, anlodipine was associated with an increase in pulmonary edema events despite there being no significant difference in the incidence of worsening heart failure when compared to placebo.

Serum electrolyte changes
Concomitant use with potassium supplements, potassium-sparing diuretics, potassium-containing salt substitutes or other medications that increase plasma potassium concentration (heparin, etc.) can lead to hyperkalemia and should be used with caution. Thiazide diuretics can precipitate a new onset of hypokalemia or exacerbate pre-existing hypokalemia. Thiazide diuretics should be administered with caution in patients with conditions involving high potassium loss, for example salt-depleting nephropathy and pre-renal (cardiogenic) impairment of renal function. If hypokalemia is accompanied by clinical signs (e.g. muscle weakness, paresis or ECG changes), Exforge HCT® should be discontinued. Correction of hypokalemia and any coexisting hypomagnesemia is recommended before starting thiazides. Serum potassium and magnesium concentrations should be periodically checked. All patients receiving thiazide diuretics should be monitored for electrolyte imbalances, particularly potassium.
Thiazide diuretics can precipitate a new onset of hyponatremia and hypochloremic alkalosis or exacerbate pre-existing hyponatremia. Hyponatremia accompanied by neurological symptoms (nausea, progressive disorientation, apathy) has been observed in isolated cases. Regular monitoring of serum sodium concentrations is recommended.

anlodipine - valsartan - hydrochlorothiazide
In the controlled clinical study of Exforge HCT® in patients with moderate to severe hypertension, the incidence of hypokalemia (serum potassium < 3.5 mEq/L) with the maximum dose of Exforge HCT® (320/25/10 mg) was 9.9% at any time after start, compared to 24.5% with HCT/anlodipine (25/10 mg), 6.6% with valsartan/HCT (320/25 mg) and 2.7% with anlodipine/valsartan (10/320 mg). One patient (0.2%) discontinued therapy due to a hypokalemia event in each of the Exforge HCT® and HCT/anlodipine groups. The incidence of hyperkalemia (plasma potassium > 5.7 mEq/L) was 0.4% with Exforge HCT® compared to 0.2-0.7% with dual therapies.
In the controlled clinical study with Exforge HCT®, the opposing effects of valsartan 320 mg and hydrochlorothiazide 25 mg on serum potassium were approximately balanced in many patients. In others, one or the other effect may be dominant. Periodic determinations of serum electrolytes to detect electrolyte imbalances should be carried out at appropriate time intervals.

Systemic lupus erythematosus
It has been reported that thiazide diuretics, including hydrochlorothiazide, can exacerbate or activate systemic lupus erythematosus.

Other metabolic disorders
Thiazide diuretics, including hydrochlorothiazide, can alter glucose tolerance and can raise plasma levels of cholesterol and triglycerides.
Like other diuretics, hydrochlorothiazide can raise serum uric acid concentrations due to reduced uric acid clearance and can cause or exacerbate hyperuricemia and precipitate gout in susceptible patients.
Thiazide diuretics decrease urinary calcium excretion and may cause a slight increase in serum calcium in the absence of known calcium metabolism disorders. Since hydrochlorothiazide can raise serum calcium concentrations, it should be used with caution in patients with hypercalcemia. Hypercalcemia unresponsive to thiazide withdrawal or ≥ 12 mg/dL may be evidence of an underlying hypercalcemic process independent of thiazides. Pathological changes in the parathyroid gland of patients with hypercalcemia and hypophosphatemia have been observed in some patients on long-term thiazide therapy. If hypercalcemia occurs, clarification of the diagnosis is required.

General
Hypersensitivity reactions to hydrochlorothiazide are more likely in patients with allergies and asthma.

Acute angle-closure glaucoma
Hydrochlorothiazide, a sulfonamide, has been associated with an idiosyncratic reaction resulting in transient acute myopia and acute angle-closure glaucoma. Symptoms include acute onset of reduced visual acuity or eye pain and typically occur within hours to weeks of starting therapy. If left untreated, acute angle-closure glaucoma can lead to permanent vision loss.
The primary treatment is to discontinue hydrochlorothiazide as soon as possible. Immediate medical or surgical treatment may need to be considered if intraocular pressure remains uncontrolled. Risk factors for developing acute angle-closure glaucoma may include a history of allergy to sulfonamide or penicillin.

Dual Blockade of the Renin-Angiotensin System (RAS)
Caution is required when co-administering ARBs, including valsartan, with other agents that block the RAS such as ACEIs or aliskiren (see "Drug-drug interactions").

Non-melanoma skin cancer
An increased risk of non-melanoma skin cancer (NMSC) [basal cell carcinoma (BCC) and squamous cell carcinoma (SCC)] with increasing cumulative dose exposure to hydrochlorothiazide in two epidemiological studies based on the Danish National Cancer Registry. The risk of NMSC appears to increase with long-term use (see "Pharmacological Characteristics"). Photosensitizing actions of hydrochlorothiazide may act as a possible mechanism for NMSC.
Patients taking hydrochlorothiazide should be informed of the risk of NMSC and advised to regularly check their skin for new lesions and to immediately report any suspicious skin lesions. Possible preventive measures, such as limited exposure to sunlight and adequate protection when exposed to sunlight, should be advised to patients in order to minimize the risk of skin cancer. Suspicious skin lesions should be promptly examined, potentially including histological examination of biopsies. The use of hydrochlorothiazide may also have to be reconsidered in patients who have already had NMSC (see "Adverse Reactions").

Elderly patients
No initial dose adjustment is required for elderly patients (see "Pharmacological Characteristics").

Children and adolescents
Exforge HCT® is not recommended for patients under the age of 18, due to the lack of safety and efficacy data.

Pregnancy, lactation, women and men of childbearing age
Pregnancy
Risk summary
As with any drug that acts directly on the RAAS, Exforge HCT® should not be used during pregnancy (see "Contraindications"). Due to the mechanism of action of angiotensin II antagonists, the risk to the fetus cannot be excluded. It has been reported that the administration of angiotensin-converting enzyme (ACE) inhibitors, a specific class of medications that act on the renin-angiotensin-aldosterone system (RAAS), to pregnant women during the second and third trimesters of pregnancy causes injury and death to developing fetuses. In addition, in retrospective data, the use of ACE inhibitors in the first trimester was associated with a potential risk of birth defects. Hydrochlorothiazide crosses the placenta. There have been reports of spontaneous abortion, oligohydramnios and renal dysfunction in the newborn when the pregnant woman inadvertently took valsartan. There are no adequate clinical data on amlodipine in pregnant women. Animal studies with amlodipine have shown reproductive toxicity at doses of up to eight times the maximum recommended dose of 10 mg in humans (see "Animal data"). The potential risk in humans is unknown. Intrauterine exposure to thiazide diuretics, including hydrochlorothiazide, is associated with fetal or neonatal jaundice or thrombocytopenia, and may be associated with other adverse reactions that occur in adults. If pregnancy occurs during treatment, Exforge HCT® should be discontinued as soon as possible (see "Animal data").

Clinical considerations
Maternal and/or embryonic/fetal risk associated with the disease
Hypertension in pregnancy increases the maternal risk of pre-eclampsia, gestational diabetes, premature birth and birth complications (e.g. need for caesarean section and post-partum hemorrhage). Hypertension increases the fetal risk of intrauterine growth restriction and intrauterine death.

Fetal/neonatal risk

Oligohydramnios in pregnant women using drugs that affect the renin-angiotensin system in the second and third trimesters of pregnancy can result in: reduced fetal renal function leading to anuria and renal impairment, fetal lung hypoplasia, skeletal deformities, including cranial hypoplasia, hypotension and death.
In the event of accidental exposure to ARB therapy, appropriate fetal monitoring should be considered.
Infants whose mothers have received ARB therapy should be carefully observed for hypotension.

Animal data

Valsartan

In studies of embryonic development in mice, rats and rabbits, fetotoxicity in association with maternal toxicity was observed in rats at doses of valsartan of 600 mg / kg / day approximately 18 times the maximum recommended human dose on a mg / m^2 basis (calculations assume an oral dose of 320 mg / day in a 60 kg patient) and in rabbits at doses of 10 mg / kg / day approximately 0, 6 times the maximum recommended human dose on a mg / m^2 basis (calculations assume an oral dose of 320 mg / day in a 60 kg patient). There was no evidence of maternal toxicity or fetotoxicity in mice up to a dose level of 600 mg / kg / day approximately 9 times the maximum recommended human dose in mg / m^2 (calculations assume an oral dose of 320 mg / day in a 60 kg patient)

Hydrochlorothiazide

Hydrochlorothiazide was not teratogenic and had no effects on fertility and conception. No teratogenic potential was revealed in 3 animal species tested. There was no dose-related fetotoxicity at dose levels 0, 100, 300 and 1000 mg/kg in rats. A decrease in weight gain in lactating rat pups was attributed to the high dose and diuretic effects of hydrochlorothiazide, with subsequent effects on milk production.

Anlodipine

No evidence of teratogenicity or embryofetal toxicity was found when pregnant rats and rabbits were treated orally with anlodipine maleate at doses of up to 10 mg/kg/day during the respective periods of organogenesis. However, litter size was significantly reduced (to approximately 50%) and the number of intrauterine deaths was significantly increased (approximately 5-fold). Anlodipine has been shown to prolong both the gestation period and the duration of parturition in rats at this dose.

Valsartan and anlodipine

In an oral embryofetal development study in rats with dose levels of 80/5 mg/kg/day valsartan/anlodipine, 160/10 mg/kg/day valsartan/anlodipine and 320/20 mg/kg/day valsartan/anlodipine, treatment-related maternal and fetal effects (developmental delay and changes noted in the presence of significant maternal toxicity) were noted with the high doses combined. The no-observed-adverse-effect level (NEANO/NOAEL) for embryofetal effects was 160/10 mg/kg/day of valsartan/anlodipine. These doses are, respectively, 4.3 and 2.7 times the systemic exposure in humans receiving the maximum recommended human dose (MRHD) (320/10 mg/60 kg).

Lactation

It is not known whether valsartan is transferred into human milk. It has been reported that anlodipine is transferred into human milk. The proportion of the maternal dose received by the baby has been estimated with an interquartile range of 3 to 7%, with a maximum of 15%. The effect of anlodipine in children is unknown. Valsartan has been transferred to the milk of lactating rats. Hydrochlorothiazide is transferred to human milk. Therefore, the use of Exforge HCT® in breastfeeding women is not recommended.

Women and men of childbearing age

As with any drug that acts directly on RAAS, Exforge HCT® should not be used by women who are planning to become pregnant. Doctors prescribing any agent that acts on the RAAS should advise women of childbearing potential about the potential risk of these agents during pregnancy.

Infertility
There are no data on the effects of anlodipine, valsartan or hydrochlorothiazide on human fertility. Studies in rats have not shown any effect of anlodipine, valsartan or hydrochlorothiazide on fertility (see "Pre-clinical safety data").

Effects on ability to drive vehicles or operate machinery
No studies have been carried out on the effects on the ability to drive vehicles or operate machinery. When driving vehicles or operating machinery, it should be borne in mind that dizziness or fatigue may occasionally occur.

Inform your patient that they should avoid getting up quickly, driving vehicles and/or operating machinery, especially during the entire course of treatment.

Warning: Contains the dyes titanium dioxide (only Exforge HCT® 160/12.5/5 mg, 160/12.5/10 mg and 160/25/5 mg), iron oxide yellow (except Exforge HCT® 160/12.5/5 mg) and iron oxide red (only Exforge HCT® 160/12.5/10 mg) which may cause allergic reactions.

This medication may cause doping.

6. DRUG-DRUG INTERACTIONS
- valsartan - hydrochlorothiazide
The following drug-drug interactions may occur due to the two components (valsartan and/or hydrochlorothiazide) of Exforge HCT®:

Lithium: reversible increases in serum lithium concentrations and toxicity have been reported during concomitant administration of lithium and ACE inhibitors, angiotensin II receptor antagonists or thiazides. Since the renal clearance of lithium is reduced by thiazides, the risk of lithium toxicity can presumably be further increased with Exforge HCT®. Therefore, careful monitoring of serum lithium concentrations is recommended during concomitant use.

- anlodipine
The following potential drug-drug interactions may occur due to the anlodipine component of Exforge HCT®:

Simvastatin: coadministration of multiple doses of 10 mg anlodipine with 80 mg simvastatin resulted in a 77% increase in simvastatin exposure compared to simvastatin alone. A dose limit of 20 mg of simvastatin per day is recommended for patients taking anlodipine.

CYP3A4 inhibitors: coadministration of a 180 mg dose of diltiazem with 5 mg of anlodipine in elderly hypertensive patients resulted in a 1.6-fold increase in the systemic exposure of anlodipine. However, potent CYP3A4 inhibitors (e.g. ketoconazole, itraconazole and ritonavir) can increase plasma concentrations of anlodipine to a greater extent than diltiazem. Caution should be exercised when anlodipine is co-administered with CYP3A4 inhibitors.

Grapefruit juice: anlodipine exposure may be increased when co-administered with grapefruit juice due to CYP3A4 inhibition. However, coadministration of 240 mL of grapefruit juice with a single oral dose of 10 mg of anlodipine in 20 healthy volunteers showed no significant effect on the pharmacokinetics of anlodipine.

CYP3A4 inducers: there is no information available on the quantitative effects of CYP3A4 inducers on anlodipine. Patients should be adequately monitored for clinical effects when anlodipine is co-administered with CYP3A4 inducers (e.g. rifampicin, hypericum perforatum).

In monotherapy, anlodipine is safely administered with thiazide diuretics, beta-blockers, angiotensin-converting enzyme inhibitors, long-acting nitrates, sublingual nitroglycerins, digoxin, warfarin, atorvastatin, sildenafil, Maalox® (aluminum hydroxide gel, magnesium hydroxide and simethicone), cimetidine, non-steroidal anti-inflammatory drugs, antibiotics and oral hypoglycemic drugs.

- valsartan
The following potential drug-drug interactions may occur due to the valsartan component of Exforge HCT®:

Dual blockade of the Renin-Angiotensin System (RAS) with ARBs, ACEIs or aliskiren: concomitant use of ARBs, including valsartan, with other medications that act on the RAS is associated with an increased incidence of hypotension, hyperkalemia and changes in renal function compared to monotherapy. It is recommended to monitor blood pressure, kidney function and electrolytes in patients being treated with Exforge HCT® and other RAS inhibitors (see "Warnings and precautions").

The concomitant use of ARBs, including valsartan, or ACEIs with aliskiren should be avoided in patients with severe renal impairment (GFR < 30 mL/min) (see "Warnings and precautions").
The concomitant use of ARBs, including valsartan, or ACEIs with aliskiren is contraindicated in patients with type 2 diabetes (see "Contraindications").

Potassium: concomitant use with potassium supplements, potassium-sparing diuretics, potassium-containing salt substitutes or other medications or substances that may increase potassium levels (heparin, etc.) requires caution and frequent monitoring of potassium levels.

Non-steroidal anti-inflammatory drugs (NSAIDs) including selective cyclooxygenase-2 inhibitors (COX-2 inhibitors): when angiotensin II antagonists are administered simultaneously with NSAIDs, attenuation of the antihypertensive effects may occur. Furthermore, in elderly patients with hypovolemia (including those on diuretic therapy) or who have impaired renal function, the concomitant use of angiotensin II antagonists and NSAIDs may lead to an increased risk of worsening renal function. Therefore, monitoring of renal function is recommended when starting or changing the treatment of patients taking valsartan who are taking NSAIDs concomitantly.

Transporters: the results of an in vitro study with human liver tissue indicated that valsartan is a substrate of the hepatic uptake transporter OATP1B1 and the hepatic efflux transporter MRP2. Coadministration of inhibitors of the uptake transporter (rifampicin and ciclosporin) or the efflux transporter (ritonavir) can increase systemic exposure to valsartan.

In monotherapy with valsartan, no clinically significant drug-drug interactions were found with the following drugs: cimetidine, warfarin, furosemide, digoxin, atenolol, indomethacin, hydrochlorothiazide, anlodipine, glibenclamide.

- hydrochlorothiazide
The following potential drug-drug interactions may occur due to the hydrochlorothiazide component of Exforge HCT®:

Other antihypertensive medications: thiazide diuretics potentiate the action of other antihypertensive medications (e.g. guanethidine, methyldopa, beta-blockers, vasodilators, calcium channel blockers, ACE inhibitors, angiotensin receptor blockers (ARBs) and direct renin inhibitors (DRIs)).

Musculoskeletal relaxants: thiazide diuretics, including hydrochlorothiazide, potentiate the action of musculoskeletal relaxants, such as curare derivatives.

Medications that affect serum potassium concentration: the hypokalemic effect of diuretics can be increased by concomitant administration of potassium-depleting diuretics, corticosteroids, ACTH, amphotericin, carbenoxolone, penicillin G and salicylic acid derivatives or antiarrhythmics (see "Warnings and precautions").

Medications that affect serum sodium concentrations: the hyponatremic effect of diuretics can be intensified by the concomitant administration of medications such as antidepressants, antipsychotics, antiepileptics, etc. Caution is advised in the long-term administration of these drugs (see "Warnings and precautions").

Antidiabetic agents: thiazide diuretics can change glucose tolerance. It may be necessary to adjust the dose of insulin and oral antidiabetics.

Digitalis glycosides: hypokalemia or hypomagnesemia induced by thiazide diuretics may occur as an undesired effect, which favors the incidence of digitalis-induced cardiac arrhythmia (see "Warnings and precautions").

NSAIDs and selective COX-2 inhibitors: concomitant administration of NSAIDs (e.g. salicylic acid derivatives, indomethacin) can weaken the diuretic and antihypertensive activity of the thiazide component of Exforge HCT®. Concomitant hypovolemia can induce acute renal impairment.

Allopurinol: coadministration of thiazide diuretics (including hydrochlorothiazide) may increase the incidence of hypersensitivity reactions to allopurinol.

Amantadine: coadministration of thiazide diuretics (including hydrochlorothiazide) may increase the risk of adverse effects caused by amantadine.

Antineoplastic agents (e.g. cyclophosphamide, methotrexate): coadministration of thiazide diuretics can reduce the renal excretion of cytotoxic agents and increase their myelosuppressive effects.

Anticholinergic agents: the bioavailability of thiazide diuretics may be increased by anticholinergic agents (e.g. atropine, biperiden), apparently due to a decrease in gastrointestinal motility and gastric emptying rate. However, prokinetic medications such as cisapride can reduce the bioavailability of thiazide-type diuretics.

Ion exchange resins: the absorption of thiazide diuretics, including hydrochlorothiazide, is reduced by cholestyramine or colestipol. However, staggering the dose of hydrochlorothiazide and resin would probably minimize the interaction, provided that hydrochlorothiazide was administered at least 4 hours before or 4 to 6 hours after the administration of resins.

Vitamin D: the administration of thiazide diuretics, including hydrochlorothiazide, with vitamin D or calcium salts may potentiate the increase in serum calcium.

Cyclosporine: concomitant treatment with cyclosporine may increase the risk of hyperuricemia and gout complications.

Calcium salts: concomitant administration of thiazide-type diuretics can lead to hypercalcemia due to increased tubular reabsorption of calcium.

Diazoxide: thiazide diuretics can increase the hyperglycemic effect of diazoxide.

Methyldopa: the occurrence of hemolytic anemia in the concomitant use of hydrochlorothiazide and methyldopa has been reported in the literature.

Alcohol, barbiturates or narcotics: concomitant administration of thiazide diuretics with alcohol, barbiturates or narcotics can potentiate orthostatic hypotension.

Pressor amines: hydrochlorothiazide can reduce the response to pressor amines such as noradrenaline. The clinical significance of this effect is uncertain and insufficient to exclude its use.

7. MEDICATION STORAGE PRECAUTIONS
Exforge HCT® tablets should be stored at room temperature (between 15 and 30 °C) and protected from moisture.
The expiry date is 24 months from the date of manufacture.
Batch number and manufacturing and expiry dates: see packaging.
Do not use medicine after the expiration date. Store in its original packaging.

Physical characteristics: Exforge HCT® is available as coated tablets in 5 concentrations.
Exforge HCT® (160/12.5/5 mg): white, oval, biconvex tablet.
Exforge HCT® (160/12.5/10 mg): light yellow, oval, biconvex tablet.

Exforge HCT® (160/25/5 mg): yellow, oval, biconvex tablet.
Exforge HCT® (160/25/10 mg): brownish-yellow, oval, biconvex tablet.
Exforge HCT® (320/25/10 mg): brownish-yellow, oval, biconvex tablet.

Before use, check the appearance of the medication.
All medication should be kept out of the reach of children.

8. DOSAGE AND INSTRUCTIONS FOR USE
Exforge HCT® tablets can be taken with or without food. It is recommended to take Exforge HCT® tablets with a little water. The tablets should be swallowed whole, without chewing.
Administer orally.

Dosage
The recommended dose is 1 tablet a day.
For initial therapy, the starting dose generally used is one tablet of Exforge HCT® 160/12.5/5 mg once a day. The dose can be increased after 1 to 2 weeks from the start of therapy, up to a maximum of one tablet of 320/25/10 mg once a day for blood pressure control, as required.

A patient whose blood pressure is not adequately controlled by dual therapy can be directly switched to combination therapy with Exforge HCT®.

For convenience, patients taking valsartan, anlodipine and HCT in separate tablets can have their treatment switched to Exforge HCT® containing the same doses of the components. A patient who experiences dose-related adverse reactions to any dual combination of Exforge HCT® components can be switched to Exforge HCT® containing a lower dose of that component to achieve similar blood pressure reductions. The dose can be increased after two weeks. The maximum antihypertensive effect achieved by Exforge HCT® is reached within two weeks of changing the dose. The maximum recommended dose of Exforge HCT® is 320/25/10 mg (valsartan/hydrochlorothiazide/anlodipine).

It is advisable to take the medication at the same time every day, preferably in the morning. If you forget to take Exforge HCT®, you should take it as soon as you remember and then take the next dose at the usual time. However, if the next dose is due, the patient should not take the missed dose. The patient should not take a double dose to make up for a missed dose.

Special population
- Geriatric patients (65 years and over)
No initial dose adjustment is required for elderly patients aged 65 and over. Starting with the lowest available dose of anlodipine should be considered. The lowest concentration of Exforge HCT® contains 5 mg of anlodipine (see "Pharmacological characteristics").

- Pediatric patients (under 18 years of age)
Due to the lack of safety and efficacy data, Exforge HCT® is not recommended for patients under the age of 18.

- Patients with renal impairment
Due to the hydrochlorothiazide component, Exforge HCT® is contraindicated in patients with anuria (see "Contraindications") and should be used with caution in patients with severe renal impairment (GFR < 30 mL/min) (see "Warnings and precautions" and "Pharmacological characteristics"). Thiazide diuretics are ineffective as monotherapy in severe renal impairment (GFR < 30 mL/min), but can be useful in these patients when used with due caution and in combination with a loop diuretic, even in patients with GFR < 30 mL/min. No dose adjustment of Exforge HCT® is necessary in patients with mild to moderate renal impairment.

- Patients with hepatic impairment
Due to the components valsartan, hydrochlorothiazide and anlodipine, Exforge HCT® should be used with special caution in patients with hepatic impairment or obstructive biliary disorders. Starting with the lowest available dose of anlodipine should be considered. The lowest concentration of Exforge HCT® contains 5 mg of anlodipine (see "Warnings and precautions" and "Pharmacological characteristics").

This medication should not be broken or chewed.

9. ADVERSE REACTIONS

The presentation of the safety profile of Exforge HCT® is based on experience with Exforge HCT® and the components alone.

Exforge HCT® information

The safety of Exforge HCT® was assessed at its maximum dose of 320/25/10 mg in a controlled clinical study of 2,271 patients, 582 of whom received valsartan in combination with anlodipine and HCT. No new reactions were found, especially with Exforge HCT®, other than those already known to be associated with individual monotherapy. No additional risks to those previously identified with long-term treatment were observed. In general, Exforge HCT® is well tolerated regardless of gender, age or race. The changes in laboratory parameters observed with the Exforge HCT® combination were minor and consistent with the pharmacological mechanism of action of the agents in monotherapy. The hypokalemic effect of HCT is attenuated by the presence of valsartan in both the triple combination and the double combination with HCT.

Additional information on the individual components

Adverse reactions previously reported with the individual components may occur with Exforge HCT®, even if they were not observed in pivotal clinical studies.

anlodipine

As the clinical studies of anlodipine were conducted under widely varying conditions, the rates of adverse experiences observed in the clinical studies of one medication cannot be directly compared with the rates in the clinical studies of other medications and may not reflect the rate observed in practice.

Adverse reactions reported with anlodipine monotherapy, disregarding the causal association with the medication studied, were as follows:

Table 1 - Adverse experiences with anlodipine monotherapy

**Blood and lymphatic system disorders**	
Very rare	Thrombocytopenia, leukopenia
**Immune system disorders**	
Very rare	Allergic reactions
**Metabolic and nutritional disorders**	
Very rare	Hyperglycemia
**Psychiatric disorders**	
Uncommon	Insomnia, mood swings, including anxiety
**Nervous system disorders**	
Common	Headache, drowsiness, dizziness
Uncommon	Tremor, hypoesthesia, dysgeusia, paresthesia, syncope
Very rare	Peripheral neuropathy, hypertonia
**Eye disorders**	
Uncommon	Visual impairment, diplopia
**Hearing and labyrinth disorders**	
Uncommon	Tinnitus
**Cardiac disorders**	
Common	Palpitations
Very rare	Arrhythmia, bradycardia, atrial fibrillation, ventricular tachycardia, myocardial infarction
**Vascular disorders**	
Common	Flushing
Uncommon	Hypotension
Very rare	Vasculitis
**Respiratory, thoracic and mediastinal disorders**	
Uncommon	Dyspnea, rhinitis
Very rare	Cough

**Gastrointestinal disorders**	
Common	Abdominal pain, nausea
Uncommon	Vomiting, dyspepsia, dry mouth, constipation, diarrhea
Very rare	Pancreatitis, gastritis, gingival hyperplasia
**Hepatobiliary disorders**	
Very rare	Hepatitis, jaundice
**Skin and subcutaneous tissue disorders**	
Uncommon	Alopecia, hyperhidrosis, pruritus, rash, purpura, skin discoloration, photosensitivity
Very rare	Angioedema, urticaria, erythema multiforme, Stevens-Johnson syndrome
**Connective tissue and musculoskeletal disorders**	
Uncommon	Back pain, muscle spasms, myalgia, arthralgia
**Urinary and renal disorders**	
Uncommon	Urination disorders, nocturia, pollakiuria
**Reproductive system and breast disorders**	
Uncommon	Gynecomastia, erectile dysfunction
**General and application site disorders**	
Common	Edema, fatigue
Uncommon	Asthenia, pain, malaise, chest pain
**Laboratory**	
Uncommon	Weight loss, weight gain
Very rare	Increased liver enzymes (most commonly cholestasis)

valsartan

The adverse reactions reported in the hypertension indication from clinical studies, post-marketing experience and laboratory findings are listed below according to organ system classification. All adverse reactions reported in post-marketing experience and laboratory findings have a frequency described as "unknown" since it is not possible to determine them.

Table 2 - Adverse Reactions with valsartan

**Disorders of the lymphatic and blood systems**	
Unknown	Decreased hemoglobin, decreased hematocrit, neutropenia, thrombocytopenia
**Immune system disorders**	
Unknown	Hypersensitivity including serum sickness
**Nutritional and metabolic disorders**	
Unknown	Increased serum potassium
**Labyrinth and ear disorders**	
Uncommon	Vertigo
**Vascular disorders**	
Unknown	Vasculitis
**Mediastinal, thoracic and respiratory disorders**	
Uncommon	Cough
**Gastrointestinal disorders**	
Uncommon	Abdominal pain
**Hepatobiliary disorders**	
Unknown	Abnormal liver function tests including increased bilirubin in the blood
**Subcutaneous tissue and skin disorders**	
Unknown	Angioedema, bullous dermatitis, rash and itching

**Connective tissue and musculoskeletal disorders**	
Unknown	Myalgia
**Urinary and renal disorders**	
Unknown	Renal impairment and dysfunction, increased creatinine in the blood
**General disorders and administration site conditions**	
Uncommon	Fatigue

The following events were also observed during clinical studies with hypertensive patients, disregarding their causal association with the study medication: insomnia, decreased libido, pharyngitis, rhinitis, sinusitis, upper respiratory tract infection, viral infections.

hydrochlorothiazide

Hydrochlorothiazide has been extensively prescribed over the years, often at doses higher than that contained in Exforge HCT®. The following adverse reactions have been reported in patients treated with thiazide diuretics, in monotherapy, including hydrochlorothiazide.

Table 3 Adverse reactions with hydrochlorothiazide

**Benign, malignant and non-specific neoplasms (including cysts and polyps)**	
Unknown	Non-melanoma skin cancer (basal cell carcinoma and squamous cell carcinoma) (See "Warnings and Precautions and Pharmacological Characteristics")
**Blood and lymphatic system disorders**	
Rare	Thrombocytopenia, sometimes with purpura
Very rare	Leukopenia, agranulocytosis, bone marrow failure and hemolytic anemia
Unknown	Aplastic anemia
**Immune system disorders**	
Very rare	Necrotizing vasculitis, hypersensitivity reactions - respiratory distress, including pneumonitis and pulmonary edema
**Metabolism and nutrition disorders**	
Very common	Hypokalemia (especially at high doses), increase in blood lipids
Common	Hyponatremia, hypomagnesemia, hyperuricemia and decreased appetite
Rare	Hypercalcemia, hyperglycemia, glycosuria and worsening of diabetic metabolic state
Very rare	Hypochloremic alkalosis
**Psychiatric disorders**	
Rare	Sleep disorders
**Nervous system disorders**	
Rare	Headache, dizziness, depression and paresthesia
**Eye disorders**	
Rare<br>Unknown	Visual impairment, particularly in the first few weeks of treatment<br>Closed-angle glaucoma
**Cardiac disorders**	
Rare	Arrhythmias
**Vascular disorders**	
Common	Orthostatic hypotension, which can be aggravated by alcohol, anesthetics or sedatives
**Gastrointestinal disorders**	
Common	Mild nausea and vomiting
Rare	Abdominal discomfort, constipation and diarrhea

Very rare	Pancreatitis
**Hepatobiliary disorders**	
Rare	Cholestasis or jaundice
**Skin and subcutaneous tissue disorders**	
Common	Hives and other forms of rash
Rare	Photosensitivity reaction
Very rare	Toxic epidermal necrolysis, reactions resembling cutaneous lupus erythematosus, reactivation of cutaneous lupus erythematosus
Unknown	Erythema multiforme
**Musculoskeletal and connective tissue disorders**	
Unknown	Muscle spasm
**Renal and urinary disorders**	
Unknown	Acute renal failure, kidney disorders
**Reproductive system and breast disorders**	
Common	Erectile dysfunction
**General disorders and conditions at the place of administration**	
Unknown	Pyrexia, asthenia

In the event of adverse events, please report them via the VigiMed system, available on the Anvisa website.

10. OVERDOSE

There is no experience of overdose with Exforge HCT®. The main symptom of overdose with valsartan is probably pronounced hypotension with dizziness. Overdose with anlodipine can result in excessive peripheral vasodilation and possibly reflex tachycardia. Marked and potentially prolonged systemic hypotension has been reported, including shock with fatal outcome.

The occurrence of clinically significant hypotension due to anlodipine overdose requires active cardiovascular support measures, including frequent monitoring of cardiac and respiratory functions, elevation of the extremities, attention to circulating fluid volume and urinary elimination.

A vasoconstrictor may be useful in recovering vascular tone and blood pressure, as long as its use is not contraindicated.

If ingestion is recent, induction of vomiting or gastric lavage should be considered.

The administration of activated charcoal to healthy volunteers immediately or up to two hours after taking anlodipine showed a significant decrease in the absorption of anlodipine.

Intravenous calcium gluconate may be beneficial in reversing the effects of calcium channel blockers.

Both valsartan and anlodipine are unlikely to be removed by hemodialysis, while hydrochlorothiazide clearance is achieved by dialysis.

In case of poisoning, call 0800 722 6001 if you need further advice.

LEGAL PROVISIONS

Registration 1.0068.1082

Produced by: Siegfried Barbera S.L., Barberà del Vallès, Spain or Novartis Farma S.p.A., Torre Annunziata, Italy (see cartridge).

Imported and Registered by:
Novartis Biociências S.A.
Av. Prof. Vicente Rao, 90.

NOVARTIS

São Paulo – SP.
CNPJ: 56.994.502/0001-30
Made in Brazil.

® = Registered trademark of Novartis AG, Basel, Switzerland.

SALE ON PRESCRIPTION

This package leaflet was approved by Anvisa on 07/30/2024.

CDS 01.02.2021
NA
VPS12

VPS12 = Exforge HCT_Bula_Profissional

ANNEX III – PACKAGE LEAFLET

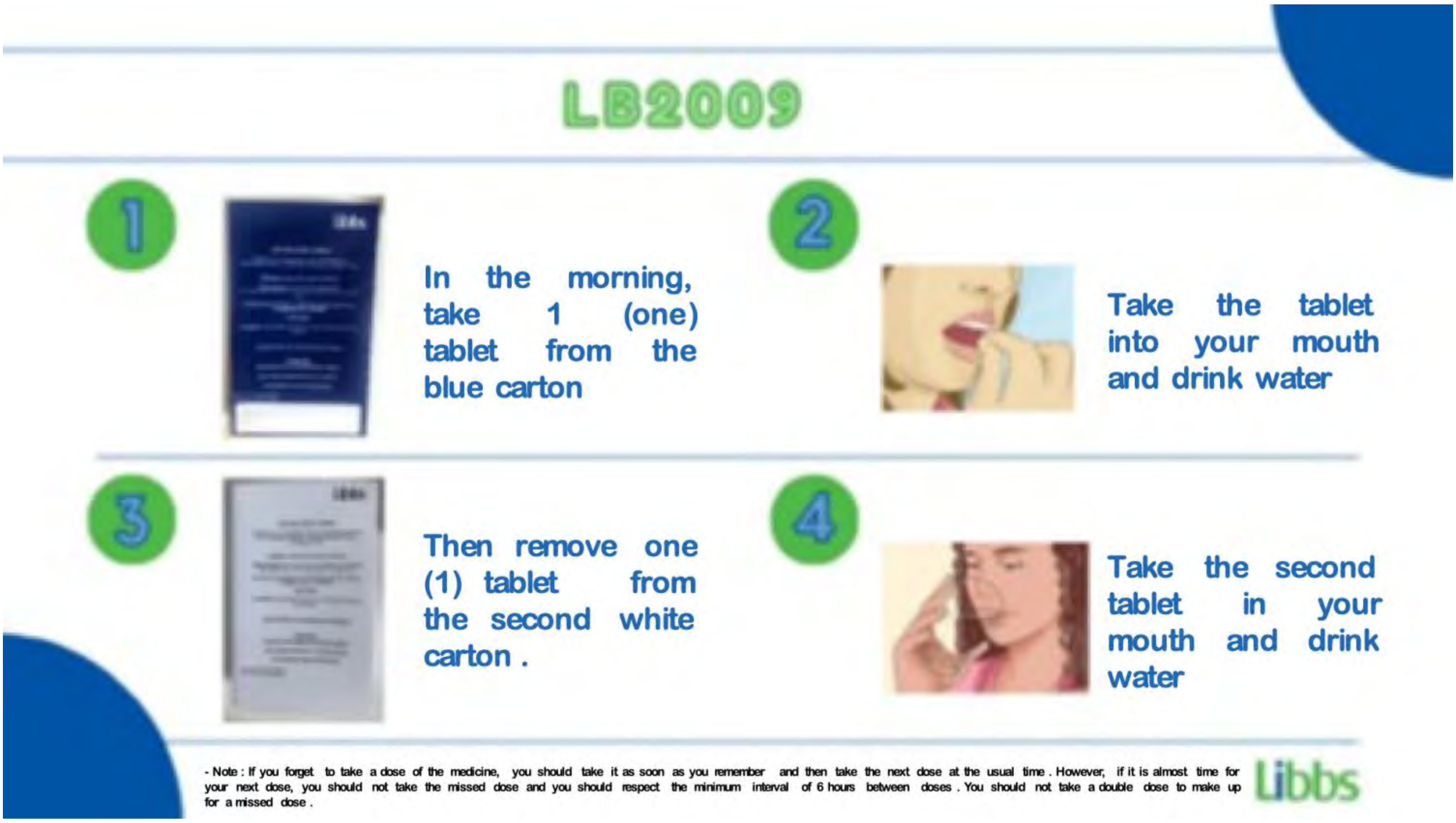

ANNEX IV – LDL CALCULATION (Low Density Lipoprotein) - Martin, 2013

LDL = CT - HDL - (Trig/NF)

Where:
LDL: Low
Density
Lipoprotein
CT: Total
Cholesterol
Trig: Triglycerides
NF: Novel Factor (must be evaluated in the table below).

To find the Non HDL value in the table below, consider:
Non HDL= total cholesterol – HDL.

Where:
HDL: High Density Lipoprotein

Note: This formula is only applicable for Triglyceride values >=400 mg/dL

Triglycerides [mg/dL]	Non-HDL Cholesterol [mg/dL]					
	<100	100-129	130-159	160-189	190-219	≥220
7-49	3.5	3.4	3.3	3.3	3.2	3.1
50-56	4	3.9	3.7	3.6	3.6	3.4
57-61	4.3	4.1	4	3.9	3.8	3.6
62-66	4.5	4.3	4.1	4	3.9	3.9
67-71	4.7	4.4	4.3	4.2	4.1	3.9
72-75	4.8	4.6	4.4	4.2	4.2	4.1
76-79	4.9	4.6	4.5	4.3	4.3	4.2
80-83	5	4.8	4.6	4.4	4.3	4.2
84-87	5.1	4.8	4.6	4.5	4.4	4.3
88-92	5.2	4.9	4.7	4.6	4.4	4.3
93-96	5.3	5	4.8	4.7	4.5	4.4
97-100	5.4	5.1	4.8	4.7	4.5	4.3
101-105	5.5	5.2	5	4.7	4.6	4.5
106-110	5.6	5.3	5	4.8	4.6	4.5
111-115	5.7	5.4	5.1	4.9	4.7	4.5
116-120	5.8	5.5	5.2	5	4.8	4.6
121-126	6	5.5	5.3	5	4.8	4.6
127-132	6.1	5.7	5.3	5.1	4.9	4.7
133-138	6.2	5.8	5.4	5.2	5	4.7

139-146	6.3	5.9	5.6	5.3	5	4.8

147-154	6.5	6	5.7	5.4	5.1	4.8
155-163	6.7	6.2	5.8	5.4	5.2	4.9
164-173	6.8	6.3	5.9	5.5	5.3	5
174-185	7	6.5	6	5.7	5.4	5.1
186-201	7.3	6.7	6.2	5.8	5.5	5.2
202-220	7.6	6.9	6.4	6	5.6	5.3
221-247	8	7.2	6.6	6.2	5.9	5.4
248-292	8.5	7.6	7	6.5	6.1	5.6
293-399	9.5	8.3	7.5	7	6.5	5.9
400-13975	11.9	10	8.8	8.1	7.5	6.7

STATISTICAL
ANALYSIS
PLAN

LB2009

Version 4.0 – September 20, 2024

CONFIDENTIAL

This document is confidential to Libbs Pharmaceuticals. Acceptance of this document constitutes the recipient's agreement that no unpublished information contained herein shall be published or disclosed without prior written approval.

National, phase III, multicenter, randomized, double-blind, controlled, parallel clinical study to evaluate the non-inferiority of the combination candesartan cilexetil 16mg + chlorthalidone 12.5mg + anlodipine 5mg compared to Exforge HCT® (valsartan 160mg + hydrochlorothiazide 12.5mg + anlodipine 5mg) in the treatment of systemic arterial hypertension in adults of both sexes.

Biostatistician in charge

Maura Gonzaga Lapa

Specialist in Clinical Research Statistics

Libbs Pharmaceuticals

Clinical research coordinator

Paula Bonilha Fernandes

Clinical Research Coordinator

Libbs Pharmaceuticals

Medical Consultant for the ARO (Academic Research Organization) - Hospital Israelita Albert Einstein

Dr. Patricia Oliveira Guimarães

Cardiologist

Hospital Israelita Albert Einstein

Sponsor

Libbs Pharmaceuticals

SUMMARY

List of abbreviations and definition of terms 4
Study overview 5
2. Sample Size Determination 12
3. General analysis definitions 15
3.1. Analysis populations 15
3.2. Arrangement of participants 16
3.3. Significance level and hypothesis testing 16
3.4. Interim analysis 16
3.5. Randomization 18
3.6. Data convention 18
3.6.1. Partial dates 18
3.6.2. Multiple assessments within visits 18
3.6.3. Calculation of time/duration of events 18
3.6.4. Addressing missing or invalid data 18
4. Study variables 19
5. Statistical methods 20
5.1. Demographic data and other baseline characteristics 20
5.2. Treatments 20
5.2.1. Exposure to study treatment 20
5.2.2. Prior and concomitant treatments 21
5.3. Efficacy 21
5.3.1. Primary Efficacy Analysis 21
5.3.1.1. Sensitivity analyses 22
5.3.2. Secondary Efficacy Analyses 22
5.4 Safety 23
5.5 Exploratory Analysis 23
6. Deviations from the protocol statistical plan 24
7. Statistical software 24
8. References 24
Versioning Table 26
Appendix - Results table templates 30

LIST OF ABBREVIATIONS AND DEFINITION OF TERMS

Abbreviation or term	Definition
ATC	Anatomical Therapeutic Chemical
SD	SD
DSMC	Independent Data Safety Monitoring Committee
CI	Confidence Interval
ITT	Intention-to-Treat Analysis Population
MedDRA	*Medical Dictionary for Regulatory Activities*
MAR	Missing at Random
MCMC	Markov Chain Monte Carlo Model
MMRM	Mixed Model for Repeated Measures
DBP	DBP
MNAR	Missing Not at Random
SBP	SBP
PMM	Pattern Mixture Model
PP	Per-Protocol Population
PT	Preferred Term
ICF	Informed Consent Form
SOC	System Organ Class
WHO-DD	*World Health Organization Drug Dictionary*

STUDY OVERVIEW

**Clinical Study Code**	LB2009
**Clinical Study Title**	National, phase III, multicenter, randomized, double-blind, controlled, parallel clinical study to evaluate the non-inferiority of the combination candesartan cilexetil 16mg + chlorthalidone 12.5mg + anlodipine 5mg compared to Exforge HCT® (valsartan 160mg + hydrochlorothiazide 12.5mg + anlodipine 5mg) in the treatment of systemic arterial hypertension in adults of both sexes.
**Version**	Version 4.0
**Document Date**	09/20/2024
**Clinical Study Population**	Participants of both sexes, ≥18 years old, and who have SBP measurements ≥ 140 mmHg and ≤180 mmHg and DBP ≥ 90 mmHg and ≤ 110 mmHg despite the use of dual antihypertensive therapy from different therapeutic classes for at least 8 weeks.
**Primary Objective**	To evaluate the efficacy through non-inferiority of the combination candesartan cilexetil 16mg + chlorthalidone 12.5mg + anlodipine 5mg compared to Exforge HCT® (valsartan 160mg + hydrochlorothiazide 12.5mg + anlodipine 5mg) in the mean change in SBP 12 weeks after starting treatment compared to baseline.
**Primary efficacy outcome**	The primary efficacy outcome is the mean change in SBP, measured at the research center, 12 weeks (± 4 days) after the start of treatment, compared to baseline (V0).
**Secondary Objectives**	To evaluate the change in DBP from baseline to 4, 8 and 12 weeks<br><br>To evaluate the change in SBP from baseline to 4 and 8 weeks<br><br>To assess the proportion of participants achieving target BP (SBP <140 and DBP <90 mmHg) 4, 8 and 12 weeks after starting treatment;<br><br>To assess the proportion of participants achieving SBP<120 mmHg 4, 8 and 12 weeks after starting treatment;<br><br>To assess the proportion of participants achieving SBP <140 mmHg) 4, 8 and 12 weeks after starting treatment;<br><br>To assess the proportion of participants achieving DBP<90 mmHg) 4, 8 and 12 weeks after starting treatment;<br><br>To assess the proportion of participants who show a reduction of greater than or equal to 20 mmHg in SBP 4, 8 and 12 weeks after starting treatment;<br><br>To assess the proportion of participants showing a reduction greater than or equal to 10 mmHg in DBP 4, 8

	and 12 weeks after starting treatment.
**Secondary Efficacy Outcomes**	Mean change in DBP, measured at the research center, from baseline (V0) to 4, 8 and 12 weeks (± 4 days);<br><br>Mean change in SBP, measured at the research center, from baseline (V0) to 4 and 8 weeks (± 4 days) ;<br><br>Proportion of participants who, after starting treatment, achieve target BP (SBP <140 and DBP <90 mmHg) at 4, 8 and 12 weeks (± 4 days);<br><br>Proportion of participants who, after starting treatment, achieve target SBP (SBP <120 mmHg) at 4, 8 and 12 weeks (± 4 days);<br><br>Proportion of participants who, after starting treatment, achieve target SBP (SBP <140 mmHg) at 4, 8 and 12 weeks (± 4 days);<br><br>Proportion of participants who, after starting treatment, reach the target SBP (SBP <140 mmHg) at 4, 8 and 12 weeks (± 4 days);<br><br>Proportion of participants who, after starting treatment, show a reduction of greater than or equal to 20 mmHg in SBP at 4, 8 and 12 weeks (± 4 days);<br><br>Proportion of participants who, after starting treatment, show a reduction of greater than or equal to 10 mmHg in DBP at 4, 8 and 12 weeks (± 4 days).
**Safety Objectives**	To assess the safety of treatments through the occurrence of adverse events and changes in laboratory tests, clinical/physical assessment and vital signs.
**Safety Outcomes**	Incidence of adverse events (AEs) recorded from the first dose of PSI up to 30 days after the end of the treatment; however, all adverse events occurring after the consent is obtained should be collected and reported;<br>Incidence of any AE occurring after the consent is obtained up to 30 days after the end of the treatment;<br><br>Proportion of participants with alterations in laboratory tests, considered to be clinically relevant according to the investigator's criteria, at the screening visit (V-1) and final visit (FV) of the study;<br><br>Proportion of participants with clinical or physical alterations, considered to be clinically relevant according to the investigator's criteria, at visits V1, V2 and FV in relation to baseline (V0);<br><br>Absolute change in vital signs measured at visits V1, V2 and FV in relation to the baseline visit (V0);<br><br>Note: Although only adverse events occurring after the use of the experimental/comparator drug will be used in the

	analysis that will evaluate the safety of the treatments, all adverse events occurring after the consent obtained will be collected and reported.
**Methodology**	The study includes a Screening Visit (V- 1), a Randomization Visit (V0), two Follow-up Visits (V1 and V2), a Final Visit (FV) and a telephone contact (CT).<br><br>The Screening visit (V-1) will be the first visit of the study and all procedures should only be carried out after consent has been obtained through the ICF. The purpose of this visit is to verify the eligibility criteria that may be assessed at that time.<br><br>The Randomization Visit (V0) will take place one week (+4 days) after the Screening Visit (V-1). At this visit, all eligibility criteria must be verified and, if the participant is eligible, he/she must be randomized and allocated to one of two treatment groups:<br><br>Group 1:<br><br>- Candesartan cilexetil 16mg + chlorthalidone 12.5mg + anlodipine 5mg;<br><br>Group 2:<br><br>- Exforge HCT® (valsartan 160mg + hydrochlorothiazide 12.5mg + anlodipine 5mg)<br><br>The Follow-up Visits (V1 and V2) will take place four and eight weeks (± 4 days) after the date of the Randomization Visit (V0). The purpose of this visit is to assess the efficacy and safety of the Investigational Product during the course of the study.<br><br>The Final Visit (FV) will take place twelve weeks (± 4 days) after the date of the Randomization Visit (V0). This visit aims to evaluate the effectiveness and safety of PSI after the end of treatment. This visit will be the last in-person visit of the study, therefore, the research participant must be instructed on the end of the study.<br><br>Telephone Contact (CT) will be made 30 (+7) days after the end of treatment with PSI. The purpose of this contact is to assess the possible occurrence of adverse events during this period.
**Inclusion Criteria**	1. Participants of both sexes of 18 years old or older;<br><br>2. Participants currently using dual anti-hypertensive therapy, from different therapeutic classes, for at least 8 weeks and non-responders to this treatment, defined as office measurements of SBP ≥ 140 mmHg and ≤180 mmHg and DBP ≥ 90 mmHg and ≤ 110 mmHg, assessed at the screening visit and randomization visit (both conditions are in accordance with the Brazilian Hypertension Guideline - 2020);

	3. Ability to understand and consent to their participation in this clinical study, expressed through the Informed Consent Form (ICF).
**Exclusion Criteria**	1. Any clinical observation finding (clinical/physical assessment and vital signs) that is interpreted by the investigator as a risk to the research participant's participation in the clinical study;<br><br>2. Any laboratory test finding that the investigating physician considers to be a risk to the research participant regarding their participation in the clinical study;<br><br>3. Participants suspected or diagnosed with COVID-19;<br><br>4. Known hypersensitivity to the components of the medicines used during the study or to medicines derived from sulphonamides;<br><br>5. Women who are pregnant or breastfeeding;<br><br>Menopausal women who do not agree to use effective contraceptive methods [oral contraceptive, injectable contraceptive, intrauterine device, hormonal implant, barrier methods, hormonal transdermal patch, vaginal ring and tubal ligation]; except those who are surgically sterile (bilateral oophorectomy or hysterectomy), those who have been menopausal , for at least one (1) year and participants who declare that they do not engage in sexual practices or do so in a non-reproductive manner;<br><br>7. Male participants who do not agree to use effective contraceptive methods:<br><br>a. contraceptive methods for the participant: condom, except for those who are surgically sterile (vasectomy) or for participants who are sexually abstinent during the study period or participants who declare that they do not engage in sexual practices or do so in a non-reproductive way;<br>OR<br>b. contraceptive methods for the female partner: oral contraceptive, injectable contraceptive, intrauterine device, hormonal implant, hormonal transdermal patch, tubal ligation, vaginal ring and barrier methods except for female partners who are surgically sterile (bilateral oophorectomy or hysterectomy), menopausal , for at least one (1) year;<br><br>8. Research participants who have taken part in clinical study protocols in the last 12 (twelve) months (CNS Resolution 251, of August 7, 1997, item III, sub-item J), unless the investigator believes that there may be a direct benefit to them;<br><br>9. Participants who have a relationship of up to the second degree or are related to collaborators or employees of the Sponsor and the Research Center;

	10. Participants with an estimated glomerular filtration rate (eGFR) of less than 45 ml/min /1.73m2 (calculated using the Chronic Kidney Disease Epidemiology Collaboration [CKD-EPI] equation) or end-stage renal disease;<br>11. Participants with a history of severe liver dysfunction;<br>12. Participants with a history of cardiogenic shock or heart failure with reduced ejection fraction, with left ventricular ejection fraction less than or equal to 50%;<br>13. History of symptomatic congestive heart failure classes II, III or IV according to the New York Heart Association (Annex I) and/or participants with a history of infarction, unstable angina or stroke in the last 6 months prior to the start of the study;<br>14. History of clinically relevant ventricular cardiac arrhythmias;<br>15. Participants with a history of obstructive coronary artery disease undergoing percutaneous or surgical coronary intervention;<br>16. Participants with a history of dementia syndrome;<br>17. History of alcohol or illicit drug dependence in the six months prior to the ICF date;<br>18. Participants who use prohibited medication as described in item 9.1;<br>19. Participants with a history of obstructive biliary disorders;<br>20. Participants with a history of refractory hypokalemia and/or conditions involving marked loss of potassium, hyperkalemia (with serum potassium levels above 5.5 mmol/L) and/or hyponatremia;<br>21. Participants with a history of symptomatic hyperuricemia (history of drop or uric acid calculation);<br>22. Participants with a history of secondary arterial hypertension;<br>23. Participants with a medical history of malignant neoplasia, without documentation of remission/cure.
**Experimental Treatment**	- Candesartan cilexetil 16mg + chlorthalidone 12.5mg + anlodipine 5mg;<br>- Pharmaceutical form: tablet;<br>- Dosage: administer one (1) tablet of the active experimental drug and then one (1) tablet of placebo of the comparator, orally, once a day, in the morning.
**Comparator Treatment**	- Exforge HCT® (valsartan 160mg + hydrochlorothiazide 12.5mg + anlodipine 5mg);<br>- Pharmaceutical form: coated tablet;

	- Dosage: administer one (1) tablet of the active comparator and then one (1) placebo tablet of the experimental drug, orally, once a day, in the morning.
**Number of Research Participants**	698 randomized research participants.
**Treatment Period**	Twelve weeks (± 4 days).
**Study Participation Duration**	Seventeen weeks (± 4 days), considering the period from the Screening Visit (V-1) to the Telephone Contact (TC) to be made 30 days after the end of treatment (+7).

1. Introduction

The purpose of this statistical analysis plan (SAP) is to ensure that data listings, summary tables and figures to be prepared, and statistical methodologies that will be used are complete and appropriate to allow valid conclusions to be drawn in relation to the study objectives.

This version of the SAP was developed in parallel with version 4.0 of the protocol, dated January 3, 2024. Any further changes to the protocol or CRF may require updates to the SAP.

This SAP describes the statistical analysis as envisaged at the time of study planning. The SAP will serve as a supplement to the study protocol and will replace it in case of discrepancies. In case of major differences between the study protocol and the SAP (e.g. changes in the analysis related to the primary outcome), a protocol amendment will be considered.

The SAP may be updated during the study, and will be finalized before the database is locked.

Study design:

After screening, verification of inclusion and exclusion criteria and consent obtained through the informed consent form, participants will be randomized in a double-blind fashion to one of the two treatment groups, and will receive guidance on administering one dose of the medication per day.

The study will follow the study schedule as per Figure 1:

Figure 1. Study schedule

Visit	V-1	V0	V1	V2	FV	TC
Week (days)	- 1 (+ 4)	0	4 (± 4)	8 (± 4)	12 (± 4)	30 days after end of treatment (+7)
Study phase	Screening	Randomization	Monitoring	Monitoring	Final	Follow-up
**Selection**						
Informed consent	x					
Evaluation of eligibility criteria	x	x				
Randomization		x				
**Treatment**						
Assessment of Previous/Concomitant/Prohibited Medications and Treatments	x	x	x	x	x	
Dispensing the PSI		x	x	x		
PSI return and checking / accounting			x	x	x	
Assessment of adherence to treatment			x	x	x	
**Efficacy and safety**						
Clinical / Physical Assessment / Vital signs	x	x	x	x	x	
Collection for laboratory tests[1]	x		x	x	x	
Dispensing male condoms[2]	x	x	x	x	x	
Assessment of occurrence of Adverse Events	x	x	x	x	x	x
Discontinuation Criteria		x	x	x		

2. Sample Size Determination

<u>Primary outcome</u>:

Mean change in SBP (ΔSBP) 12 weeks after starting treatment compared to baseline (FV – V0).

<u>Hypothesis</u>:

H0: Difference between effects is greater than 3 (H0: $\Delta T - \Delta C > 3$ mmHg)

H1: Difference between effects is not greater than 3 (H1: $\Delta T - \Delta C \leq 3$ mmHg), where:

- Δ T = Mean change in SBP in the Treatment group;
- Δ C = Mean change in SBP in the Control group;
- Margin of non-inferiority between changes: Δ = 3 mmHg.

Assumptions:

- Non-inferiority margin: Δ = 3 mmHg;
- SD of the reduction: SD = 14 mmHg;
- One-sided alpha = 0.05;
- Study power = 85%.

The study power was set slightly above 80%, a commonly used value, with the intention of ensuring the lowest possible type II error rate.

The non-inferiority margin of 3 mmHg was defined based on the minimum clinically relevant difference for assessing SBP (OJJI, 2019; N Engl J Med 2019;380:2429-39.). It is also noteworthy that the margin of 3 mmgHg in SBP preserves at least 50% of the magnitude of the effect observed in randomized clinical trials comparing combined triple therapy in a single pill versus usual treatment or combined double therapy (Table 1).

Table 1: Descriptive analysis of randomized studies including triple therapy in a single pill.

Study	Year	N	Treatment	Comparator	Titration	Δ SBP	Δ DBP	Highest SD SBP	Duration
Calhoun et al.	2009	2271	Anl/Val/HCTZ (10/320/ 25 mg)	Anl/Val (10/320 mg) or Val/HCTZ (320/25 mg) or Anl/HCTZ (10/25 mg)	Yes (*forced up-titration)*	8.2-6.2 (LSM)	5.3-3.3 (LSM)	14.5	9 weeks
Chrysant et al.	2010	2492	Anl/Olm/ HCTZ (10/40/ 25 mg)	Olm/Anl (40/10 mg) or Olm/HCTZ (40/25 mg) or Anl/HCTZ (10/25 mg)	No	9.6-7.1 (LSM)	6.7-3.8 (LSM)	15.1	12 weeks
Ferdinand et al.	2011	412	Anl/Ali/HCTZ (5/150/ 12.5 mg-10/300/25mg)	Anl/Ali (5/150 mg-10/300mg)	Yes (*forced up-titration)*	7.0 (LSM)	3.1 (LSM)	9.0	8 weeks
Maladkar et al.	2012	220	Anl/Tel/HCTZ (5/40/12.5 mg)	Tel/HCTZ (40/12,5 mg)	No	7.88	3.07	16.0	12 weeks

Higaki e col	2017	132	Tel/Anl/HCTZ (80/5/12.5mg)	Tel/HCTZ (80/12.5mg)	No	8.6	7.5	13.9	8 weeks
Webster et al.	2018	700	Tel/Anl/CLOR<br><br>(20-40/2.5-5.0/12.5-25)	At the discretion of the investigator/recommendation of SAH guidelines	Yes	8.8	4.6	11.6	6 months

Acronyms: Anl, anlodipine; Ali, aliskiren; CLOR, chlorthalidone; SD, SD; SAH, systemic arterial hypertension; HCTZ, hydrochlorothiazide; LSM, least square means; Olm, olmesartan; Tel, telmisartan; Val, valsartan.

The assumed SD was found in studies with similar designs, in particular in Smith, T. R, 2010, which followed participants for 8 weeks using low-dose Amlodipine/Valsartan/HCTZ (5/160/12.5mg) (Table 2 below) - and in Calhoun, D.A, 2009, which uses this SD value in the calculation of the sample size, with the objective of demonstrating a difference between triple therapy Anl/Val/HCTZ (10/320/25 mg) and dual therapies with the individual components.

Table 2 Mean changes from core baseline in MSDBP and MSSBP (mmHg)

	Low dose			High dose		
	n	MSDBP change, mean (SD)	MSSBP change, mean (SD)	n	MSDBP change, mean (SD)	MSSBP change, mean (SD)
Core baseline BP	619	99.0 (3.4)	152.1 (12.9)	627	98.8 (3.2)	151.7 (12.4)
Week 0	619	−14.0 (6.2)	−19.3 (12.3)	627	−13.8 (6.2)	−18.8 (11.9)
Week 4	616	−14.1 (7.0)	−18.7 (12.7)	621	−15.1 (6.8)	−19.5 (13.3)
Week 8	605	−16.4 (7.1)	−21.7 (13.6)	605	−17.1 (7.1)	−22.9 (13.7)
Week 13	595	−16.6 (7.0)	−23.0 (13.5)	587	−18.1 (6.7)	−24.5 (12.8)
Week 26	584	−16.9 (7.1)	−22.6 (13.3)	569	−18.5 (7.2)	−24.5 (13.1)
Week 39	564	−17.1 (7.2)	−22.6 (13.7)	549	−18.5 (6.9)	−24.0 (13.0)
Week 52	548	−17.4 (7.3)	−23.0 (13.3)	532	−18.7 (7.1)	−23.8 (13.2)
End-point*	616	−17.2 (7.7)	−22.1 (14.0)	621	−18.1 (7.5)	−22.8 (14.3)

*End-point is Week 52 or last-observation-carried-forward. BP, blood pressure; MSDBP, mean sitting diastolic blood pressure; MSSBP, mean sitting systolic blood pressure.

Estimation of the SD is planned in an interim analysis, with the aim of confirming the value estimated for the calculations.

<u>Conclusion:</u>

Using a Student's t-test, assuming the above premises, the non-inferiority of the Treatment in relation to the Control can be demonstrated with the evaluation of 314 participants in each group, with a power of 85% (total = 628 participants with available data).

Considering approximately 10% of losses/violations, 698 (349 per group) should be randomized.

In order to ensure that the number of participants with available data is reached, if the dropout rate is higher than expected, participants may be replaced.

<u>Formula used:</u> Sample size was calculated using the PASS 2021 software, version 21.0.2, considering the test of means for two independent groups and balanced samples. The sample size can be obtained by the following expression (Chow et al. (2003) pg 50),

$$n = \frac{(z_\alpha + z_\beta)^2 * \sigma^2}{(\varepsilon - \delta)^2}$$

where α is the one-tailed significance level; 1-β is the power of the test; Z_k is the (1-k)-th percentile of the standard Normal distribution; σ is the SD of the variable Δ SBP, assumed to be the same for both groups; $\varepsilon = \mu_2 - \mu_1$ is assumed equal to 0, and $\delta > 0$ is the non-inferiority margin.

3. General analysis definitions

3.1. Analysis populations

The following populations are defined for the analyses:

- Safety Analysis Population: analysis population in which all randomized participants who receive at least one dose of the investigational product will be included. Research participants will be classified in the safety analyses according to the medication they actually received during the study.
- Intention-to-Treat Analysis Population (ITT): All randomized participants will be included in the ITT analysis population. They will be classified according to the treatment group to which they were randomized, regardless of the treatment received.
- Per-Protocol (PP) Population: All research participants from the ITT population will be included in the Per-Protocol (PP) analysis population, except participants with:
    - Major protocol violations that interfere with efficacy of treatments, to be defined prior to freezing the database;
    - Lack of adherence to drug treatment (adherence less than 80% or greater than 120%) between V0 – V1, V1-V2 and/or V2 - FV.
    - Use of other antihypertensives after randomization;
    - Other protocol violations that may affect efficacy assessment, to be described and duly justified in the final/statistical report.

All protocol violations and major deviations will be documented during the study and presented in the statistical report.

Since the ITT population is considered to better reflect the behavior of the general population, and based on Bai, Anthony, 2021 and Wiens BL, Zhao, 2007, the primary efficacy analysis will consider mainly the ITT population and, additionally, the PP population. Secondary efficacy analyses will be performed for the ITT population, and safety analyses will be performed for the Safety population.

3.2. Arrangement of participants

The following data will be summarized in tables, by treatment group, when applicable, and overall.

- Number of participants screened;
- Number of participants who failed to be screened;
- Number of participants randomized to the study;
- Number (%) of participants in different study populations (% calculated from the ITT population), accompanied by justifications for exclusion of the participant from the PP population;
- Number (%) of participants who completed the study (based on the end-of-study case record page);
- Number (%) of participants who withdrew from the study and associated reasons (% calculated from the safety population);
- Number (%) of participants with protocol deviations and type of deviation (% calculated from the ITT population). At least the protocol deviations below will be described:
    - Participants randomized who do not meet any inclusion or exclusion criteria;
    - Participants who received the wrong treatment at any time during the study;
    - Participants who received prohibited concomitant medication.

3.3. Significance level and hypothesis testing

The primary outcome will be assessed using CI (90%), due to the assumptions used in calculating the sample size (considering that non-inferiority is established with a one-sided alpha error of 5%). All other results will be assessed using two-tailed hypothesis tests, with a significance level of 5%, and presented with effect estimates and their respective 95% confidence intervals. There will be no adjustments for multiple comparisons. Secondary outcomes will not be adjusted for multiplicity corrections.

3.4. Interim analysis

When 50% of the study participants reach the 12 weeks of follow-up, the SD value of the SBP changes (FV-V0) will be estimated, with the sole purpose of confirming the variability used in the sample size calculation. This estimate must occur in a blinded procedure (analysis of aggregated data). Since there will be no comparison between groups, no correction for alpha and beta expenditure will occur.

The study sample size may be recalculated using the estimated SD, if it is above the assumption applied for the sample size calculation. However, regardless of the new SD found during the interim analysis for the sample, the sponsor undertakes not to reduce the current sample size (698 randomized participants) and may only maintain or increase it.

The aggregated SD will be calculated using a mixed model adjusted for SBP change (ΔSBP), with baseline SBP value as a covariate, the 4th, 8th and 12th week as repeated measures and center as a random effect, using SAS statistical software. Using the Lsmeans command, estimates and standard errors (S.E.) of ΔSBP will be calculated for the studied time points. The SD for the primary outcome of 12 weeks will be calculated by multiplying the S.E. estimated by the model by the square root of the sample size (which should be approximately n = 349 participants):

SD = S.E. * root (n)

If SD> 14, the sample size should be recalculated using the SD estimated by the model. Otherwise, it will be maintained.

Independent Data Security Monitoring Committee (DSMC)

To enhance safety and integrity of study data, a blinded review committee of independent experts will be convened to periodically review accumulated safety data for the study. Safety reviews for the purpose of evaluating the data by the review committee will occur after approximately 25% and 50% of participants, approximately half in each treatment group, have completed the 12-week follow-up of the study. Unscheduled meetings may be requested if any concerns about participant safety arise during the course of the clinical trial (e.g., a series of adverse events attributed to the same event, such as emergency or hypertensive crisis). The committee's bylaws, which outline its responsibilities and composition, are described in the document known as the Data and Safety Committee Manual.

At a minimum, all of the following accumulated data will be reviewed during DSMC analyses:

Incidence of adverse events and serious adverse events;

Proportion of participants with changes in laboratory tests, considered clinically relevant according to the investigator's criteria, at 4, 8 and 12 weeks;

Proportion of participants with clinical or physical changes, considered clinically relevant according to the investigator's criteria, at 4, 8 and 12 weeks;

Absolute change in vital signs measured at 4, 8 and 12 weeks in relation to the baseline visit (V0);

Missing safety data rate of the above mandatory items during the monitoring visits;

The DSMC may recommend suspension or termination of the study for safety reasons if any of the following comparative scenarios between treatment and control groups occur during the safety analyses:

Increased mortality in one of the groups with a two-tailed alpha threshold <0.01 for superiority;

Increased incidence of serious adverse events in one of the groups with a two-tailed alpha threshold <0.01 for superiority;

All details regarding analyses and the committee are explained in the DSMC manual.

Safety data reports for DSMC analyses and data reports for the interim analysis of possible sample recalculation will be generated through the electronic data collection system in a blinded manner, i.e., without determining the allocated group (test or control group). The partner responsible for implementing the electronic clinical record and for managing the clinical trial data will be informed about the unblinded people in the study and, if breaking the blinding is required for safety reasons, the code will be opened for the unblinded team only.

3.5. Randomization

Participants who meet all eligibility criteria and sign the informed consent form will be randomized in a 1:1 ratio to Groups 1 and 2. The randomization sequence will be generated by validated software, with size 4 blocks.

Any randomization code break that occurs before formal uncoding will be documented in the final study report, and the impact on results will be assessed.

3.6. Data convention

3.6.1. Partial dates

Partial dates will be used in calculation of time between dates, if necessary and appropriate, considering the following: in the lack of month information, the first month of the year will be considered; in the lack of day information, the first day of the month will be considered.

3.6.2. Multiple assessments within visits

In case of multiple assessments for a variable within the same visit, the average between assessments will be considered for analysis purposes.

3.6.3. Calculation of time/duration of events

Intervals between dates, when expressed in years, will be calculated as below, exemplified for calculating the age of participants:

Age = int ((date of informed consent - date of birth + 1) / 365.25)

If the time is calculated in months, the duration period, in days, will be divided by 30.44.

3.6.4. Approach to missing or invalid data. In a sensitivity analysis, in the lack of post-baseline data, data imputation will be used, applicable to SBP assessments, in primary and secondary efficacy, for the ITT population.

For multiple data imputation, techniques based on Pattern Mixture Models (PMM) will be applied (Ratitch et al., 2011). Intermittent missing values will be imputed using the Markov chain Monte Carlo (MCMC) methodology, which assumes a multivariate Normal distribution over all variables included in the imputation model. The MI procedure in SAS will be used for this purpose, and this first step of multiple imputation is planned to be repeated 50 times, generating different data sets with a monotonic missing data structure. The imputation is based on the missing at random (MAR) assumption (Little and Rubin, 1987), i.e., it is assumed that the missing data follow the same pattern as the other participants with complete data.

Missing data resulting from dropouts will be imputed using a method for monotonic data, also based on the MAR assumption. Therefore, for each of the data sets created, the MI procedure in SAS will be used to impute missing values based on a sequential procedure. Participants with the first missing value occurring at V1 will have their missing value replaced by an imputed value from a regression model with treatment group, baseline BP value, and other explanatory variables from the analysis model. In the next step, patients with a missing FV value will have it replaced by a value imputed from a regression model with treatment group, V1 BP value, and other explanatory variables from the analysis model.

Missing data from participants who permanently discontinue the PSI due to use of another antihypertensive medication in a manner not permitted by the protocol will be imputed using the LOCF method from week 8 onwards, as they cannot be classified as missing not at random (MNAR).

There will be no data imputation for analyses of categorical variables.

Following the guidelines of ICH-E9 (Statistical Principles for Clinical Trials), if any data is statistically and clinically identified as spurious, the analyses will be performed with and without this data. If a difference is found between the results of the two analyses, an assessment of the interference of the spurious data at the end of the study will be presented in the final report.

4. Study outcomes

At least the following data will be collected in a medical record:

Demographic data and baseline characteristics:

- Demographic data and baseline characteristics: age, sex, race, education;
- Vital signs (BP, heart rate and respiratory rate);
- General physical assessment (weight, BMI, respiratory and cardiovascular system);
- Laboratory tests;
- History of cardiovascular diseases, respiratory diseases, kidney diseases, urological, endocrine, neurological diseases etc.;
- Medical history of family antecedents (father, mother and siblings), such as early coronary disease or hypertension;
- Use of contraceptive methods;
- History of smoking and alcoholism.

Treatments:

- History of treatments and/or surgical procedures;
- Previous medications, according to the ATC (Anatomical Therapeutic Chemical) classification of the WHO-DD (World Health Organization Drug Dictionary6);
- Concomitant medications, according to the ATC classification of the WHO-DD dictionary.

Efficacy: BP in a sitting position;

Safety:

- Adverse events, according to MedDRA terminology;
- Weight and vital signs - temperature, systolic and DBP and heart rate;
- Physical exam;
- Laboratory tests.

5. Statistical methods

Statistical analyses will be performed after freezing the data and blocking the data collection system (clinical record), using SAS® software (version 9.4 or later).

5.1. Demographic data and other baseline characteristics

Demographic characteristics, pre-existing clinical conditions, medical history, and previous treatments reported at baseline will be summarized by treatment group,

considering the ITT population. Efficacy variables observed at baseline will not be described in this section. Regarding safety, only the variables weight, vital signs and physical examination at visit 1 will be described as baseline characteristics. The others will be presented in the safety analysis.

Descriptive statistics will be calculated to summarize the data according to the type of each variable: absolute and relative frequency, and 95% CI, when appropriate, will be used for categorical and ordinal numerical variables, and number of valid observations, mean, SD, median, interquartile range, minimum and maximum values will be calculated for continuous numerical variables.

5.2. Treatments

5.2.1. Exposure to study treatment

The number of participants using the study medication, as well as the exposure time, number of doses, daily exposure dose and adherence to treatment will be summarized by treatment group actually received in the safety population.

Exposure time will be calculated as the time between start and end dates of drug use, including possible temporary interruptions (Date of last administration - Date of first administration + 1).

Adherence to study treatment will be calculated as a percentage, as: (Amount of use*100) / Expected amount of use.

Adherence will be summarized by frequency and percentage of participants in relation to the total safety population, and the other variables will be described by number of participants with information, mean, SD, median, and minimum and maximum values.

5.2.2. Prior and concomitant treatments

Prior and concomitant treatments will be summarized by study group in the ITT population. Prior treatments are those initiated and terminated before the first administration of study medication. Concomitant treatments are those initiated on or after the first administration of study medication, or initiated before the first administration of study medication and continued after the first dose.

Frequency and percentage of participants using a medication will be presented according to the ATC classification of the WHO-DD dictionary.

Separate listings describing, by participant, the use of prior, concomitant and undefined medications (without valid start and/or end date) will also be provided.

5.3. Efficacy

SBP and DBP measurements will be summarized using appropriate descriptive statistics, as defined in Section 5.1, in tables and graphs, by time point and by treatment group. The number of participants achieving target pressures and respective proportions will be described and displayed in tables and graphs, at each time point and for each treatment.

The primary efficacy analyses will be performed considering ITT and PP populations. Secondary efficacy analyses will be performed for the ITT population, and safety

analyses will be performed for the Safety population.

5.3.1. Primary Efficacy Analysis

The primary hypothesis of efficacy is the non-inferiority of Group 1 in relation to Group 2 (control), in the mean change of SBP (ΔSBP), 12 weeks after the start of treatment (FV) in relation to the baseline (V0).

With ΔT and ΔC being the changes obtained in SBP (FV – V0) of Groups 1 and 2, respectively, the conclusion of non-inferiority will be given if the upper limit of the CI (90%) of the difference between the groups does not exceed 3 mmHg:

$$\Delta T - \Delta C \leq 3$$

The data will be analyzed and the confidence interval constructed using a mixed model for repeated measures (MMRM), with a random effect of center, fixed effect of treatment, moment and moment/treatment interaction, assessment moments as repeated measures and SBP values at V0 as a covariate. The “unstructured” covariance structure will be used for the MMRM model, which may be replaced by a different covariance pattern, if appropriate. The restricted maximum likelihood (REML) estimation method will be used to adjust the model. This analysis will be performed using the ITT population.

5.3.1.1. Sensitivity analyses

As sensitivity analyses, the MMRM model described above will be repeated using:

- ITT population and CI (95%);
- PP population and CI (90%);
- PP population and CI (95%);

In addition, participants without post-baseline data will not contribute to the analyses performed with the MMMR approach, but will contribute to the sensitivity analysis with data imputation defined in Section 3.6.4.

5.3.2. Secondary Efficacy Analyses

Analyses of SBP and DBP change (ΔSBP and ΔDBP) will be performed using the same MMRM model described for the primary efficacy analysis.

In addition to descriptive measures of SBP and DBP for each time point and for each treatment group, the results of the analysis model for the effects of treatment, time point and interaction between treatment/time point will be presented. Adjusted mean estimates and 95% two-sided confidence intervals for ΔSBP and ΔDBP will be established for the difference between treatments (ΔT – ΔC) at weeks 4, 8 and 12.

The MMRM model proposed for analyses of the primary objective and secondary outcomes related to numerical changes in SBP and DBP is used under the assumption of data normality. If normality is not confirmed, the generalized linear model proposed in Section 6 will be used as sensitivity analysis.

The following proportions of participants will be compared between treatment groups at Weeks 4, 8 and 12, using the Chi-Square test:

- Participants achieving target BP (SBP <140 and DBP <90 mmHg);
- Participants achieving SBP <120 mmHg;
- Participants achieving SBP <140 mmHg;
- Participants achieving DBP <90 mmHg;
- Participants with a reduction greater than or equal to 20 mmHg in SBP;
- Participants with a reduction greater than or equal to 10 mmHg in DBP.

The proportions will be calculated as follows, for each post-baseline week:

- Numerator: number of participants achieving the target at the time;
- Denominator: number of participants in the ITT population;

5.4 Safety

The following safety outcomes will be considered:

- Incidence of adverse events (AE) recorded from the first dose of PSI up to 30 days after the end of the treatment;
- Incidence of any AE occurring after the consent obtained through the ICF up to 30 days after the end of the treatment ;
- Proportion of participants with changes in laboratory tests considered clinically relevant according to the investigator's criteria, at the baseline visit (V0) and at the final visit (FV) of the study;
- Proportion of participants with clinical or physical changes, considered relevant according to the investigator's criteria, at visits V1, V2 and FV in relation to the baseline (V0);
- Absolute changes in vital signs measured at visits V1, V2 and FV in relation to the baseline visit (V0)

Number and percentage of participants with adverse events will be presented along with the respective 95% CIs for each outcome, by treatment group, and according to severity, intensity, causality with study drugs, action taken and outcome.

Adverse events reported during the treatment period will be coded using the MedDRA (Medical Dictionary for Regulatory Activities) dictionary. Serious adverse events or those that led to permanent discontinuation of treatment will be listed. All adverse events recorded in the study will be organized in a list, using the MedDRA PT and SOC terms, containing information on the date of onset, severity, intensity, causality, action taken, outcome, with identification of the participant and treatment group.

Vital signs, physical assessment results, and laboratory tests will be summarized using descriptive statistics for each treatment group at each assessment time. The absolute change in vital signs from visit 1, visit 2, and final visit to visit 0 will be calculated and displayed by treatment group.

The percentages of participants with clinically relevant changes will be summarized by

treatment group:

- in laboratory tests, from final visit to visit -1;
- in laboratory tests from visits 1 and 2;
- in clinical and physical assessments from visits 1, 2, and from final visit to visit 0;

5.5 Exploratory Analysis

As an exploratory analysis, the difference between treatments in relation to SBP and DBP changes at 4, 8 and 12 weeks will be assessed using the nonparametric Mann-Whitney test.

6. Deviations from the protocol statistical plan

The statistical methods planned for efficacy analysis are parametric methods that assume a certain probability distribution for the data. For each adjusted model, the assumption about data distribution will be evaluated and, if the assumption is not confirmed, data transformations may occur.

The MMRM model proposed for analyses will be used under the assumption of normality of SBP and DBP change values. To verify normality, the Univariate procedure of the SAS statistical software will be used, observing the result of the Shapiro-Wilk test (normality rejected if $p < 0.05$).

If normality is not confirmed, the data will be transformed to make them positive to allow the evaluation of other probability distributions and use the generalized linear model, with fixed effects of treatment, moment, moment/treatment interaction and center, with moment declared as a repeated measurement and SBP/DBP values at V0 as a covariate, using the distribution that best fits the data, through the GENMOD procedure of SAS software. For this transformation, the smallest value among the changes, in module, is planned to be added to all data.

The Gamma distribution will be assumed as an alternative assumption to the Normal distribution, and its adherence to data will be verified through the Univariate procedure of the SAS statistical software.

The assumptions will be made about primary and secondary numerical variables that involve statistical models, that is, change of SBP (SBP) and DBP (DBP). The other outcomes (proportions) do not require evaluation in relation to data distribution.

Any alternative statistical method to be used in the data analysis will be duly described and justified in the final study report.

7. Statistical software

For all statistical analyses, SAS software version 9.4 (or later) will be used.

8. References

Bai, Anthony D., et al. "Intention-to-treat analysis may be more conservative than per protocol analysis in antibiotic non-inferiority trials: a systematic review." BMC medical

research methodology 21.1 (2021): 1-10.

Calhoun, David A., et al. "Triple antihypertensive therapy with amlodipine, valsartan, and hydrochlorothiazide: a randomized clinical trial." Hypertension 54.1 (2009): 32-39

Enders, Craig K. *Applied missing data analysis*. Guilford press, 2010.

Onakpoya, Igho. "Comparative effectiveness of combination treatment for hypertension in black Africans." *BMJ Evidence-Based Medicine* 25.3 (2020): 112-112.

Smith, T. R., et al. "Combination therapy with amlodipine/valsartan in essential hypertension: a 52-week, randomised, open-label, extension study." International journal of clinical practice 64.10 (2010): 1367-1374.

Spratt, Michael, et al. "Strategies for multiple imputation in longitudinal studies." *American journal of epidemiology* 172.4 (2010): 478-487.

Wiens BL, Zhao W. The role of intention to treat in analysis of noninferiority studies. Clinical Trials. 2007;4(3):286-291. doi:10.1177/1740774507079443.

Ratitch et al., 2011. " O'Kelly, Michael, and Bohdana Ratitch. Clinical trials with missing data: a guide for practitioners. John Wiley & Sons, 2014.

Ratitch, B, O’Kelly, M. Implementation of Pattern-Mixture Models Using Standard SAS/STAT Procedures. Proceedings of PharmaSUG2011 Conference – Paper SP04. http://www.pharmasug.org/proceedings/2011/SP/PharmaSUG-2011-SP04.pdf

Little e Rubin, 1987. " Little, Roderick JA, and Donald B. Rubin. Statistical analysis with missing data. Vol. 793. John Wiley & Sons, 2019.

Versions

SAP		Amendments		
**Version No.**	**Date**	**Amendm. No**	**SAP section**	**Summary of main changes**
1	3/15/2022	-	-	N.A (issuance)
2	2/17/2023	1	Overview	Overview amended in accordance with amendment 1 of the protocol
2	2/17/2023	1	Study schedule	Schedule amended as per protocol amendment 1 (including visits at weeks 4 and 8 in place of week 6)
2	2/17/2023	1	3.1. Analysis Populations - PP -	Predefined items removed in relation to the PP population to adapt to the protocol.
2	2/17/2023	1	3.1. Analysis Populations - PP - Breachers - Lack of adherence to drug treatment	(adherence less than 80% or greater than 120%) between V0 – V1, V1-V2 and/or V2~~1~~ - FV - Changed to include visit added to the study design.
2	2/17/2023	1	3.4. Interim Analysis	Clarifications included regarding calculation of sample size by estimating the SD in the interim analysis: Rule, formula, person responsible for calculation, guarantee of blinding, guarantee there will be no reduction in sample size.
2	2/17/2023	1	3.4. Interim Analysis	Independent Data Safety Monitoring Committee included
2	2/17/2023	1	3.6. Data convention - Missing or invalid data addressed	Change of outcomes for sensitivity analysis with imputation (only SBP change in MMRM model); Added LOCF for handling missing MNAR data

2	2/17/2023	1	5.3. Primary efficacy analysis	1) Formula changed to $\Delta T - \Delta C \leq 3$ and text rewritten, both for better understanding; 2) Mixed model covariance structure added as Unstructured
2	2/17/2023	1	5.3. Secondary efficacy analyses	Increased text detail
2	2/17/2023	1	5.4. Safety	V2 added to analyzes
2	2/17/2023	1	5.5. Exploratory Analysis	Nonparametric test included as exploratory analysis for comparison between groups
2	2/17/2023	1	6. Deviations from the protocol statistical plan	Included text detailing normality test and GLM alternative in case of not meeting normality.
2	2/17/2023	1	Appendix	Included models of some Tables that will be presented in the report.
**SAP**		**Amendments**		
**Version No.**	**Date**	**Version No.**	**Date**	**Version No.**
3	4/14/2023	2	Overview	Overview amended in accordance with amendment 2 of the protocol
3	4/14/2023	2	2. Sample Size Determination	Row of Table 1 of articles corrected, as it mentioned comparison of two Triple associations.
3	4/14/2023	2	5.3. Efficacy	Added sensitivity analysis in case normality in data is not reached
3	4/14/2023	2	6. Deviations from the protocol statistical plan	GLM alternative text corrected in case it does not meet normality.
3	4/14/2023	2	3.4. Interim Analysis	Changes made to objectives of the safety committee (DSMC)
3	4/14/2023	2	3.4. Interim Analysis	Added explanatory text on how blind reports will be generated for interim analyses.
**SAP**		**Amendments**		
**Version No.**	**Date**	**Version No.**	**Date**	**Version No.**
4	9/20/2024	3	Participants – people responsible for the study, page 2	Clinical Research Manager Angélica Csipak replaced by Paula Bonilha Fernandes, Clinical Research Coordinator

4	9/20/2024	3	List of Abbreviations and Definitions of Terms	ICF included in the list
4	9/20/2024	3	OVERVIEW	Overview amended in accordance with amendment 3 of the protocol
4	9/20/2024	3	3.4. Interim Analysis	Adjustment of the number of participants calculated as 50% to be evaluated in the interim analysis: from 314 to 349 (includes the forecast of 10% dropout)
4	9/20/2024	3	3.5. Randomization	Correction of the text removing stratification by center, as it was not defined in the protocol.
4	9/20/2024	3	3.6.4. Missing or invalid data addressed	Where it reads: Missing data from participants who permanently discontinue the PSI due to use of another antihypertensive medication will be imputed using the LOCF method, It should read: Missing data from participants who permanently discontinue the PSI due to use of another antihypertensive medication **in a manner not permitted by the protocol** will be imputed using the LOCF method.<br>Due to the permission introduced in version 4 of the protocol.
4	9/20/2024	3	5.2.1 Exposure to study treatment	Text removed to adapt to the protocol amendment: Adherence to study treatment will be calculated as a percentage, as: (Amount of use*100) / Expected amount of use. ~~The calculation must be made up to one day before the return date.~~<br>~~In case the participant uses the medication on the day of return (intermediate visits - V1 and V2), the calculation will be added to the next visit, except for the final visit.~~
4	9/20/2024	3	5.2. Treatments and 5.3. Efficacy	For better organization and understanding of the SAP, the sections were subdivided and numbered: 5.2.1. Exposure to treatment and 5.2.2. Prior and concomitant treatments;<br>5.3.1. Primary Efficacy Analysis, 5.3.1.1. Sensitivity analyses and

				5.3.2. Secondary Efficacy Analyses
4	9/20/2024	3	8. References	Inclusion of bibliographic reference:<br>Ratitch, B, O'Kelly, M. Implementation of Pattern-Mixture Models Using Standard SAS/STAT Procedures.<br>Proceedings of PharmaSUG2011 Conference – Paper SP04.<br>http://www.pharmasug.org/proc e edings/2011/SP/PharmaSUG-2011-SP04.pdf

Appendix - Results table templates

1. Primary Efficacy Results

Table 4.1. SBP, by moment and treatment (ITT).

SBP (mmHg)	V0 (Baseline)	V1 (Week 4)	V2 (Week 8)	FV (Week 12)
**Treatment A (n = X)**				
Mean ± SD	0 ± 0	0 ± 0	0 ± 0	0 ± 0
Median	0.0	0.0	0.0	0.0
Q1 - Q3	0 - 0	0 - 0	0 - 0	0 - 0
Minimum - Maximum	0 - 0	0 - 0	0 - 0	0 - 0
Total participants	0	0	0	0
**Treatment B (n = X)**				
Mean ± SD	0 ± 0	0 ± 0	0 ± 0	0 ± 0
Median	0.0	0.0	0.0	0.0
Q1 - Q3	0 - 0	0 - 0	0 - 0	0 - 0
Minimum - Maximum	0 - 0	0 - 0	0 - 0	0 - 0
Total participants	0	0	0	0

Table 4.2. Change of SBP, by moment and treatment (ITT).

ΔSBP (mmHg)	V1 (Week 4)	V2 (Week 8)	FV (Week 12)
**Treatment A (n = X)**			
Mean ± SD	0 ± 0	0 ± 0	0 ± 0
Median	0.0	0.0	0.0
Q1 - Q3	0 - 0	0 - 0	0 - 0
Minimum - Maximum	0 - 0	0 - 0	0 - 0
Total participants	0	0	0
**Treatment B (n = X)**			
Mean ± SD	0 ± 0	0 ± 0	0 ± 0
Median	0.0	0.0	0.0
Q1 - Q3	0 - 0	0 - 0	0 - 0
Minimum - Maximum	0 - 0	0 - 0	0 - 0
Total participants	0	0	0
Value p[1] (Treatment)		p = 0.000	
Value p[1] (Moment)		p = 0.000	
Value p[1] (Moment*Treatment)		p = 0.000	
**Difference between treatments**			
A - B - 90% CI (bilateral)	[0.0 ; 0.0]	[0.0 ; 0.0]	[0.0 ; 0.0]
A - B - 95% CI (bilateral)	[0.0 ; 0.0]	[0.0 ; 0.0]	[0.0 ; 0.0]

[1] MMRM with center as random effect
